# Exploring the Molecular Mechanism of Liuwei Dihuang Pills for Treating Diabetic Nephropathy by Combined Network Pharmacology and Molecular Docking

**DOI:** 10.1155/2021/7262208

**Published:** 2021-09-13

**Authors:** Gaoxiang Wang, Lin Zeng, Qian Huang, Zhaoqi Lu, Ruiqing Sui, Deliang Liu, Hua Zeng, Xuemei Liu, Shufang Chu, Xinhui Kou, Huilin Li

**Affiliations:** ^1^Shenzhen Traditional Chinese Medicine Hospital Affiliated to Nanjing University of Chinese Medicine, Shenzhen 518033, Guangdong, China; ^2^Department of Endocrinology, Shenzhen Traditional Chinese Medicine Hospital, Shenzhen 518033, Guangdong, China; ^3^The Fourth Clinical Medical College of Guangzhou University of Chinese Medicine, Shenzhen 518033, Guangdong, China; ^4^Department of Oncology, Lishui District Traditional Chinese Medicine Hospital, Nanjing 211200, Jiangsu, China

## Abstract

**Background:**

Diabetic nephropathy (DN) is a common and serious complication of diabetes, but without a satisfactory treatment strategy till now. Liuwei Dihuang pills (LDP), an effective Chinese medicinal formula, has been used to treat DN for more than 1000 years. However, its underlying mechanism of action is still vague.

**Methods:**

Active compounds and corresponding targets of LDP were predicted from the TCMSP database. DN disease targets were extracted from the OMIM, GeneCards, TTD, DisGeNET, and DrugBank databases. Subsequently, the “herbal-compound-target” network and protein-protein interaction (PPI) network were constructed and analyzed via the STRING web platform and Cytoscape software. GO functional and KEGG pathway enrichment analyses were carried out on the Metascape web platform. Molecular docking utilized AutoDock Vina and PyMOL software.

**Results:**

41 active components and 186 corresponding targets of LDP were screened out. 131 common targets of LDP and DN were acquired. Quercetin, kaempferol, beta-sitosterol, diosgenin, and stigmasterol could be defined as five crucial compounds. JUN, MAPK8, AKT1, EGF, TP53, VEGFA, MMP9, MAPK1, and TNF might be the nine key targets. The enrichment analysis showed that common targets were mainly associated with inflammation reaction, oxidative stress, immune regulation, and cell apoptosis. AGE-RAGE and IL-17 were the suggested two significant signal pathways. Molecular docking revealed that the nine key targets could closely bind to their corresponding active compounds.

**Conclusion:**

The present study fully reveals the multicompound's and multitarget's characteristics of LDP in DN treatment. Furthermore, this study provides valuable evidence for further scientific research of the pharmacological mechanisms and broader clinical application.

## 1. Introduction

Diabetes mellitus, like COVID-19, is a wicked problem [1]. It may affect 693 million adults by 2045, according to the prediction of the International Diabetes Federation [2]. Diabetes mellitus has emerged as the leading cause of diabetic nephropathy (DN), which is the leading cause of the end-stage renal disease (ESDR) [3]. Epidemiological studies have shown that more than 30% of diabetic patients may develop DN [4]. In some parts of the world, ESDR caused by DN accounts for over 50% of renal replacement therapeutic patients [5]. The current therapies strategies for DN mainly include controlling blood glucose, reducing proteinuria, and managing merging symptoms [6]. However, the understanding of DN continues to increase, and current treatment methods for DN are still not effective enough. Many patients with DN have a poor prognosis, especially those with advanced DN are still unsatisfactory [7]. Therefore, novel therapeutic drugs for DN are urgently required. Fortunately, Liuwei Dihuang pills (LDP) may be a potential complementary and alternative therapy medicine for DN.

LDP, a classical prescription first described in Xiaoer Yaozheng Zhijue, has been used to treat DN for more than 1000 years by Chinese people. Qian Yi formulated LDP during the Song dynasty. It is composed of 6 herbs, including Rehmanniae Radix Preparata (Shudihuang), Cortex Moutan (Mudanpi), Rhizoma Dioscoreae (Shanyao), *Cornus officinalis* (Shanzhuyu), *Alisma* (Zexie), and *Poria Cocos* (Fuling). LDP is considered to have the efficacy of nourishing the yin and kidney. In recent years, more and more researchers' interest has been focused on LDP to treat DN. Xu et al. [8] have reported that LDP can protect glomerular mesangial cells and prevent renal fibrosis in the treatment of rats of DN. Lin et al. [9] have documented that Western medicine has better therapeutic efficacy in treating DN when combined with LDP. Shi et al. [10] also have found that LDP has a practical therapeutic effect on diabetic nephropathy. A meta-analysis including 14 RCT studies and 918 study participants has reported that LDP categorized formulas are safe and effective in treating DN proteinuria [11]. However, because of the characteristics of multicompound and multitarget, exploring the underlying molecular mechanism of traditional Chinese medicine (TCM) through cellular or animal studies is relatively tricky [12]. Until now, the pharmacological mechanism of LDP in DN treatment is still vague, which greatly limits its extensive application.

**Figure 1:**
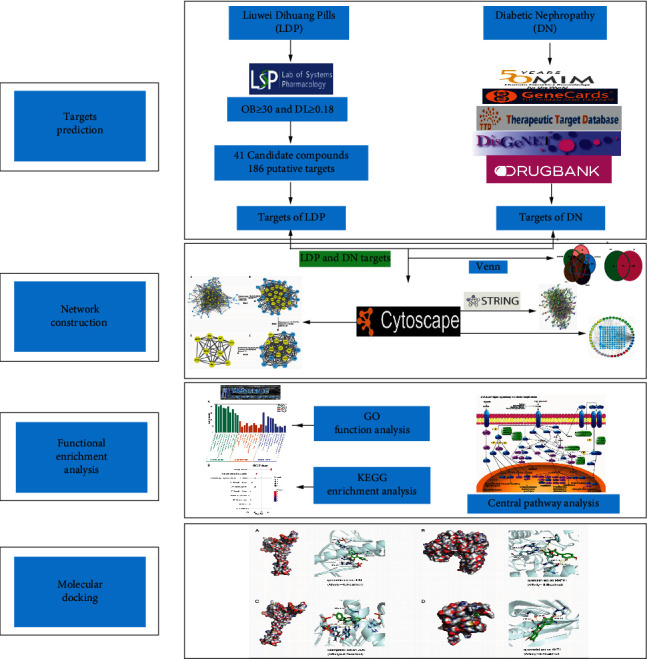
The flowchart of LDP in treating DN.

Because of the holism concept of TCM, Chinese medicinal formulas are usually treated diseases through several components and targets rather than a single one [13]. Network pharmacology can abstract the interaction relationship of drugs and targets into a network model and study them via a holistic perspective, consistent with the concept of holism in TCM [14]. In recent years, it has been recognized as an efficient method to study TCM. Therefore, our study was set out to reveal the mechanism of LDP in treating DN via the pharmacology network and molecular docking combination approach. It can provide valuable evidence for further basic research and clinical applications. The flowchart of network pharmacology research on LDP in treating DN is shown in Figure 1.

## 2. Methods

### 2.1. Herbal Compounds and Corresponding Targets in LDP Extraction

Traditional Chinese Medicine System Pharmacology Database (TCMSP, https://tcmspw.com/tcmsp.php/), a systematic pharmacology database, provides information about the active herbal components and related targets [15]. We searched the TCMSP database to acquire the active compounds in LDP. Oral bioavailability (OB) means the rate and percentage of pharmaceutical agents orally absorbed into the systemic circulation [16]. Drug-likeness (DL) is a concept based on existing drugs' physical and chemical properties and can be used to estimate compounds whether reaching the conditions to become new drugs [16]. The active compounds in LDP were further screened with the criteria that OB ≥ 30% and DL ≥ 0.18. Then, we also screened the targets related to these herb compounds from the TCMSP database. Taking “Reviewed” and “Human organisms” for filtering conditions, we acquired the related gene symbols of these targets from the UniProt database (https://www.uniprot.org/) [17]. In this study, the compounds that cannot find corresponding gene symbols were excluded.

### 2.2. DN Targets Determination

For the purpose of obtaining a target list associate with DN, “diabetic nephropathy” was set as a keyword to search five disease database: Online Mendelian Inheritance in Man (OMIM) database (https://omim.org/) [18], GeneCards database (https://www.genecards.org/) [19], Therapeutic Target (TTD) database (http://db.idrblab.net/ttd/) [20], DisGeNET database (https://www.disgenet.org/home/) [21], and DrugBank database (https://go.drugbank.com/) [22]. After combining the acquired targets and removing the duplicate data, the candidate targets for the treatment of DN were obtained.

### 2.3. “Herbal-Compound-Target” Network Construction

After intersecting the acquired targets of formula and disease by using R language software, the common targets of LDP in treating DN were screened out. Then, we used Cytoscape 3.8.0 software to construct a “Herbal-compound-target” network [23]. The relationship between the active compounds and the anti-DN targets in LDP can be visualized easily on this network.

### 2.4. Protein-Protein Interaction (PPI) Network Construction

The STRING (https://string-db.org/, version 11.0) is a data platform that collects almost all known and predicted interactions between the expressed proteins [24]. We uploaded the acquired therapeutic targets to this web platform. The species parameter was set as “Homo sapiens,” and the confidence score was limited to “>0.7,” hide discrete targets, the PPI network was built, and the date of the network was exported.

### 2.5. Screening of Key Targets in the PPI Network

Cytoscape3.8.0 software was utilized to visualize and analyze the PPI network data. We then used the Cytoscape plugin CytoNCA [25] to screen critical targets in the PPI network. Betweenness centrality, closeness centrality, and degree centrality were chosen as the parameters to calculate topological features of the PPI network. Betweenness centrality was used to assess how much the shortest paths must pass through a given node [26]. Nodes with higher betweenness can be understood as connected to many nodes transmitting information [27]. Closeness centrality indicates the average distance between the nodes in the network [28]. Degree shows the number of edges to node [29]. Nodes with larger degree values are more crucial in the network [30]. The median of the three parameters was set as the thresholds for filtering central nodes to screen key targets.

### 2.6. Enrichment Analysis

Metascape (http://metascape.org) is an enrichment analysis platform updated monthly rather than longer to keep the data up to date [31]. To further illustrate the underlying mechanism of LDP for treating DN, we uploaded the acquired 131 common targets to Metascape online platform. Then, we performed GO and KEGG enrichment analyses on this platform. The “Min Overlap = 3,” “Min Enrichment = 1.5,” and “*P* value cutoff <0.01” were set as significant thresholds. Biological processes (BP), cellular components (CC), and molecular function (MF) are included in GO terms. After analysis, the top 10 terms of BP, CC, MF, and KEGG pathways were chosen to be visualized.

### 2.7. Molecular Docking

The binding capability of nine key targets and their corresponding active compounds were evaluated by molecular docking. To ensure the accuracy of the data, we used the UniProt database to find the UniProt ID of the key targets. According to the Uniport ID, the 3-dimensional (3D) protein structures related to the nine key targets were downloaded from the RCSB PDB online tools (http://www.rcsb.org/) [32]. The water molecules and small molecule ligands of the 3D protein structure were removed by PyMOL 2.4.0 software. The 2-dimensional (2D) structures of the compounds corresponding to the nine key targets were acquired from the PubChem database (https://pubchem.ncbi.nlm.nih.gov/) [33]. Then, the 2D structures of active compounds were converted into 3D structures by ChemBio3D 14.0 software. AutoDockTools 1.5.6 software was used to convert the “PDB” format file of 9 proteins and their corresponding active compounds into “pdbqt” format and define the location of their active pocket. At last, we performed molecular docking with AutoDock Vina 1.1.2 software.

## 3. Results

### 3.1. Active Compounds in LDP

A total of 41 active compounds of 6 herbs in LDP were acquired from the TCMSP database after screening (Table 1), including 2 compounds in Rehmanniae Radix Preparata (Shudihuang), 6 compounds in Cortex Moutan (Mudanpi), 12 compounds in Rhizoma Dioscoreae (Shanyao), 13 compounds in *Cornus officinalis* (Shanzhuyu), 7 compounds in *Alisma* (Zexie), and 6 compounds in *Poria cocos* (Fuling).

### 3.2. Target Prediction

**Figure 2:**
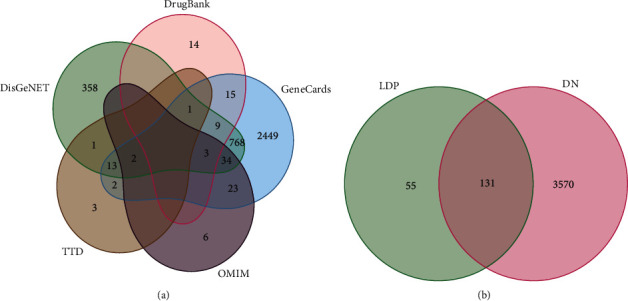
Venn diagram. (a) DN disease targets. (b) The intersection of LDP and DN disease targets.

In total, 186 corresponding targets of active compounds in LDP were screened out (Supplementary Table S1). The number of related targets in Rehmanniae Radix Preparata (Shudihuang), Cortex Moutan (Mudanpi), Rhizoma Dioscoreae (Shanyao), *Cornus officinalis* (Shanzhuyu), *Alisma* (Zexie), and *Poria cocos* (Fuling) was 27, 152, 62, 54, 5, and 19, respectively. As for the disease targets, 22 DN-related targets were acquired in TTD, 1189 in DisGeNET, 109 in DrugBank, 3319 in GeneCards, and 68 in OMIM. After removing duplicates, 3701 targets were identified (Figure 2(a) and Supplementary Table S2). Finally, 131 common targets of LDP and DN were obtained by intersecting the acquired targets (Figure 2(a), Supplementary Table S3).

### 3.3. “Herbal-Compound-Target” Network Construction

**Figure 3:**
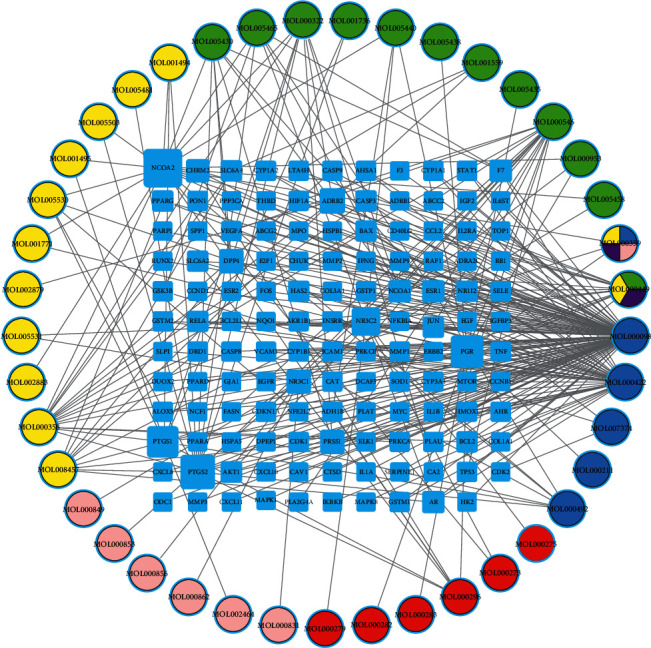
The “herbs-active compounds-disease targets” network. The circular nodes indicate active compounds, and the square nodes indicate the possible therapeutic targets in LDP. Different colors in the circular nodes represent that active compounds are included in different herbs. The yellow is Shanzhuyu, the green is Shanyao, the blue is Mudanpi, the red is Fuling, the pink is Zexie, and the purple is Shudihuang.

A “herbal-compound-target” network (Figure 3), including 172 nodes (41 active compound nodes of herb and 131 common target nodes) and 293 edges, was constructed. We obtained the “degree” parameter of the herbal-compound-target network by using an analysis network tool in Cytoscape software. After the network analysis, we found that the MOL000098 (quercetin, degree = 104), MOL000422 (kaempferol, degree = 42), MOL000358 (beta-sitosterol, degree = 16), MOL000546 (diosgenin, degree = 16), and MOL000449 (stigmasterol, degree = 13) were the top 5 compounds in the 41 active compounds. These five compounds may play the most significant role in treating DN. This result indicates that LDP played anti-DN roles mainly through these compounds. Overall, the relationships among herbs, active compounds, and disease targets can be observed through the network graph.

### 3.4. PPI Network Construction and Key Targets Analysis

**Figure 4:**
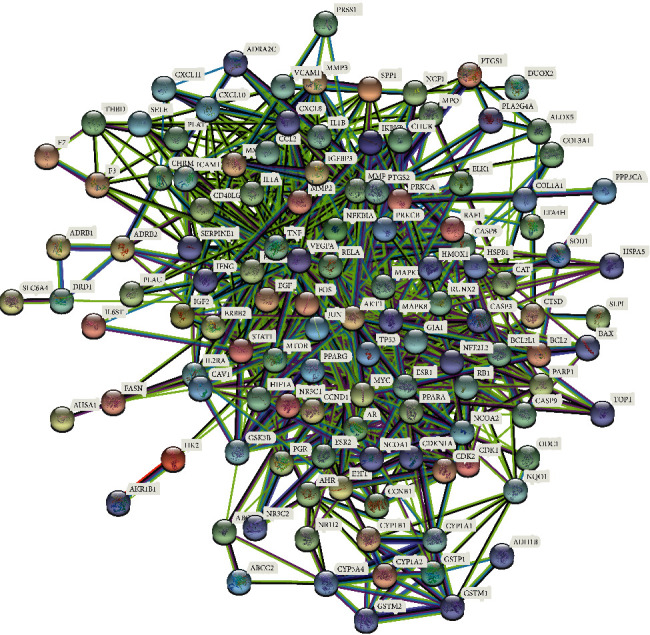
PPI network of LDP and DN common targets.

**Figure 5:**
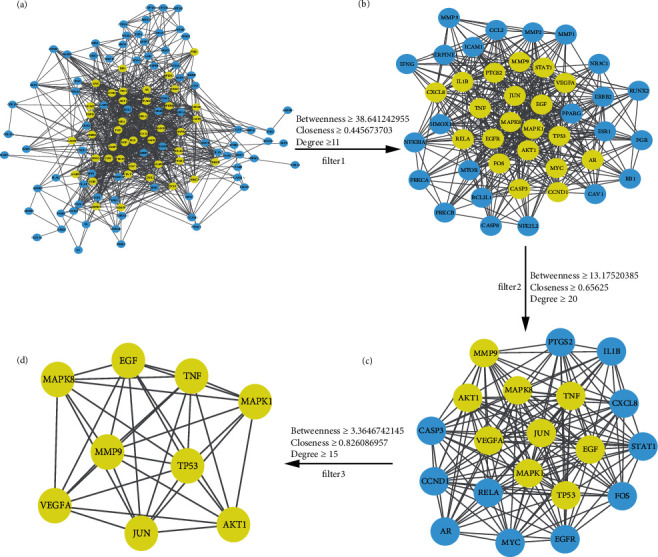
Screening of the key targets in the PPI network. (a) 122 nodes and 914 edges. The yellow genes are core genes, and they have a higher betweenness, closeness, and degree. (b) 43 nodes and 440 edges. (c) 20 nodes and 155 edges. (d) 9 nodes and 77 edges. The 9 targets in this network are considered the key targets in the whole PPI network, including JUN, MAPK8, AKT1, EGF, TP53, VEGFA, MMP9, MAPK1, and TNF.

For the purpose of further elucidating the mechanisms of LDP treatment of DN in vivo, we introduced 131 common targets into the STRING online service platform, and the date and figure of a PPI network were acquired (Figure 4). The PPI network data were visualized and analyzed via the “Analysis network” tool in Cytoscape 3.8.0 software. 122 nodes and 914 edges were included in the PPI network (Figure 5(a)). We tried to find the key targets by the topological features and set the median of betweenness, closeness, and degree centrality as the screening criteria. The thresholds of the first screening were betweenness ≥ 38.641242955, closeness ≥ 0.445673703, and degree ≥ 11. A new network including 43 nodes and 440 edges was acquired after the first screening (Figure 5(b)). Subsequently, betweenness ≥ 13.17520385, closeness ≥ 0.65625, and degree ≥ 20 were set as the thresholds of second screening. A center network that includes 20 nodes and 155 edges was constructed (Figure 5(c)). For the purpose of finding the most critical targets in the PPT network, betweenness ≥ 3.3646742145, closeness ≥ 0.826086957, and degree ≥ 15 were set as the last screening thresholds. Eventually, JUN, MAPK8, AKT1, EGF, TP53, VEGFA, MMP9, MAPK1, and TNF were screened as the key targets of the PPI network (Figure 5(d)).

### 3.5. Enrichment Analysis

**Figure 6:**
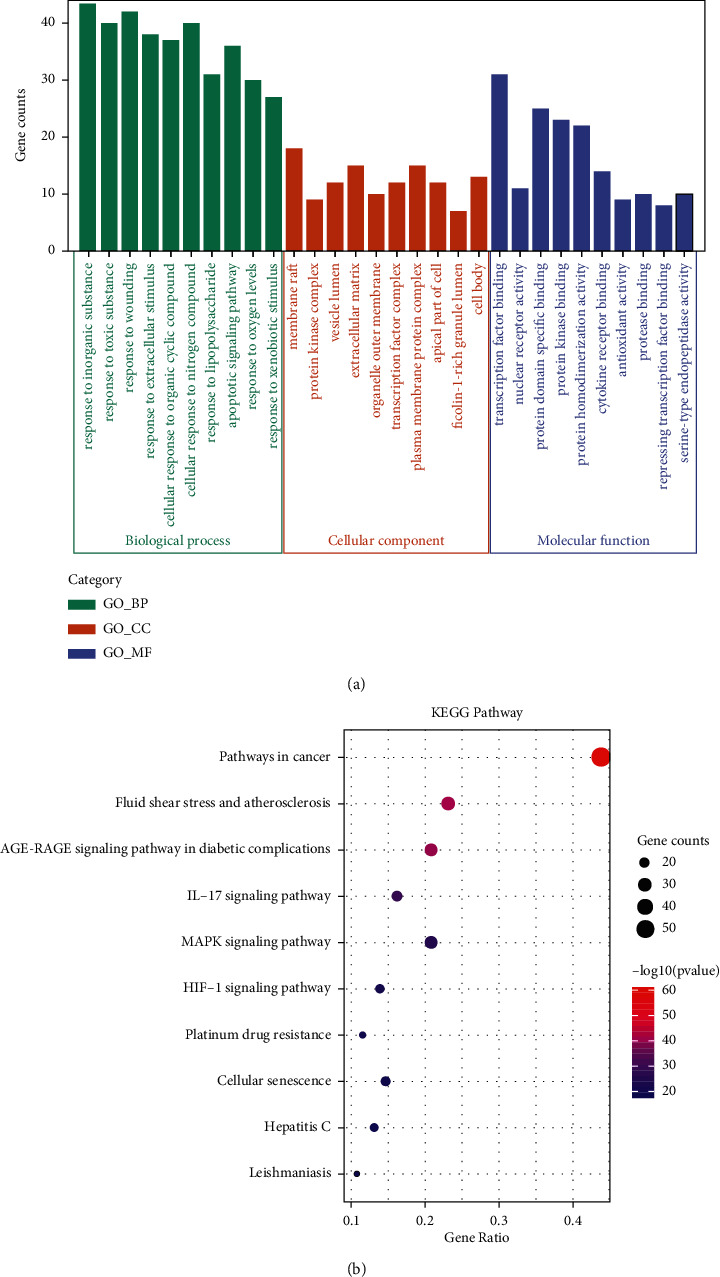
GO functional and KEGG pathway enrichment analyses. (a) The top 10 terms of BP, CC, and MF in GO functional enrichment analysis are shown. The height of the column in each part is closely related to the counts of potential targets. (b) The top 10 KEGG terms were closely associated with LDP in the treatment of DN. The redder the color, the larger the −log10 (*P* value). The bigger the size, the more potential targets are involved in the pathways.

GO and KEGG enrichment analyses of 131 common targets were performed on the Metascape data platform. This study found 2231 GO terms, including 2008 BP terms, 83 CC terms, and 143 MF terms. The top 10 most important terms of BP, CC, and MF are shown in Figure 6(a). BP terms mainly include response to the apoptotic signaling pathway, oxygen levels, lipopolysaccharide, wounding, organic cyclic compound, and nitrogen compound. CC terms were mainly enriched in membrane raft, protein kinase complex, vesicle lumen, transcription factor complex, plasma membrane protein complex, ficolin-1-rich granule lumen, organelle outer membrane, and cell body. MF terms mainly involved cytokine receptor binding, antioxidant activity, protein domain specific binding, transcription factor binding, nuclear receptor activity, protein homodimerization activity, repressing transcription factor binding, and protein kinase binding.

**Figure 7:**
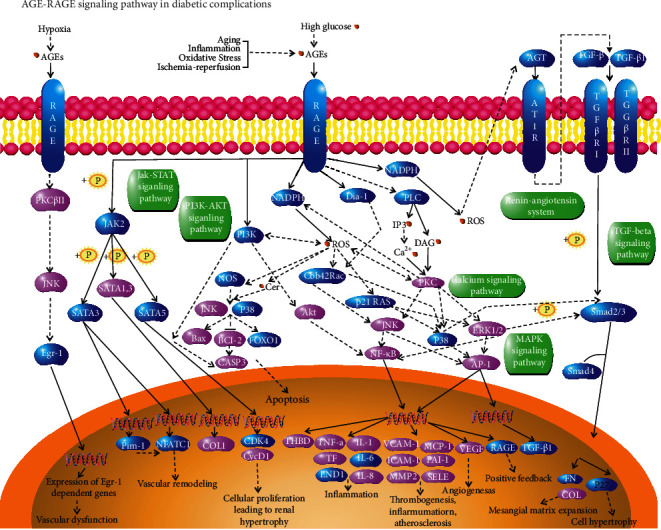
AGE-RAGE signaling pathway in diabetic complications. The pink nodes are the common targets of DN and LDP, and the blue nodes are others in the pathway.

339 pathways were acquired through the KEGG pathway enrichment analysis. The top 10 most important terms of the KEGG pathway enrichment analysis were chosen for the visual analysis (Figure 6(b)). The potential targets of LDP in treating DN were principally enriched in pathways in cancer, AGE-RAGE signaling pathway in diabetic complications, and L-17 signaling pathway. Besides, MAPK, HIF-1, platinum drug resistance, cellular senescence, hepatitis C, and leishmaniasis pathways were also included. The AGE-RAGE signaling pathway is closely connected with inflammatory response and diabetic complications and is selected to be further visualized as an example. The pink labeled nodes are the common targets of LDP and DN (Figure 7). It suggests that the AGE-RAGE signaling pathway is significant in LDP's anti-DN efficacy. More details of the GO functional and KEGG pathway analyses results are shown in additional file 1.

### 3.6. Molecular Docking

**Figure 8:**
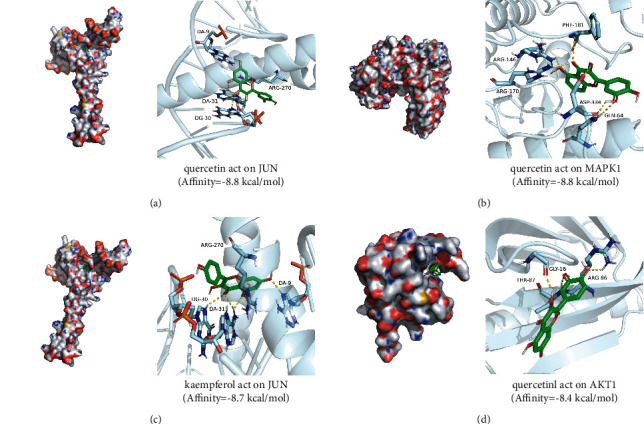
Four best molecular docking results. Molecular docking results between quercetin and JUN, quercetin and MAKP1, kaempferol and JUN, quercetin and AKT1, respectively.

For the purpose of validating the study results of the network analysis, the molecular docking between the nine key targets (JUN, MAPK8, AKT1, EGF, TP53, VEGFA, MMP9, MAPK1, and TNF) and their corresponding active compounds was performed. When the binding energy is <0 kJ mol, the small molecule ligand can spontaneously bind to the protein receptor. If the binding energy is <−5.0 kJ mol or lower, it indicates that the two have the better binding ability [34]. Through docking simulations, 16 pairs of docking results were yielded (Table 2). Their binding energy is all <−5 kJ mol, which means all of them can bind very well. The detailed information of the four best molecular docking targets and their corresponding active compounds is shown in Figure 8. Quercetin and JUN docking and quercetin and MAKP1 docking had the lowest binding energy (−8.8 kcal/mol), whereas the kaempferol and JUN docking (−8.7 kcal/mol) and quercetin and AKT1 docking (−8.4 kcal/mol) pairs with the second-and third-lowest binding energy. This molecular docking result indicates that their combination might have an essential role in treating DN with LDP.

## 4. Discussion

DN is a common diabetic complication that threatens the health and lives of diabetic patients. Unfortunately, most diabetic patients cannot obtain good treatment effects with routine therapies. Growing evidence has suggested that LDP may be a potential adjuvant or alternative medicine for DN [8–11]. However, the detailed mechanism of action remains obscure. As a relatively new approach in drug discovery, network pharmacology can illustrate the interaction between diseases, drugs, and targets [35, 36]. To some extent, the characteristics of network pharmacology coincide with the “multi-compounds, multi-targets, and multi-pathways” theory of traditional Chinese medicinal formula. Therefore, we explored the mechanism of action of the Chinese medicine formula LDP as an adjuvant treatment of DN through a network pharmacology approach. Moreover, the inner links between LDP and DN were further verified via molecular docking. The present study improves the understanding of the molecular mechanism of LDN in treating DN, which is of great importance for further basic research and clinical application.

Based on the results of the network analysis, quercetin, kaempferol, beta-sitosterol, diosgenin, and stigmasterol can be defined as the crucial compounds of LDP in treating DN. It is reported that quercetin could inhibit inflammatory cell infiltration, alleviate renal oxidative stress injury, relieve the pathological damage of the kidney, and improve renal function in DN [37]. Kaempferol has anti-inflammatory, antioxidant, and antifibrotic properties in DN [38, 39]. Beta-sitosterol has been identified as a potential herbal nutraceutical for DN because it has anti-inflammatory, lipid-lowering, antioxidant, and antidiabetic activities [40]. Diosgenin plays a protective role in DN through lowering oxidative stress and inflammation [41]. Stigmasterol has the function of regulating the glucose metabolism [42]. These main LDP compounds collectively exert anti-inflammation, antioxidant, antifibrotic, antihyperglycemic, and antihyperlipidemic effects which can form a pharmacological basis for the anti-DN function of LDP.

Through the PPI network analysis, JUN, MAPK8, AKT1, EGF, TP53, VEGFA, MMP9, MAPK1, and TNF were the key targets of LDP in treating DN. These targets are mainly connected with inflammation, vascular permeability, and oxidative stress. In some ways, this is consistent with the disease characteristics and pathogenesis of DN. To further reveal LDP's possible anti-DN molecular mechanism, we conducted molecular docking of 9 key targets with their corresponding active compounds. Study results have shown that the nine key targets have an excellent ability to bind their related active compounds in LDP. Among them, JUN, MAKP1, and AKT1 had a more stable binding ability than others. A recent study showed that c-Jun could be progressively elevated, and it could activate the expression of TGF*β*1 via ross-activation and autoregulation during renal fibrosis in DN [43]. MAPK1 can increase many inflammatory and adhesion factors in glomerular cells and exacerbate the damage in the pathological state of DN [44]. AKT1 is closely associated with the immune regulation and inflammation reaction of DN. It plays a vital role in basement membrane thickening, mesangial proliferation, and podocyte injury [45].

The GO functional enrichment analysis of the 131 common targets was carried out (Figure 6(a)). The 10 most meaningful enriched BP terms were principally associated with apoptosis, response to oxygen levels, and response to lipopolysaccharide. Related research demonstrated that the initiation and progression of DN are closely associated with apoptosis, oxidative stress, and lipopolysaccharide level [46, 47]. MF terms mainly included transcription factor binding, protein homodimerization activity, cytokine receptor binding, and antioxidant activity. The targets primarily enriched in the above MF terms were JUN, TNF, VEGFA, SOD1, DPP4, and AKT1. They are principally included in inflammatory regulation, immune response, and oxidative stress. Inflammation and immune response play essential roles in the progression of DN [48]. CC terms were mainly enriched in membrane raft, protein kinase complex, extracellular matrix, transcription factor complex, and vesicle lumen. The key targets, such as TNF, MAPK1, and VEGFA, were included in these terms. These finds indicated that DN is very complex, and the LDP could be used to treat DN by interfering with various molecular functions and cellular components.

Associated with the GO enrichment analysis, we found out that the main pathways of LDP on DN might be the AGE-RAGE signaling pathway and IL-17 signaling pathway based on the enrichment results of KEGG. As we all know, the AGE-RAGE signaling pathway is of great significance to diabetic complications. The upregulation of AGEs levels and RAGE expression can aggravate the progression of DN [49]. When the kidney is subjected to long-term stimulation of glycosylation of reducing sugars, the AGEs are gradually accumulated and increase the risk of extracellular matrix migration, renal tubular dysfunction, and glomerular proliferative lesion. Furthermore, AGEs can bind to RAGE receptors to cause chronic inflammation reaction, oxidative stress, kidney tissue damage, and the loss of kidney function [50]. Sharma et al. have reported that AGE-RAGE interaction promotes DN's progression because of the release of fibronectin, TGF-*β*, and inflammatory cytokines [51]. If the IL-17 pathway is activated in many kidney diseases, it can promote inflammatory cytokines [52]. Inflammatory cytokines can cause glomerulosclerosis and kidney tissue damage in DN via inflammatory response [53]. Mohamed et al. have shown that low-dose recombinant IL-17 might prevent and reverse DN [54].

To some extent, our results are supported by the previous studies, which made them more reliable. However, there are also some limitations in this study. For example, it is difficult to ensure that the drug's active ingredients are identical to those absorbed into the patient's bloodstream; We are still vague about the interaction effects of different nodes in the network analysis. The functions and pathways highly researched may cause departures from expected results. Thus, further experimental and clinical studies are warranted to verify our theoretical prediction.

## 5. Conclusion

This research proved that the therapeutic mechanism of LDP on DN might be realized by multitargets, multiactive compounds, and multipathways. We found that quercetin, kaempferol, beta-sitosterol, diosgenin, and stigmasterol can be defined as five crucial compounds. JUN, MAPK8, AKT1, EGF, TP53, VEGFA, MMP9, MAPK1, and TNF may be the nine most important therapeutic targets. AGE-RAGE and IL-17 are two key signal pathways of LDP for the treatment of DN. The potential pharmacological mechanism mainly associates with inflammation reaction, oxidative stress, immune regulation, and cell apoptosis. Also, some active compounds, target genes, and pathways in our study have few reports, which may be the clues for further research in the mechanism of LDP in treating DN. In summary, our study provides valuable evidence for further basic research and clinical applications.

## Figures and Tables

**Table 1:** 41 active compounds information in LDP.

Mol ID	Compound	Drug	OB (%)	DL
MOL000273	(2R)-2-[(3S,5R,10S,13R,14R,16R,17R)-3,16-Dihydroxy-4,4,10,13,14-pentamethyl-2,3,5,6,12,15,16,17-octahydro-1H-cyclopenta[a]phenanthren-17-yl]-6-methylhept-5-enoic acid	Fuling	30.93	0.81
MOL000275	Trametenolic acid	Fuling	38.71	0.8
MOL000283	Ergosterol peroxide	Fuling	40.36	0.81
MOL000279	Cerevisterol	Fuling	37.96	0.77
MOL000296	Hederagenin	Fuling	36.91	0.75
MOL000282	Ergosta-7,22E-dien-3beta-ol	Fuling	43.51	0.72
MOL000098	Quercetin	Mudanpi	46.43	0.28
MOL000211	Mairin	Mudanpi	55.38	0.78
MOL000422	Kaempferol	Mudanpi	41.88	0.24
MOL000492	(+)-Catechin	Mudanpi	54.83	0.24
MOL007374	5-[[5-(4-Methoxyphenyl)-2-furyl]methylene]barbituric- acid	Mudanpi	43.44	0.3
MOL000322	Kadsurenone	Shanyao	54.72	0.38
MOL000546	Diosgenin	Shanyao	80.88	0.81
MOL000953	CLR	Shanyao	37.87	0.68
MOL001559	Piperlonguminine	Shanyao	30.71	0.18
MOL001736	(−)-Taxifolin	Shanyao	60.51	0.27
MOL005430	Hancinone C	Shanyao	59.05	0.39
MOL005435	24-Methylcholest-5-enyl-3belta-O-glucopyranoside_qt	Shanyao	37.58	0.72
MOL005438	Campesterol	Shanyao	37.58	0.71
MOL005440	Isofucosterol	Shanyao	43.78	0.76
MOL005458	Dioscoreside C_qt	Shanyao	36.38	0.87
MOL005465	AIDS180907	Shanyao	45.33	0.77
MOL000449	Stigmasterol	Shanyao, Shanzhuyu, Shudihuang	43.83	0.76
MOL005531	Telocinobufagin	Shanzhuyu	69.99	0.79
MOL000358	Beta-sitosterol	Shanzhuyu	36.91	0.75
MOL005481	2,6,10,14,18-Pentamethylicosa-2,6,10,14,18-pentaene	Shanzhuyu	33.4	0.24
MOL001495	Ethyl linolenate	Shanzhuyu	46.1	0.2
MOL005503	Cornudentanone	Shanzhuyu	39.66	0.33
MOL002879	Diop	Shanzhuyu	43.59	0.39
MOL002883	Ethyl oleate (NF)	Shanzhuyu	32.4	0.19
MOL001771	Poriferast-5-en-3beta-ol	Shanzhuyu	36.91	0.75
MOL005530	Hydroxygenkwanin	Shanzhuyu	36.47	0.27
MOL001494	Mandenol	Shanzhuyu	42	0.19
MOL008457	Tetrahydroalstonine	Shanzhuyu	32.42	0.81
MOL000831	Alisol B monoacetate	Zexie	35.58	0.81
MOL000849	16*β*-Methoxyalisol B monoacetate	Zexie	32.43	0.77
MOL000853	Alisol B	Zexie	36.76	0.82
MOL000856	Alisol C monoacetate	Zexie	33.06	0.83
MOL000862	[(1S,3R)-1-[(2R)-3,3-Dimethyloxiran-2-yl]-3-[(5R,8S,9S,10S,11S,14R)-11-hydroxy-4,4,8,10,14-pentamethyl-3-oxo-1,2,5,6,7,9,11,12,15,16-decahydrocyclopenta[a]phenanthren-17-yl]butyl] acetate	Zexie	35.58	0.81
MOL002464	1-Monolinolein	Zexie	37.18	0.3
MOL000359	Sitosterol	Zexie, Mudanpi, Shanzhuyu, Shudihuang	36.91	0.75

**Table 2:** Molecular docking results of nine key genes with their corresponding compounds in LDP.

PDB ID	Key targets	Compounds	Binding energy (kcal/mol)
5T01	JUN	Quercetin	−8.8
3ZU7	MAPK1	Quercetin	−8.8
5T01	JUN	Kaempferol	−8.7
2UVM	AKT1	Quercetin	−8.4
2UVM	AKT1	Kaempferol	−8.1
4QAF	VEGFA	Diosgenin	−8.1
4H82	MMP9	Quercetin	−8.1
2TNF	TNF	Quercetin	−8.1
2NO3	MAPK8	Kaempferol	−8.0
4QAF	VEGFA	Quercetin	−7.9
2TNF	TNF	Kaempferol	−7.9
2UVM	AKT1	Diosgenin	−7.7
5T01	JUN	Beta-sitosterol	−7.7
2RUK	TP53	Diosgenin	−7.6
2RUK	TP53	Quercetin	−6.9
2KV4	EGF	Quercetin	−6.9

## Data Availability

The relevant data and code used to support the findings of this study are available from the corresponding author upon request for two years after publication.

## Supplementary Materials

Supplementary Materials Table S1: 186 putative targets of LDP. Table S2: 3701 disease targets of DN. Table S3: 131 common targets of LDP and DN. Table S4: GO functional enrichment analysis. Table S5: KEGG pathway enrichment analysis. Figures S1–S4: heatmap of the enrichment analysis results downloaded from Metascape online platform.
