# Supplementary material for: Exploring the Molecular Mechanism of Liuwei Dihuang Pills for Treating Diabetic Nephropathy by Combined Network Pharmacology and Molecular Docking
Source: Evid Based Complement Alternat Med. 2021 Sep 13;2021:7262208. doi: 10.1155/2021/7262208 (PMC8452392; doi:10.1155/2021/7262208)
Supplement: Supplementary Materials — Table S1: 186 putative targets of LDP. Table S2: 3701 disease targets of DN. Table S3: 131 common targets of LDP and DN. Table S4: GO functional enrichment analysis. Table S5: KEGG pathway enrichment analysis. Figures S1–S4: heatmap of the enrichment analysis results downloaded from Metascape online platform. [file 7262208.f1.zip › 7262208.f1/Table S1 186 putative targets of LDP.docx]

Table S1: 186 putative targets of LDP

| **Unitprot ID** | **Protein names** | **Gene symbol** | **Entry name** |
| --- | --- | --- | --- |
| P15692 | Vascular endothelial growth factor A (VEGF-A) (Vascular permeability factor) (VPF) | VEGFA | VEGFA_HUMAN |
| P19320 | Vascular cell adhesion protein 1 (V-CAM 1) (VCAM-1) (INCAM-100) (CD antigen CD106) | VCAM1 | VCAM1_HUMAN |
| P04637 | Cellular tumor antigen p53 (Antigen NY-CO-13) (Phosphoprotein p53) (Tumor suppressor p53) | TP53 | P53_HUMAN |
| P11388 | DNA topoisomerase 2-alpha (EC 5.6.2.2) (DNA topoisomerase II, alpha isozyme) | TOP2A | TOP2A_HUMAN |
| P11387 | DNA topoisomerase 1 (EC 5.6.2.1) (DNA topoisomerase I) | TOP1 | TOP1_HUMAN |
| P01375 | Tumor necrosis factor (Cachectin) (TNF-alpha) (Tumor necrosis factor ligand superfamily member 2) (TNF-a) [Cleaved into: Tumor necrosis factor, membrane form (N-terminal fragment) (NTF); Intracellular domain 1 (ICD1); Intracellular domain 2 (ICD2); C-domain 1; C-domain 2; Tumor necrosis factor, soluble form] | TNF | TNFA_HUMAN |
| P07204 | Thrombomodulin (TM) (Fetomodulin) (CD antigen CD141) | THBD | TRBM_HUMAN |
| P49888 | Sulfotransferase 1E1 (ST1E1) (EC 2.8.2.4) (EST-1) (Estrogen sulfotransferase) (Sulfotransferase, estrogen-preferring) | SULT1E1 | ST1E1_HUMAN |
| P42224 | Signal transducer and activator of transcription 1-alpha/beta (Transcription factor ISGF-3 components p91/p84) | STAT1 | STAT1_HUMAN |
| P10451 | Osteopontin (Bone sialoprotein 1) (Nephropontin) (Secreted phosphoprotein 1) (SPP-1) (Urinary stone protein) (Uropontin) | SPP1 | OSTP_HUMAN |
| P00441 | Superoxide dismutase [Cu-Zn] (EC 1.15.1.1) (Superoxide dismutase 1) (hSod1) | SOD1 | SODC_HUMAN |
| P03973 | Antileukoproteinase (ALP) (BLPI) (HUSI-1) (Mucus proteinase inhibitor) (MPI) (Protease inhibitor WAP4) (Secretory leukocyte protease inhibitor) (Seminal proteinase inhibitor) (WAP four-disulfide core domain protein 4) | SLPI | SLPI_HUMAN |
| P31645 | Sodium-dependent serotonin transporter (SERT) (5HT transporter) (5HTT) (Solute carrier family 6 member 4) | SLC6A4 | SC6A4_HUMAN |
| Q01959 | Sodium-dependent dopamine transporter (DA transporter) (DAT) (Solute carrier family 6 member 3) | SLC6A3 | SC6A3_HUMAN |
| P23975 | Sodium-dependent noradrenaline transporter (Norepinephrine transporter) (NET) (Solute carrier family 6 member 2) | SLC6A2 | SC6A2_HUMAN |
| P05121 | Plasminogen activator inhibitor 1 (PAI) (PAI-1) (Endothelial plasminogen activator inhibitor) (Serpin E1) | SERPINE1 | PAI1_HUMAN |
| P16581 | E-selectin (CD62 antigen-like family member E) (Endothelial leukocyte adhesion molecule 1) (ELAM-1) (Leukocyte-endothelial cell adhesion molecule 2) (LECAM2) (CD antigen CD62E) | SELE | LYAM2_HUMAN |
| Q14524 | Sodium channel protein type 5 subunit alpha (Sodium channel protein cardiac muscle subunit alpha) (Sodium channel protein type V subunit alpha) (Voltage-gated sodium channel subunit alpha Nav1.5) (hH1) | SCN5A | SCN5A_HUMAN |
| P19793 | Retinoic acid receptor RXR-alpha (Nuclear receptor subfamily 2 group B member 1) (Retinoid X receptor alpha) | RXRA | RXRA_HUMAN |
| Q13950 | Runt-related transcription factor 2 (Acute myeloid leukemia 3 protein) (Core-binding factor subunit alpha-1) (CBF-alpha-1) (Oncogene AML-3) (Osteoblast-specific transcription factor 2) (OSF-2) (Polyomavirus enhancer-binding protein 2 alpha A subunit) (PEA2-alpha A) (PEBP2-alpha A) (SL3-3 enhancer factor 1 alpha A subunit) (SL3/AKV core-binding factor alpha A subunit) | RUNX2 | RUNX2_HUMAN |
| Q06455 | Protein CBFA2T1 (Cyclin-D-related protein) (Eight twenty one protein) (Protein ETO) (Protein MTG8) (Zinc finger MYND domain-containing protein 2) | RUNX1T1 | MTG8_HUMAN |
| Q04206 | Transcription factor p65 (Nuclear factor NF-kappa-B p65 subunit) (Nuclear factor of kappa light polypeptide gene enhancer in B-cells 3) | RELA | TF65_HUMAN |
| P06400 | Retinoblastoma-associated protein (p105-Rb) (p110-RB1) (pRb) (Rb) (pp110) | RB1 | RB_HUMAN |
| Q9NS23 | Ras association domain-containing protein 1 | RASSF1 | RASF1_HUMAN |
| P20936 | Ras GTPase-activating protein 1 (GAP) (GTPase-activating protein) (RasGAP) (Ras p21 protein activator) (p120GAP) | RASA1 | RASA1_HUMAN |
| P04049 | RAF proto-oncogene serine/threonine-protein kinase (EC 2.7.11.1) (Proto-oncogene c-RAF) (cRaf) (Raf-1) | RAF1 | RAF1_HUMAN |
| P35354 | Prostaglandin G/H synthase 2 (EC 1.14.99.1) (Cyclooxygenase-2) (COX-2) (PHS II) (Prostaglandin H2 synthase 2) (PGH synthase 2) (PGHS-2) (Prostaglandin-endoperoxide synthase 2) | PTGS2 | PGH2_HUMAN |
| P23219 | Prostaglandin G/H synthase 1 (EC 1.14.99.1) (Cyclooxygenase-1) (COX-1) (Prostaglandin H2 synthase 1) (PGH synthase 1) (PGHS-1) (PHS 1) (Prostaglandin-endoperoxide synthase 1) | PTGS1 | PGH1_HUMAN |
| P43115 | Prostaglandin E2 receptor EP3 subtype (PGE receptor EP3 subtype) (PGE2 receptor EP3 subtype) (PGE2-R) (Prostanoid EP3 receptor) | PTGER3 | PE2R3_HUMAN |
| O43242 | 26S proteasome non-ATPase regulatory subunit 3 (26S proteasome regulatory subunit RPN3) (26S proteasome regulatory subunit S3) (Proteasome subunit p58) | PSMD3 | PSMD3_HUMAN |
| P07477 | Trypsin-1 (EC 3.4.21.4) (Beta-trypsin) (Cationic trypsinogen) (Serine protease 1) (Trypsin I) [Cleaved into: Alpha-trypsin chain 1; Alpha-trypsin chain 2] | PRSS1 | TRY1_HUMAN |
| P05771 | Protein kinase C beta type (PKC-B) (PKC-beta) (EC 2.7.11.13) | PRKCB | KPCB_HUMAN |
| P17252 | Protein kinase C alpha type (PKC-A) (PKC-alpha) (EC 2.7.11.13) | PRKCA | KPCA_HUMAN |
| Q08209 | Serine/threonine-protein phosphatase 2B catalytic subunit alpha isoform (EC 3.1.3.16) (CAM-PRP catalytic subunit) (Calmodulin-dependent calcineurin A subunit alpha isoform) | PPP3CA | PP2BA_HUMAN |
| P37231 | Peroxisome proliferator-activated receptor gamma (PPAR-gamma) (Nuclear receptor subfamily 1 group C member 3) | PPARG | PPARG_HUMAN |
| Q03181 | Peroxisome proliferator-activated receptor delta (PPAR-delta) (NUCI) (Nuclear hormone receptor 1) (NUC1) (Nuclear receptor subfamily 1 group C member 2) (Peroxisome proliferator-activated receptor beta) (PPAR-beta) | PPARD | PPARD_HUMAN |
| Q07869 | Peroxisome proliferator-activated receptor alpha (PPAR-alpha) (Nuclear receptor subfamily 1 group C member 1) | PPARA | PPARA_HUMAN |
| P16435 | NADPH--cytochrome P450 reductase (CPR) (P450R) (EC 1.6.2.4) | POR | NCPR_HUMAN |
| P27169 | Serum paraoxonase/arylesterase 1 (PON 1) (EC 3.1.1.2) (EC 3.1.1.81) (EC 3.1.8.1) (Aromatic esterase 1) (A-esterase 1) (K-45) (Serum aryldialkylphosphatase 1) | PON1 | PON1_HUMAN |
| P00749 | Urokinase-type plasminogen activator (U-plasminogen activator) (uPA) (EC 3.4.21.73) [Cleaved into: Urokinase-type plasminogen activator long chain A; Urokinase-type plasminogen activator short chain A; Urokinase-type plasminogen activator chain B] | PLAU | UROK_HUMAN |
| P00750 | Tissue-type plasminogen activator (t-PA) (t-plasminogen activator) (tPA) (EC 3.4.21.68) (Alteplase) (Reteplase) [Cleaved into: Tissue-type plasminogen activator chain A; Tissue-type plasminogen activator chain B] | PLAT | TPA_HUMAN |
| P47712 | Cytosolic phospholipase A2 (cPLA2) (Phospholipase A2 group IVA) [Includes: Phospholipase A2 (EC 3.1.1.4) (Phosphatidylcholine 2-acylhydrolase); Lysophospholipase (EC 3.1.1.5)] | PLA2G4A | PA24A_HUMAN |
| P06401 | Progesterone receptor (PR) (Nuclear receptor subfamily 3 group C member 3) | PGR | PRGR_HUMAN |
| Q15113 | Procollagen C-endopeptidase enhancer 1 (Procollagen COOH-terminal proteinase enhancer 1) (PCPE-1) (Procollagen C-proteinase enhancer 1) (Type 1 procollagen C-proteinase enhancer protein) (Type I procollagen COOH-terminal proteinase enhancer) | PCOLCE | PCOC1_HUMAN |
| P09874 | Poly [ADP-ribose] polymerase 1 (PARP-1) (EC 2.4.2.30) (ADP-ribosyltransferase diphtheria toxin-like 1) (ARTD1) (DNA ADP-ribosyltransferase PARP1) (EC 2.4.2.-) (NAD(+) ADP-ribosyltransferase 1) (ADPRT 1) (Poly[ADP-ribose] synthase 1) (Protein poly-ADP-ribosyltransferase PARP1) (EC 2.4.2.-) | PARP1 | PARP1_HUMAN |
| P35372 | Mu-type opioid receptor (M-OR-1) (MOR-1) (Mu opiate receptor) (Mu opioid receptor) (MOP) (hMOP) | OPRM1 | OPRM_HUMAN |
| P41143 | Delta-type opioid receptor (D-OR-1) (DOR-1) | OPRD1 | OPRD_HUMAN |
| P11926 | Ornithine decarboxylase (ODC) (EC 4.1.1.17) | ODC1 | DCOR_HUMAN |
| P08235 | Mineralocorticoid receptor (MR) (Nuclear receptor subfamily 3 group C member 2) | NR3C2 | MCR_HUMAN |
| P04150 | Glucocorticoid receptor (GR) (Nuclear receptor subfamily 3 group C member 1) | NR3C1 | GCR_HUMAN |
| Q14994 | Nuclear receptor subfamily 1 group I member 3 (Constitutive activator of retinoid response) (Constitutive active response) (Constitutive androstane receptor) (CAR) (Orphan nuclear receptor MB67) | NR1I3 | NR1I3_HUMAN |
| O75469 | Nuclear receptor subfamily 1 group I member 2 (Orphan nuclear receptor PAR1) (Orphan nuclear receptor PXR) (Pregnane X receptor) (Steroid and xenobiotic receptor) (SXR) | NR1I2 | NR1I2_HUMAN |
| P15559 | NAD(P)H dehydrogenase [quinone] 1 (EC 1.6.5.2) (Azoreductase) (DT-diaphorase) (DTD) (Menadione reductase) (NAD(P)H:quinone oxidoreductase 1) (Phylloquinone reductase) (Quinone reductase 1) (QR1) | NQO1 | NQO1_HUMAN |
| P55786 | Puromycin-sensitive aminopeptidase (PSA) (EC 3.4.11.14) (Cytosol alanyl aminopeptidase) (AAP-S) | NPEPPS | PSA_HUMAN |
| P25963 | NF-kappa-B inhibitor alpha (I-kappa-B-alpha) (IkB-alpha) (IkappaBalpha) (Major histocompatibility complex enhancer-binding protein MAD3) | NFKBIA | IKBA_HUMAN |
| Q16236 | Nuclear factor erythroid 2-related factor 2 (NF-E2-related factor 2) (NFE2-related factor 2) (Nrf-2) (HEBP1) (Nuclear factor, erythroid derived 2, like 2) | NFE2L2 | NF2L2_HUMAN |
| Q15596 | Nuclear receptor coactivator 2 (NCoA-2) (Class E basic helix-loop-helix protein 75) (bHLHe75) (Transcriptional intermediary factor 2) (hTIF2) | NCOA2 | NCOA2_HUMAN |
| Q15788 | Nuclear receptor coactivator 1 (NCoA-1) (EC 2.3.1.48) (Class E basic helix-loop-helix protein 74) (bHLHe74) (Protein Hin-2) (RIP160) (Renal carcinoma antigen NY-REN-52) (Steroid receptor coactivator 1) (SRC-1) | NCOA1 | NCOA1_HUMAN |
| P14598 | Neutrophil cytosol factor 1 (NCF-1) (47 kDa autosomal chronic granulomatous disease protein) (47 kDa neutrophil oxidase factor) (NCF-47K) (Neutrophil NADPH oxidase factor 1) (Nox organizer 2) (Nox-organizing protein 2) (SH3 and PX domain-containing protein 1A) (p47-phox) | NCF1 | NCF1_HUMAN |
| P01106 | Myc proto-oncogene protein (Class E basic helix-loop-helix protein 39) (bHLHe39) (Proto-oncogene c-Myc) (Transcription factor p64) | MYC | MYC_HUMAN |
| P42345 | Serine/threonine-protein kinase mTOR (EC 2.7.11.1) (FK506-binding protein 12-rapamycin complex-associated protein 1) (FKBP12-rapamycin complex-associated protein) (Mammalian target of rapamycin) (mTOR) (Mechanistic target of rapamycin) (Rapamycin and FKBP12 target 1) (Rapamycin target protein 1) | MTOR | MTOR_HUMAN |
| P05164 | Myeloperoxidase (MPO) (EC 1.11.2.2) [Cleaved into: Myeloperoxidase; 89 kDa myeloperoxidase; 84 kDa myeloperoxidase; Myeloperoxidase light chain; Myeloperoxidase heavy chain] | MPO | PERM_HUMAN |
| P14780 | Matrix metalloproteinase-9 (MMP-9) (EC 3.4.24.35) (92 kDa gelatinase) (92 kDa type IV collagenase) (Gelatinase B) (GELB) [Cleaved into: 67 kDa matrix metalloproteinase-9; 82 kDa matrix metalloproteinase-9] | MMP9 | MMP9_HUMAN |
| P08254 | Stromelysin-1 (SL-1) (EC 3.4.24.17) (Matrix metalloproteinase-3) (MMP-3) (Transin-1) | MMP3 | MMP3_HUMAN |
| P08253 | 72 kDa type IV collagenase (EC 3.4.24.24) (72 kDa gelatinase) (Gelatinase A) (Matrix metalloproteinase-2) (MMP-2) (TBE-1) [Cleaved into: PEX] | MMP2 | MMP2_HUMAN |
| P03956 | Interstitial collagenase (EC 3.4.24.7) (Fibroblast collagenase) (Matrix metalloproteinase-1) (MMP-1) [Cleaved into: 22 kDa interstitial collagenase; 27 kDa interstitial collagenase] | MMP1 | MMP1_HUMAN |
| P45983 | Mitogen-activated protein kinase 8 (MAP kinase 8) (MAPK 8) (EC 2.7.11.24) (JNK-46) (Stress-activated protein kinase 1c) (SAPK1c) (Stress-activated protein kinase JNK1) (c-Jun N-terminal kinase 1) | MAPK8 | MK08_HUMAN |
| P28482 | Mitogen-activated protein kinase 1 (MAP kinase 1) (MAPK 1) (EC 2.7.11.24) (ERT1) (Extracellular signal-regulated kinase 2) (ERK-2) (MAP kinase isoform p42) (p42-MAPK) (Mitogen-activated protein kinase 2) (MAP kinase 2) (MAPK 2) | MAPK1 | MK01_HUMAN |
| P11137 | Microtubule-associated protein 2 (MAP-2) | MAP2 | MTAP2_HUMAN |
| P27338 | Amine oxidase [flavin-containing] B (EC 1.4.3.4) (Monoamine oxidase type B) (MAO-B) | MAOB | AOFB_HUMAN |
| P21397 | Amine oxidase [flavin-containing] A (EC 1.4.3.4) (Monoamine oxidase type A) (MAO-A) | MAOA | AOFA_HUMAN |
| O75951 | Lysozyme-like protein 6 (EC 3.2.1.17) | LYZL6 | LYZL6_HUMAN |
| P09960 | Leukotriene A-4 hydrolase (LTA-4 hydrolase) (EC 3.3.2.6) (Leukotriene A(4) hydrolase) (Tripeptide aminopeptidase LTA4H) (EC 3.4.11.4) | LTA4H | LKHA4_HUMAN |
| Q12809 | Potassium voltage-gated channel subfamily H member 2 (Eag homolog) (Ether-a-go-go-related gene potassium channel 1) (ERG-1) (Eag-related protein 1) (Ether-a-go-go-related protein 1) (H-ERG) (hERG-1) (hERG1) (Voltage-gated potassium channel subunit Kv11.1) | KCNH2 | KCNH2_HUMAN |
| P05412 | Transcription factor AP-1 (Activator protein 1) (AP1) (Proto-oncogene c-Jun) (V-jun avian sarcoma virus 17 oncogene homolog) (p39) | JUN | JUN_HUMAN |
| P10914 | Interferon regulatory factor 1 (IRF-1) | IRF1 | IRF1_HUMAN |
| P14616 | Insulin receptor-related protein (IRR) (EC 2.7.10.1) (IR-related receptor) [Cleaved into: Insulin receptor-related protein alpha chain; Insulin receptor-related protein beta chain] | INSRR | INSRR_HUMAN |
| P40189 | Interleukin-6 receptor subunit beta (IL-6 receptor subunit beta) (IL-6R subunit beta) (IL-6R-beta) (IL-6RB) (CDw130) (Interleukin-6 signal transducer) (Membrane glycoprotein 130) (gp130) (Oncostatin-M receptor subunit alpha) (CD antigen CD130) | IL6ST | IL6RB_HUMAN |
| P01589 | Interleukin-2 receptor subunit alpha (IL-2 receptor subunit alpha) (IL-2-RA) (IL-2R subunit alpha) (IL2-RA) (TAC antigen) (p55) (CD antigen CD25) | IL2RA | IL2RA_HUMAN |
| P01584 | Interleukin-1 beta (IL-1 beta) (Catabolin) | IL1B | IL1B_HUMAN |
| P01583 | Interleukin-1 alpha (IL-1 alpha) (Hematopoietin-1) | IL1A | IL1A_HUMAN |
| Q08334 | Interleukin-10 receptor subunit beta (IL-10 receptor subunit beta) (IL-10R subunit beta) (IL-10RB) (Cytokine receptor class-II member 4) (Cytokine receptor family 2 member 4) (CRF2-4) (Interleukin-10 receptor subunit 2) (IL-10R subunit 2) (IL-10R2) (CD antigen CDw210b) | IL10RB | I10R2_HUMAN |
| O14920 | Inhibitor of nuclear factor kappa-B kinase subunit beta (I-kappa-B-kinase beta) (IKK-B) (IKK-beta) (IkBKB) (EC 2.7.11.10) (I-kappa-B kinase 2) (IKK2) (Nuclear factor NF-kappa-B inhibitor kinase beta) (NFKBIKB) (Serine/threonine protein kinase IKBKB) (EC 2.7.11.1) | IKBKB | IKKB_HUMAN |
| P01857 | Immunoglobulin heavy constant gamma 1 (Ig gamma-1 chain C region) (Ig gamma-1 chain C region EU) (Ig gamma-1 chain C region KOL) (Ig gamma-1 chain C region NIE) | IGHG1 | IGHG1_HUMAN |
| P17936 | Insulin-like growth factor-binding protein 3 (IBP-3) (IGF-binding protein 3) (IGFBP-3) | IGFBP3 | IBP3_HUMAN |
| P01344 | Insulin-like growth factor II (IGF-II) (Somatomedin-A) (T3M-11-derived growth factor) [Cleaved into: Insulin-like growth factor II; Insulin-like growth factor II Ala-25 Del; Preptin] | IGF2 | IGF2_HUMAN |
| P01579 | Interferon gamma (IFN-gamma) (Immune interferon) | IFNG | IFNG_HUMAN |
| P05362 | Intercellular adhesion molecule 1 (ICAM-1) (Major group rhinovirus receptor) (CD antigen CD54) | ICAM1 | ICAM1_HUMAN |
| P04792 | Heat shock protein beta-1 (HspB1) (28 kDa heat shock protein) (Estrogen-regulated 24 kDa protein) (Heat shock 27 kDa protein) (HSP 27) (Stress-responsive protein 27) (SRP27) | HSPB1 | HSPB1_HUMAN |
| P11021 | Endoplasmic reticulum chaperone BiP (EC 3.6.4.10) (78 kDa glucose-regulated protein) (GRP-78) (Binding-immunoglobulin protein) (BiP) (Heat shock protein 70 family protein 5) (HSP70 family protein 5) (Heat shock protein family A member 5) (Immunoglobulin heavy chain-binding protein) | HSPA5 | BIP_HUMAN |
| Q14568 | Heat shock protein HSP 90-alpha A2 (Heat shock 90 kDa protein 1 alpha-like 3) (Heat shock protein HSP 90-alpha A2 pseudogene) | HSP90AA2P | HS902_HUMAN |
| Q00613 | Heat shock factor protein 1 (HSF 1) (Heat shock transcription factor 1) (HSTF 1) | HSF1 | HSF1_HUMAN |
| P09601 | Heme oxygenase 1 (HO-1) (EC 1.14.14.18) | HMOX1 | HMOX1_HUMAN |
| P52789 | Hexokinase-2 (EC 2.7.1.1) (Hexokinase type II) (HK II) (Hexokinase-B) (Muscle form hexokinase) | HK2 | HXK2_HUMAN |
| Q16665 | Hypoxia-inducible factor 1-alpha (HIF-1-alpha) (HIF1-alpha) (ARNT-interacting protein) (Basic-helix-loop-helix-PAS protein MOP1) (Class E basic helix-loop-helix protein 78) (bHLHe78) (Member of PAS protein 1) (PAS domain-containing protein 8) | HIF1A | HIF1A_HUMAN |
| Q92819 | Hyaluronan synthase 2 (EC 2.4.1.212) (Hyaluronate synthase 2) (Hyaluronic acid synthase 2) (HA synthase 2) | HAS2 | HYAS2_HUMAN |
| P09211 | Glutathione S-transferase P (EC 2.5.1.18) (GST class-pi) (GSTP1-1) | GSTP1 | GSTP1_HUMAN |
| P28161 | Glutathione S-transferase Mu 2 (EC 2.5.1.18) (GST class-mu 2) (GSTM2-2) | GSTM2 | GSTM2_HUMAN |
| P09488 | Glutathione S-transferase Mu 1 (EC 2.5.1.18) (GST HB subunit 4) (GST class-mu 1) (GSTM1-1) (GSTM1a-1a) (GSTM1b-1b) (GTH4) | GSTM1 | GSTM1_HUMAN |
| P49841 | Glycogen synthase kinase-3 beta (GSK-3 beta) (EC 2.7.11.26) (Serine/threonine-protein kinase GSK3B) (EC 2.7.11.1) | GSK3B | GSK3B_HUMAN |
| P42262 | Glutamate receptor 2 (GluR-2) (AMPA-selective glutamate receptor 2) (GluR-B) (GluR-K2) (Glutamate receptor ionotropic, AMPA 2) (GluA2) | GRIA2 | GRIA2_HUMAN |
| P17302 | Gap junction alpha-1 protein (Connexin-43) (Cx43) (Gap junction 43 kDa heart protein) | GJA1 | CXA1_HUMAN |
| P14867 | Gamma-aminobutyric acid receptor subunit alpha-1 (GABA(A) receptor subunit alpha-1) | GABRA1 | GBRA1_HUMAN |
| P01100 | Proto-oncogene c-Fos (Cellular oncogene fos) (G0/G1 switch regulatory protein 7) | FOS | FOS_HUMAN |
| P49327 | Fatty acid synthase (EC 2.3.1.85) (Type I fatty acid synthase) [Includes: [Acyl-carrier-protein] S-acetyltransferase (EC 2.3.1.38); [Acyl-carrier-protein] S-malonyltransferase (EC 2.3.1.39); 3-oxoacyl-[acyl-carrier-protein] synthase (EC 2.3.1.41); 3-oxoacyl-[acyl-carrier-protein] reductase (EC 1.1.1.100); 3-hydroxyacyl-[acyl-carrier-protein] dehydratase (EC 4.2.1.59); Enoyl-[acyl-carrier-protein] reductase (EC 1.3.1.39); Acyl-[acyl-carrier-protein] hydrolase (EC 3.1.2.14)] | FASN | FAS_HUMAN |
| P08709 | Coagulation factor VII (EC 3.4.21.21) (Proconvertin) (Serum prothrombin conversion accelerator) (SPCA) (Eptacog alfa) [Cleaved into: Factor VII light chain; Factor VII heavy chain] | F7 | FA7_HUMAN |
| P13726 | Tissue factor (TF) (Coagulation factor III) (Thromboplastin) (CD antigen CD142) | F3 | TF_HUMAN |
| Q92731 | Estrogen receptor beta (ER-beta) (Nuclear receptor subfamily 3 group A member 2) | ESR2 | ESR2_HUMAN |
| P03372 | Estrogen receptor (ER) (ER-alpha) (Estradiol receptor) (Nuclear receptor subfamily 3 group A member 1) | ESR1 | ESR1_HUMAN |
| P21860 | Receptor tyrosine-protein kinase erbB-3 (EC 2.7.10.1) (Proto-oncogene-like protein c-ErbB-3) (Tyrosine kinase-type cell surface receptor HER3) | ERBB3 | ERBB3_HUMAN |
| P04626 | Receptor tyrosine-protein kinase erbB-2 (EC 2.7.10.1) (Metastatic lymph node gene 19 protein) (MLN 19) (Proto-oncogene Neu) (Proto-oncogene c-ErbB-2) (Tyrosine kinase-type cell surface receptor HER2) (p185erbB2) (CD antigen CD340) | ERBB2 | ERBB2_HUMAN |
| P19419 | ETS domain-containing protein Elk-1 | ELK1 | ELK1_HUMAN |
| P56537 | Eukaryotic translation initiation factor 6 (eIF-6) (B(2)GCN homolog) (B4 integrin interactor) (CAB) (p27(BBP)) | EIF6 | IF6_HUMAN |
| P00533 | Epidermal growth factor receptor (EC 2.7.10.1) (Proto-oncogene c-ErbB-1) (Receptor tyrosine-protein kinase erbB-1) | EGFR | EGFR_HUMAN |
| P01133 | Pro-epidermal growth factor (EGF) [Cleaved into: Epidermal growth factor (Urogastrone)] | EGF | EGF_HUMAN |
| Q14209 | Transcription factor E2F2 (E2F-2) | E2F2 | E2F2_HUMAN |
| Q01094 | Transcription factor E2F1 (E2F-1) (PBR3) (Retinoblastoma-associated protein 1) (RBAP-1) (Retinoblastoma-binding protein 3) (RBBP-3) (pRB-binding protein E2F-1) | E2F1 | E2F1_HUMAN |
| Q9NRD8 | Dual oxidase 2 (EC 1.11.1.-) (EC 1.6.3.1) (Large NOX 2) (Long NOX 2) (NADH/NADPH thyroid oxidase p138-tox) (NADPH oxidase/peroxidase DUOX2) (NADPH thyroid oxidase 2) (Thyroid oxidase 2) (p138 thyroid oxidase) | DUOX2 | DUOX2_HUMAN |
| P21728 | D(1A) dopamine receptor (Dopamine D1 receptor) | DRD1 | DRD1_HUMAN |
| P27487 | Dipeptidyl peptidase 4 (EC 3.4.14.5) (ADABP) (Adenosine deaminase complexing protein 2) (ADCP-2) (Dipeptidyl peptidase IV) (DPP IV) (T-cell activation antigen CD26) (TP103) (CD antigen CD26) [Cleaved into: Dipeptidyl peptidase 4 membrane form (Dipeptidyl peptidase IV membrane form); Dipeptidyl peptidase 4 soluble form (Dipeptidyl peptidase IV soluble form)] | DPP4 | DPP4_HUMAN |
| P16444 | Dipeptidase 1 (EC 3.4.13.19) (Beta-lactamase) (EC 3.5.2.6) (Dehydropeptidase-I) (Microsomal dipeptidase) (Renal dipeptidase) (hRDP) | DPEP1 | DPEP1_HUMAN |
| P49895 | Type I iodothyronine deiodinase (EC 1.21.99.4) (5DI) (DIOI) (Type 1 DI) (Type-I 5'-deiodinase) | DIO1 | IOD1_HUMAN |
| Q96JK2 | DDB1- and CUL4-associated factor 5 (Breakpoint cluster region protein 2) (BCRP2) (WD repeat-containing protein 22) | DCAF5 | DCAF5_HUMAN |
| P08684 | Cytochrome P450 3A4 (EC 1.14.14.1) (1,4-cineole 2-exo-monooxygenase) (1,8-cineole 2-exo-monooxygenase) (EC 1.14.14.56) (Albendazole monooxygenase (sulfoxide-forming)) (EC 1.14.14.73) (Albendazole sulfoxidase) (CYPIIIA3) (CYPIIIA4) (Cholesterol 25-hydroxylase) (Cytochrome P450 3A3) (Cytochrome P450 HLp) (Cytochrome P450 NF-25) (Cytochrome P450-PCN1) (Nifedipine oxidase) (Quinine 3-monooxygenase) (EC 1.14.14.55) | CYP3A4 | CP3A4_HUMAN |
| Q16678 | Cytochrome P450 1B1 (EC 1.14.14.1) (CYPIB1) (Hydroperoxy icosatetraenoate dehydratase) (EC 4.2.1.152) | CYP1B1 | CP1B1_HUMAN |
| P05177 | Cytochrome P450 1A2 (EC 1.14.14.1) (CYPIA2) (Cholesterol 25-hydroxylase) (Cytochrome P(3)450) (Cytochrome P450 4) (Cytochrome P450-P3) (Hydroperoxy icosatetraenoate dehydratase) (EC 4.2.1.152) | CYP1A2 | CP1A2_HUMAN |
| P04798 | Cytochrome P450 1A1 (CYPIA1) (EC 1.14.14.1) (Cytochrome P450 form 6) (Cytochrome P450-C) (Cytochrome P450-P1) (Hydroperoxy icosatetraenoate dehydratase) (EC 4.2.1.152) | CYP1A1 | CP1A1_HUMAN |
| P10145 | Interleukin-8 (IL-8) (C-X-C motif chemokine 8) (Chemokine (C-X-C motif) ligand 8) (Emoctakin) (Granulocyte chemotactic protein 1) (GCP-1) (Monocyte-derived neutrophil chemotactic factor) (MDNCF) (Monocyte-derived neutrophil-activating peptide) (MONAP) (Neutrophil-activating protein 1) (NAP-1) (Protein 3-10C) (T-cell chemotactic factor) [Cleaved into: MDNCF-a (GCP/IL-8 protein IV) (IL8/NAP1 form I); Interleukin-8 ((Ala-IL-8)77) (GCP/IL-8 protein II) (IL-8(1-77)) (IL8/NAP1 form II) (MDNCF-b); IL-8(5-77); IL-8(6-77) ((Ser-IL-8)72) (GCP/IL-8 protein I) (IL8/NAP1 form III) (Lymphocyte-derived neutrophil-activating factor) (LYNAP) (MDNCF-c) (Neutrophil-activating factor) (NAF); IL-8(7-77) (GCP/IL-8 protein V) (IL8/NAP1 form IV); IL-8(8-77) (GCP/IL-8 protein VI) (IL8/NAP1 form V); IL-8(9-77) (GCP/IL-8 protein III) (IL8/NAP1 form VI)] | CXCL8 | IL8_HUMAN |
| P19875 | C-X-C motif chemokine 2 (Growth-regulated protein beta) (Gro-beta) (Macrophage inflammatory protein 2-alpha) (MIP2-alpha) [Cleaved into: GRO-beta(5-73) (GRO-beta-T) (Hematopoietic synergistic factor) (HSF) (SB-251353)] | CXCL2 | CXCL2_HUMAN |
| O14625 | C-X-C motif chemokine 11 (Beta-R1) (H174) (Interferon gamma-inducible protein 9) (IP-9) (Interferon-inducible T-cell alpha chemoattractant) (I-TAC) (Small-inducible cytokine B11) | CXCL11 | CXL11_HUMAN |
| P02778 | C-X-C motif chemokine 10 (10 kDa interferon gamma-induced protein) (Gamma-IP10) (IP-10) (Small-inducible cytokine B10) [Cleaved into: CXCL10(1-73)] | CXCL10 | CXL10_HUMAN |
| P07339 | Cathepsin D (EC 3.4.23.5) [Cleaved into: Cathepsin D light chain; Cathepsin D heavy chain] | CTSD | CATD_HUMAN |
| P17538 | Chymotrypsinogen B (EC 3.4.21.1) [Cleaved into: Chymotrypsin B chain A; Chymotrypsin B chain B; Chymotrypsin B chain C] | CTRB1 | CTRB1_HUMAN |
| Q9Y215 | Acetylcholinesterase collagenic tail peptide (AChE Q subunit) (Acetylcholinesterase-associated collagen) | COLQ | COLQ_HUMAN |
| P02461 | Collagen alpha-1(III) chain | COL3A1 | CO3A1_HUMAN |
| P02452 | Collagen alpha-1(I) chain (Alpha-1 type I collagen) | COL1A1 | CO1A1_HUMAN |
| O14493 | Claudin-4 (Clostridium perfringens enterotoxin receptor) (CPE-R) (CPE-receptor) (Williams-Beuren syndrome chromosomal region 8 protein) | CLDN4 | CLD4_HUMAN |
| O15111 | Inhibitor of nuclear factor kappa-B kinase subunit alpha (I-kappa-B kinase alpha) (IKK-A) (IKK-alpha) (IkBKA) (IkappaB kinase) (EC 2.7.11.10) (Conserved helix-loop-helix ubiquitous kinase) (I-kappa-B kinase 1) (IKK1) (Nuclear factor NF-kappa-B inhibitor kinase alpha) (NFKBIKA) (Transcription factor 16) (TCF-16) | CHUK | IKKA_HUMAN |
| Q15822 | Neuronal acetylcholine receptor subunit alpha-2 | CHRNA2 | ACHA2_HUMAN |
| P08912 | Muscarinic acetylcholine receptor M5 | CHRM5 | ACM5_HUMAN |
| P08173 | Muscarinic acetylcholine receptor M4 | CHRM4 | ACM4_HUMAN |
| P20309 | Muscarinic acetylcholine receptor M3 | CHRM3 | ACM3_HUMAN |
| P08172 | Muscarinic acetylcholine receptor M2 | CHRM2 | ACM2_HUMAN |
| P11229 | Muscarinic acetylcholine receptor M1 | CHRM1 | ACM1_HUMAN |
| O96017 | Serine/threonine-protein kinase Chk2 (EC 2.7.11.1) (CHK2 checkpoint homolog) (Cds1 homolog) (Hucds1) (hCds1) (Checkpoint kinase 2) | CHEK2 | CHK2_HUMAN |
| P38936 | Cyclin-dependent kinase inhibitor 1 (CDK-interacting protein 1) (Melanoma differentiation-associated protein 6) (MDA-6) (p21) | CDKN1A | CDN1A_HUMAN |
| P24941 | Cyclin-dependent kinase 2 (EC 2.7.11.22) (Cell division protein kinase 2) (p33 protein kinase) | CDK2 | CDK2_HUMAN |
| P06493 | Cyclin-dependent kinase 1 (CDK1) (EC 2.7.11.22) (EC 2.7.11.23) (Cell division control protein 2 homolog) (Cell division protein kinase 1) (p34 protein kinase) | CDK1 | CDK1_HUMAN |
| P29965 | CD40 ligand (CD40-L) (T-cell antigen Gp39) (TNF-related activation protein) (TRAP) (Tumor necrosis factor ligand superfamily member 5) (CD antigen CD154) [Cleaved into: CD40 ligand, membrane form; CD40 ligand, soluble form (sCD40L)] | CD40LG | CD40L_HUMAN |
| P24385 | G1/S-specific cyclin-D1 (B-cell lymphoma 1 protein) (BCL-1) (BCL-1 oncogene) (PRAD1 oncogene) | CCND1 | CCND1_HUMAN |
| P14635 | G2/mitotic-specific cyclin-B1 | CCNB1 | CCNB1_HUMAN |
| P20248 | Cyclin-A2 (Cyclin-A) (Cyclin A) | CCNA2 | CCNA2_HUMAN |
| P13500 | C-C motif chemokine 2 (HC11) (Monocyte chemoattractant protein 1) (Monocyte chemotactic and activating factor) (MCAF) (Monocyte chemotactic protein 1) (MCP-1) (Monocyte secretory protein JE) (Small-inducible cytokine A2) | CCL2 | CCL2_HUMAN |
| Q03135 | Caveolin-1 | CAV1 | CAV1_HUMAN |
| P04040 | Catalase (EC 1.11.1.6) | CAT | CATA_HUMAN |
| P55211 | Caspase-9 (CASP-9) (EC 3.4.22.62) (Apoptotic protease Mch-6) (Apoptotic protease-activating factor 3) (APAF-3) (ICE-like apoptotic protease 6) (ICE-LAP6) [Cleaved into: Caspase-9 subunit p35; Caspase-9 subunit p10] | CASP9 | CASP9_HUMAN |
| Q14790 | Caspase-8 (CASP-8) (EC 3.4.22.61) (Apoptotic cysteine protease) (Apoptotic protease Mch-5) (CAP4) (FADD-homologous ICE/ced-3-like protease) (FADD-like ICE) (FLICE) (ICE-like apoptotic protease 5) (MORT1-associated ced-3 homolog) (MACH) [Cleaved into: Caspase-8 subunit p18; Caspase-8 subunit p10] | CASP8 | CASP8_HUMAN |
| P42574 | Caspase-3 (CASP-3) (EC 3.4.22.56) (Apopain) (Cysteine protease CPP32) (CPP-32) (Protein Yama) (SREBP cleavage activity 1) (SCA-1) [Cleaved into: Caspase-3 subunit p17; Caspase-3 subunit p12] | CASP3 | CASP3_HUMAN |
| P27482 | Calmodulin-like protein 3 (CaM-like protein) (CLP) (Calmodulin-related protein NB-1) | CALML3 | CALL3_HUMAN |
| P00918 | Carbonic anhydrase 2 (EC 4.2.1.1) (Carbonate dehydratase II) (Carbonic anhydrase C) (CAC) (Carbonic anhydrase II) (CA-II) | CA2 | CAH2_HUMAN |
| O15392 | Baculoviral IAP repeat-containing protein 5 (Apoptosis inhibitor 4) (Apoptosis inhibitor survivin) | BIRC5 | BIRC5_HUMAN |
| Q07817 | Bcl-2-like protein 1 (Bcl2-L-1) (Apoptosis regulator Bcl-X) | BCL2L1 | B2CL1_HUMAN |
| P10415 | Apoptosis regulator Bcl-2 | BCL2 | BCL2_HUMAN |
| Q07812 | Apoptosis regulator BAX (Bcl-2-like protein 4) (Bcl2-L-4) | BAX | BAX_HUMAN |
| Q9Y5Z0 | Beta-secretase 2 (EC 3.4.23.45) (Aspartic-like protease 56 kDa) (Aspartyl protease 1) (ASP1) (Asp 1) (Beta-site amyloid precursor protein cleaving enzyme 2) (Beta-site APP cleaving enzyme 2) (Down region aspartic protease) (DRAP) (Memapsin-1) (Membrane-associated aspartic protease 1) (Theta-secretase) | BACE2 | BACE2_HUMAN |
| P10275 | Androgen receptor (Dihydrotestosterone receptor) (Nuclear receptor subfamily 3 group C member 4) | AR | ANDR_HUMAN |
| P09917 | Polyunsaturated fatty acid 5-lipoxygenase (EC 1.13.11.-) (Arachidonate 5-lipoxygenase) (5-LO) (5-lipoxygenase) (EC 1.13.11.34) | ALOX5 | LOX5_HUMAN |
| P31749 | RAC-alpha serine/threonine-protein kinase (EC 2.7.11.1) (Protein kinase B) (PKB) (Protein kinase B alpha) (PKB alpha) (Proto-oncogene c-Akt) (RAC-PK-alpha) | AKT1 | AKT1_HUMAN |
| P42330 | Aldo-keto reductase family 1 member C3 (EC 1.-.-.-) (17-beta-hydroxysteroid dehydrogenase type 5) (17-beta-HSD 5) (3-alpha-HSD type II, brain) (3-alpha-hydroxysteroid dehydrogenase type 2) (3-alpha-HSD type 2) (EC 1.1.1.357) (Chlordecone reductase homolog HAKRb) (Dihydrodiol dehydrogenase 3) (DD-3) (DD3) (Dihydrodiol dehydrogenase type I) (HA1753) (Indanol dehydrogenase) (EC 1.1.1.112) (Prostaglandin F synthase) (PGFS) (EC 1.1.1.188) (Testosterone 17-beta-dehydrogenase 5) (EC 1.1.1.239) (EC 1.1.1.64) (Trans-1,2-dihydrobenzene-1,2-diol dehydrogenase) (EC 1.3.1.20) | AKR1C3 | AK1C3_HUMAN |
| P15121 | Aldo-keto reductase family 1 member B1 (EC 1.1.1.300) (EC 1.1.1.372) (EC 1.1.1.54) (Aldehyde reductase) (Aldose reductase) (AR) (EC 1.1.1.21) | AKR1B1 | ALDR_HUMAN |
| O95433 | Activator of 90 kDa heat shock protein ATPase homolog 1 (AHA1) (p38) | AHSA1 | AHSA1_HUMAN |
| P35869 | Aryl hydrocarbon receptor (Ah receptor) (AhR) (Class E basic helix-loop-helix protein 76) (bHLHe76) | AHR | AHR_HUMAN |
| P07550 | Beta-2 adrenergic receptor (Beta-2 adrenoreceptor) (Beta-2 adrenoceptor) | ADRB2 | ADRB2_HUMAN |
| P08588 | Beta-1 adrenergic receptor (Beta-1 adrenoreceptor) (Beta-1 adrenoceptor) | ADRB1 | ADRB1_HUMAN |
| P18825 | Alpha-2C adrenergic receptor (Alpha-2 adrenergic receptor subtype C4) (Alpha-2C adrenoreceptor) (Alpha-2C adrenoceptor) (Alpha-2CAR) | ADRA2C | ADA2C_HUMAN |
| P08913 | Alpha-2A adrenergic receptor (Alpha-2 adrenergic receptor subtype C10) (Alpha-2A adrenoreceptor) (Alpha-2A adrenoceptor) (Alpha-2AAR) | ADRA2A | ADA2A_HUMAN |
| P25100 | Alpha-1D adrenergic receptor (Alpha-1A adrenergic receptor) (Alpha-1D adrenoreceptor) (Alpha-1D adrenoceptor) (Alpha-adrenergic receptor 1a) | ADRA1D,ADRA1A | ADA1D_HUMAN |
| P35368 | Alpha-1B adrenergic receptor (Alpha-1B adrenoreceptor) (Alpha-1B adrenoceptor) | ADRA1B | ADA1B_HUMAN |
| P35348 | Alpha-1A adrenergic receptor (Alpha-1A adrenoreceptor) (Alpha-1A adrenoceptor) (Alpha-1C adrenergic receptor) (Alpha-adrenergic receptor 1c) | ADRA1A | ADA1A_HUMAN |
| P00326 | Alcohol dehydrogenase 1C (EC 1.1.1.1) (Alcohol dehydrogenase subunit gamma) | ADH1C | ADH1G_HUMAN |
| P00325 | All-trans-retinol dehydrogenase [NAD(+)] ADH1B (EC 1.1.1.105) (Alcohol dehydrogenase 1B) (Alcohol dehydrogenase subunit beta) | ADH1B | ADH1B_HUMAN |
| P07327 | Alcohol dehydrogenase 1A (EC 1.1.1.1) (Alcohol dehydrogenase subunit alpha) | ADH1A | ADH1A_HUMAN |
| P15309 | Prostatic acid phosphatase (PAP) (EC 3.1.3.2) (5'-nucleotidase) (5'-NT) (EC 3.1.3.5) (Acid phosphatase 3) (Ecto-5'-nucleotidase) (Thiamine monophosphatase) (TMPase) [Cleaved into: PAPf39] | ACP3 | PPAP_HUMAN |
| Q13085 | Acetyl-CoA carboxylase 1 (ACC1) (EC 6.4.1.2) (Acetyl-Coenzyme A carboxylase alpha) (ACC-alpha) | ACACA | ACACA_HUMAN |
| Q9UNQ0 | Broad substrate specificity ATP-binding cassette transporter ABCG2 (EC 7.6.2.2) (ATP-binding cassette sub-family G member 2) (Breast cancer resistance protein) (CDw338) (Mitoxantrone resistance-associated protein) (Placenta-specific ATP-binding cassette transporter) (Urate exporter) (CD antigen CD338) | ABCG2 | ABCG2_HUMAN |
| Q92887 | Canalicular multispecific organic anion transporter 1 (ATP-binding cassette sub-family C member 2) (Canalicular multidrug resistance protein) (Multidrug resistance-associated protein 2) (EC 7.6.2.2) | ABCC2 | MRP2_HUMAN |
