# Supplementary material for: Exploring the Molecular Mechanism of Liuwei Dihuang Pills for Treating Diabetic Nephropathy by Combined Network Pharmacology and Molecular Docking
Source: Evid Based Complement Alternat Med. 2021 Sep 13;2021:7262208. doi: 10.1155/2021/7262208 (PMC8452392; doi:10.1155/2021/7262208)
Supplement: Supplementary Materials — Table S1: 186 putative targets of LDP. Table S2: 3701 disease targets of DN. Table S3: 131 common targets of LDP and DN. Table S4: GO functional enrichment analysis. Table S5: KEGG pathway enrichment analysis. Figures S1–S4: heatmap of the enrichment analysis results downloaded from Metascape online platform. [file 7262208.f1.zip › 7262208.f1/Table S4 GO functional enrichment analysis.docx]

Table S4: GO functional enrichment analysis

| **Category** | **GO** | **Description** | **#GeneInGOAndHitList** | **_LogP_MyList** |
| --- | --- | --- | --- | --- |
| GO Biological Processes | GO:0010035 | response to inorganic substance | 44 | -40.72764332 |
| GO Biological Processes | GO:0009636 | response to toxic substance | 40 | -36.5823639 |
| GO Biological Processes | GO:0006979 | response to oxidative stress | 37 | -34.47397619 |
| GO Biological Processes | GO:0009611 | response to wounding | 42 | -33.94919454 |
| GO Biological Processes | GO:0010038 | response to metal ion | 34 | -33.73480075 |
| GO Biological Processes | GO:0009991 | response to extracellular stimulus | 38 | -33.05314216 |
| GO Biological Processes | GO:0071407 | cellular response to organic cyclic compound | 37 | -32.73374664 |
| GO Biological Processes | GO:1901699 | cellular response to nitrogen compound | 40 | -32.43903169 |
| GO Biological Processes | GO:0031667 | response to nutrient levels | 36 | -31.35437411 |
| GO Biological Processes | GO:0000302 | response to reactive oxygen species | 28 | -30.66948837 |
| GO Biological Processes | GO:0032496 | response to lipopolysaccharide | 31 | -30.65548646 |
| GO Biological Processes | GO:0034599 | cellular response to oxidative stress | 30 | -30.10682306 |
| GO Biological Processes | GO:0002237 | response to molecule of bacterial origin | 31 | -29.79043553 |
| GO Biological Processes | GO:0071396 | cellular response to lipid | 36 | -29.26786603 |
| GO Biological Processes | GO:0071417 | cellular response to organonitrogen compound | 36 | -28.93574316 |
| GO Biological Processes | GO:0097190 | apoptotic signaling pathway | 36 | -28.71035754 |
| GO Biological Processes | GO:0042060 | wound healing | 35 | -27.92589539 |
| GO Biological Processes | GO:0070482 | response to oxygen levels | 30 | -26.99345446 |
| GO Biological Processes | GO:0009410 | response to xenobiotic stimulus | 27 | -26.86263411 |
| GO Biological Processes | GO:0072593 | reactive oxygen species metabolic process | 27 | -26.73862405 |
| GO Biological Processes | GO:0048545 | response to steroid hormone | 28 | -25.88182925 |
| GO Biological Processes | GO:1901652 | response to peptide | 32 | -25.49575217 |
| GO Biological Processes | GO:2000147 | positive regulation of cell motility | 33 | -25.44936161 |
| GO Biological Processes | GO:0051272 | positive regulation of cellular component movement | 33 | -25.03611288 |
| GO Biological Processes | GO:0035690 | cellular response to drug | 28 | -24.91557628 |
| GO Biological Processes | GO:0030335 | positive regulation of cell migration | 32 | -24.83894273 |
| GO Biological Processes | GO:0040017 | positive regulation of locomotion | 33 | -24.81226173 |
| GO Biological Processes | GO:0001101 | response to acid chemical | 27 | -24.55078391 |
| GO Biological Processes | GO:0010942 | positive regulation of cell death | 35 | -24.5131575 |
| GO Biological Processes | GO:0001666 | response to hypoxia | 27 | -24.15557073 |
| GO Biological Processes | GO:2001233 | regulation of apoptotic signaling pathway | 28 | -23.82303103 |
| GO Biological Processes | GO:0036293 | response to decreased oxygen levels | 27 | -23.77449883 |
| GO Biological Processes | GO:0050673 | epithelial cell proliferation | 29 | -23.66441974 |
| GO Biological Processes | GO:0044706 | multi-multicellular organism process | 23 | -23.57161615 |
| GO Biological Processes | GO:0032870 | cellular response to hormone stimulus | 33 | -23.56739062 |
| GO Biological Processes | GO:0034614 | cellular response to reactive oxygen species | 21 | -23.33025066 |
| GO Biological Processes | GO:1901654 | response to ketone | 22 | -23.30149993 |
| GO Biological Processes | GO:0046677 | response to antibiotic | 25 | -22.55288548 |
| GO Biological Processes | GO:0071241 | cellular response to inorganic substance | 22 | -22.33057054 |
| GO Biological Processes | GO:0007565 | female pregnancy | 21 | -21.99794105 |
| GO Biological Processes | GO:2001234 | negative regulation of apoptotic signaling pathway | 22 | -21.8199695 |
| GO Biological Processes | GO:0071496 | cellular response to external stimulus | 25 | -21.70003076 |
| GO Biological Processes | GO:0050678 | regulation of epithelial cell proliferation | 26 | -21.49198474 |
| GO Biological Processes | GO:0001568 | blood vessel development | 33 | -21.46874845 |
| GO Biological Processes | GO:0009617 | response to bacterium | 32 | -21.0652276 |
| GO Biological Processes | GO:0018209 | peptidyl-serine modification | 24 | -21.00467694 |
| GO Biological Processes | GO:0007584 | response to nutrient | 21 | -20.97138221 |
| GO Biological Processes | GO:0071248 | cellular response to metal ion | 20 | -20.65941116 |
| GO Biological Processes | GO:0018105 | peptidyl-serine phosphorylation | 23 | -20.41112268 |
| GO Biological Processes | GO:0009314 | response to radiation | 26 | -20.38617202 |
| GO Biological Processes | GO:2000377 | regulation of reactive oxygen species metabolic process | 20 | -20.3454846 |
| GO Biological Processes | GO:0032355 | response to estradiol | 18 | -20.2829151 |
| GO Biological Processes | GO:0030155 | regulation of cell adhesion | 31 | -20.25405105 |
| GO Biological Processes | GO:0070997 | neuron death | 24 | -20.2073315 |
| GO Biological Processes | GO:0033002 | muscle cell proliferation | 21 | -19.98120223 |
| GO Biological Processes | GO:0071216 | cellular response to biotic stimulus | 21 | -19.90681723 |
| GO Biological Processes | GO:0043068 | positive regulation of programmed cell death | 30 | -19.89555441 |
| GO Biological Processes | GO:0071466 | cellular response to xenobiotic stimulus | 19 | -19.74194336 |
| GO Biological Processes | GO:0048514 | blood vessel morphogenesis | 30 | -19.70398224 |
| GO Biological Processes | GO:0008285 | negative regulation of cell proliferation | 31 | -19.43075044 |
| GO Biological Processes | GO:0046686 | response to cadmium ion | 14 | -19.33332978 |
| GO Biological Processes | GO:0048511 | rhythmic process | 22 | -19.22646303 |
| GO Biological Processes | GO:0097191 | extrinsic apoptotic signaling pathway | 20 | -19.12377429 |
| GO Biological Processes | GO:0051090 | regulation of DNA-binding transcription factor activity | 25 | -19.02130709 |
| GO Biological Processes | GO:0070848 | response to growth factor | 30 | -19.00197063 |
| GO Biological Processes | GO:0043065 | positive regulation of apoptotic process | 29 | -18.96819809 |
| GO Biological Processes | GO:0040008 | regulation of growth | 29 | -18.93395019 |
| GO Biological Processes | GO:0050878 | regulation of body fluid levels | 26 | -18.85585472 |
| GO Biological Processes | GO:0034612 | response to tumor necrosis factor | 22 | -18.80688179 |
| GO Biological Processes | GO:0071276 | cellular response to cadmium ion | 12 | -18.79385823 |
| GO Biological Processes | GO:1901214 | regulation of neuron death | 22 | -18.7776828 |
| GO Biological Processes | GO:0003006 | developmental process involved in reproduction | 29 | -18.68057848 |
| GO Biological Processes | GO:0048660 | regulation of smooth muscle cell proliferation | 18 | -18.64315721 |
| GO Biological Processes | GO:0048659 | smooth muscle cell proliferation | 18 | -18.5519362 |
| GO Biological Processes | GO:0009612 | response to mechanical stimulus | 19 | -18.54285849 |
| GO Biological Processes | GO:0062012 | regulation of small molecule metabolic process | 25 | -18.44367685 |
| GO Biological Processes | GO:0002521 | leukocyte differentiation | 26 | -18.38808913 |
| GO Biological Processes | GO:0001525 | angiogenesis | 27 | -18.13626939 |
| GO Biological Processes | GO:0048608 | reproductive structure development | 24 | -18.09147256 |
| GO Biological Processes | GO:0048732 | gland development | 24 | -18.09147256 |
| GO Biological Processes | GO:0061458 | reproductive system development | 24 | -18.001961 |
| GO Biological Processes | GO:0043434 | response to peptide hormone | 24 | -17.97972072 |
| GO Biological Processes | GO:1903409 | reactive oxygen species biosynthetic process | 16 | -17.90801784 |
| GO Biological Processes | GO:1903034 | regulation of response to wounding | 18 | -17.6177251 |
| GO Biological Processes | GO:2001237 | negative regulation of extrinsic apoptotic signaling pathway | 15 | -17.5852987 |
| GO Biological Processes | GO:0007568 | aging | 21 | -17.56763171 |
| GO Biological Processes | GO:0061041 | regulation of wound healing | 17 | -17.54807426 |
| GO Biological Processes | GO:0071363 | cellular response to growth factor stimulus | 28 | -17.38707523 |
| GO Biological Processes | GO:0031668 | cellular response to extracellular stimulus | 20 | -17.36960311 |
| GO Biological Processes | GO:1903037 | regulation of leukocyte cell-cell adhesion | 21 | -17.29350906 |
| GO Biological Processes | GO:0045785 | positive regulation of cell adhesion | 23 | -17.27903585 |
| GO Biological Processes | GO:0032787 | monocarboxylic acid metabolic process | 27 | -17.22456135 |
| GO Biological Processes | GO:0071222 | cellular response to lipopolysaccharide | 18 | -17.19243243 |
| GO Biological Processes | GO:0097193 | intrinsic apoptotic signaling pathway | 20 | -17.1314092 |
| GO Biological Processes | GO:0022407 | regulation of cell-cell adhesion | 23 | -16.99348365 |
| GO Biological Processes | GO:0007169 | transmembrane receptor protein tyrosine kinase signaling pathway | 28 | -16.86838925 |
| GO Biological Processes | GO:0071219 | cellular response to molecule of bacterial origin | 18 | -16.68532307 |
| GO Biological Processes | GO:1901216 | positive regulation of neuron death | 14 | -16.62273544 |
| GO Biological Processes | GO:0006367 | transcription initiation from RNA polymerase II promoter | 17 | -16.53726903 |
| GO Biological Processes | GO:0080135 | regulation of cellular response to stress | 28 | -16.50359012 |
| GO Biological Processes | GO:0007159 | leukocyte cell-cell adhesion | 21 | -16.40138532 |
| GO Biological Processes | GO:0051098 | regulation of binding | 21 | -16.3054724 |
| GO Biological Processes | GO:0071900 | regulation of protein serine/threonine kinase activity | 24 | -16.30309257 |
| GO Biological Processes | GO:0009416 | response to light stimulus | 20 | -16.2981801 |
| GO Biological Processes | GO:1903039 | positive regulation of leukocyte cell-cell adhesion | 18 | -16.24474956 |
| GO Biological Processes | GO:2001236 | regulation of extrinsic apoptotic signaling pathway | 16 | -16.23634 |
| GO Biological Processes | GO:0042110 | T cell activation | 23 | -16.11131706 |
| GO Biological Processes | GO:0008015 | blood circulation | 24 | -16.01340267 |
| GO Biological Processes | GO:0071214 | cellular response to abiotic stimulus | 20 | -15.96334936 |
| GO Biological Processes | GO:0104004 | cellular response to environmental stimulus | 20 | -15.96334936 |
| GO Biological Processes | GO:0003013 | circulatory system process | 24 | -15.81966312 |
| GO Biological Processes | GO:0006352 | DNA-templated transcription, initiation | 18 | -15.79910394 |
| GO Biological Processes | GO:0009411 | response to UV | 15 | -15.73364291 |
| GO Biological Processes | GO:0071356 | cellular response to tumor necrosis factor | 19 | -15.67882446 |
| GO Biological Processes | GO:0006631 | fatty acid metabolic process | 21 | -15.6646615 |
| GO Biological Processes | GO:0045596 | negative regulation of cell differentiation | 27 | -15.59648765 |
| GO Biological Processes | GO:0045765 | regulation of angiogenesis | 21 | -15.49072414 |
| GO Biological Processes | GO:0033273 | response to vitamin | 13 | -15.40362753 |
| GO Biological Processes | GO:0001936 | regulation of endothelial cell proliferation | 16 | -15.08908961 |
| GO Biological Processes | GO:0009266 | response to temperature stimulus | 17 | -14.97266003 |
| GO Biological Processes | GO:0022409 | positive regulation of cell-cell adhesion | 18 | -14.93001524 |
| GO Biological Processes | GO:0010817 | regulation of hormone levels | 23 | -14.91406624 |
| GO Biological Processes | GO:0030099 | myeloid cell differentiation | 21 | -14.89070891 |
| GO Biological Processes | GO:0031960 | response to corticosteroid | 15 | -14.73979491 |
| GO Biological Processes | GO:0070201 | regulation of establishment of protein localization | 25 | -14.70663546 |
| GO Biological Processes | GO:0046649 | lymphocyte activation | 26 | -14.68998279 |
| GO Biological Processes | GO:0098754 | detoxification | 14 | -14.64482945 |
| GO Biological Processes | GO:1901342 | regulation of vasculature development | 21 | -14.63604889 |
| GO Biological Processes | GO:0001935 | endothelial cell proliferation | 16 | -14.53487341 |
| GO Biological Processes | GO:2000379 | positive regulation of reactive oxygen species metabolic process | 13 | -14.51013883 |
| GO Biological Processes | GO:0097237 | cellular response to toxic substance | 17 | -14.49434213 |
| GO Biological Processes | GO:0031669 | cellular response to nutrient levels | 17 | -14.46556718 |
| GO Biological Processes | GO:0050900 | leukocyte migration | 22 | -14.4051069 |
| GO Biological Processes | GO:1901653 | cellular response to peptide | 20 | -14.39804662 |
| GO Biological Processes | GO:0003018 | vascular process in circulatory system | 15 | -14.31699324 |
| GO Biological Processes | GO:0048638 | regulation of developmental growth | 19 | -14.30558279 |
| GO Biological Processes | GO:0030879 | mammary gland development | 14 | -14.24186313 |
| GO Biological Processes | GO:0035296 | regulation of tube diameter | 14 | -14.19881844 |
| GO Biological Processes | GO:0050880 | regulation of blood vessel size | 14 | -14.19881844 |
| GO Biological Processes | GO:0097746 | regulation of blood vessel diameter | 14 | -14.19881844 |
| GO Biological Processes | GO:0007623 | circadian rhythm | 16 | -14.18032586 |
| GO Biological Processes | GO:0035150 | regulation of tube size | 14 | -14.15610097 |
| GO Biological Processes | GO:0010212 | response to ionizing radiation | 14 | -14.11370595 |
| GO Biological Processes | GO:0043200 | response to amino acid | 13 | -14.08871359 |
| GO Biological Processes | GO:1990748 | cellular detoxification | 13 | -14.08871359 |
| GO Biological Processes | GO:0051186 | cofactor metabolic process | 23 | -14.06371274 |
| GO Biological Processes | GO:0060135 | maternal process involved in female pregnancy | 11 | -14.03362522 |
| GO Biological Processes | GO:0010631 | epithelial cell migration | 19 | -14.02111212 |
| GO Biological Processes | GO:0090132 | epithelium migration | 19 | -13.95705086 |
| GO Biological Processes | GO:0051091 | positive regulation of DNA-binding transcription factor activity | 17 | -13.94152392 |
| GO Biological Processes | GO:0014074 | response to purine-containing compound | 14 | -13.90640839 |
| GO Biological Processes | GO:0043408 | regulation of MAPK cascade | 25 | -13.85049937 |
| GO Biological Processes | GO:0090130 | tissue migration | 19 | -13.83064029 |
| GO Biological Processes | GO:0048871 | multicellular organismal homeostasis | 21 | -13.76265754 |
| GO Biological Processes | GO:0006690 | icosanoid metabolic process | 13 | -13.69846644 |
| GO Biological Processes | GO:0071453 | cellular response to oxygen levels | 16 | -13.66915269 |
| GO Biological Processes | GO:0097305 | response to alcohol | 16 | -13.66915269 |
| GO Biological Processes | GO:0006805 | xenobiotic metabolic process | 13 | -13.51369672 |
| GO Biological Processes | GO:0062013 | positive regulation of small molecule metabolic process | 14 | -13.51353443 |
| GO Biological Processes | GO:1901655 | cellular response to ketone | 12 | -13.47425698 |
| GO Biological Processes | GO:0042759 | long-chain fatty acid biosynthetic process | 9 | -13.41252389 |
| GO Biological Processes | GO:0051402 | neuron apoptotic process | 16 | -13.38702718 |
| GO Biological Processes | GO:0032102 | negative regulation of response to external stimulus | 19 | -13.36603079 |
| GO Biological Processes | GO:0007596 | blood coagulation | 18 | -13.35500778 |
| GO Biological Processes | GO:1902893 | regulation of pri-miRNA transcription by RNA polymerase II | 10 | -13.32279921 |
| GO Biological Processes | GO:0007599 | hemostasis | 18 | -13.24772513 |
| GO Biological Processes | GO:0061614 | pri-miRNA transcription by RNA polymerase II | 10 | -13.23370225 |
| GO Biological Processes | GO:0050817 | coagulation | 18 | -13.22647273 |
| GO Biological Processes | GO:0045766 | positive regulation of angiogenesis | 15 | -13.20402953 |
| GO Biological Processes | GO:0072330 | monocarboxylic acid biosynthetic process | 18 | -13.16311665 |
| GO Biological Processes | GO:0048661 | positive regulation of smooth muscle cell proliferation | 12 | -13.15306874 |
| GO Biological Processes | GO:0051100 | negative regulation of binding | 14 | -13.14657628 |
| GO Biological Processes | GO:0002573 | myeloid leukocyte differentiation | 15 | -13.11256526 |
| GO Biological Processes | GO:0050679 | positive regulation of epithelial cell proliferation | 15 | -13.11256526 |
| GO Biological Processes | GO:0042063 | gliogenesis | 17 | -13.02825225 |
| GO Biological Processes | GO:0046394 | carboxylic acid biosynthetic process | 20 | -13.02048758 |
| GO Biological Processes | GO:0050727 | regulation of inflammatory response | 21 | -13.01059451 |
| GO Biological Processes | GO:0016053 | organic acid biosynthetic process | 20 | -13.00354845 |
| GO Biological Processes | GO:0045860 | positive regulation of protein kinase activity | 21 | -12.99505583 |
| GO Biological Processes | GO:0010332 | response to gamma radiation | 10 | -12.97731215 |
| GO Biological Processes | GO:1901568 | fatty acid derivative metabolic process | 14 | -12.97182999 |
| GO Biological Processes | GO:0001667 | ameboidal-type cell migration | 20 | -12.90274361 |
| GO Biological Processes | GO:0033674 | positive regulation of kinase activity | 22 | -12.89337077 |
| GO Biological Processes | GO:0030522 | intracellular receptor signaling pathway | 16 | -12.78339979 |
| GO Biological Processes | GO:0043410 | positive regulation of MAPK cascade | 21 | -12.76579669 |
| GO Biological Processes | GO:0051347 | positive regulation of transferase activity | 23 | -12.75838513 |
| GO Biological Processes | GO:0048145 | regulation of fibroblast proliferation | 11 | -12.72951943 |
| GO Biological Processes | GO:0042542 | response to hydrogen peroxide | 13 | -12.6767916 |
| GO Biological Processes | GO:0002064 | epithelial cell development | 15 | -12.67562328 |
| GO Biological Processes | GO:0048144 | fibroblast proliferation | 11 | -12.67027415 |
| GO Biological Processes | GO:0001676 | long-chain fatty acid metabolic process | 12 | -12.66073896 |
| GO Biological Processes | GO:0051384 | response to glucocorticoid | 13 | -12.6382859 |
| GO Biological Processes | GO:2000045 | regulation of G1/S transition of mitotic cell cycle | 14 | -12.63812966 |
| GO Biological Processes | GO:1903706 | regulation of hemopoiesis | 20 | -12.62460128 |
| GO Biological Processes | GO:0051345 | positive regulation of hydrolase activity | 24 | -12.60888372 |
| GO Biological Processes | GO:0010001 | glial cell differentiation | 15 | -12.56445755 |
| GO Biological Processes | GO:0010638 | positive regulation of organelle organization | 22 | -12.54734403 |
| GO Biological Processes | GO:1904018 | positive regulation of vasculature development | 15 | -12.42844359 |
| GO Biological Processes | GO:0050865 | regulation of cell activation | 22 | -12.37345722 |
| GO Biological Processes | GO:1903829 | positive regulation of cellular protein localization | 17 | -12.3519952 |
| GO Biological Processes | GO:0032103 | positive regulation of response to external stimulus | 17 | -12.33133732 |
| GO Biological Processes | GO:0000082 | G1/S transition of mitotic cell cycle | 16 | -12.32621846 |
| GO Biological Processes | GO:0045787 | positive regulation of cell cycle | 18 | -12.31562047 |
| GO Biological Processes | GO:0043062 | extracellular structure organization | 19 | -12.28245409 |
| GO Biological Processes | GO:0001890 | placenta development | 13 | -12.26824856 |
| GO Biological Processes | GO:1904951 | positive regulation of establishment of protein localization | 18 | -12.18739608 |
| GO Biological Processes | GO:0048589 | developmental growth | 22 | -12.15195851 |
| GO Biological Processes | GO:0071902 | positive regulation of protein serine/threonine kinase activity | 17 | -12.14840436 |
| GO Biological Processes | GO:0031099 | regeneration | 14 | -12.14319178 |
| GO Biological Processes | GO:0008637 | apoptotic mitochondrial changes | 12 | -12.12923056 |
| GO Biological Processes | GO:0045936 | negative regulation of phosphate metabolic process | 21 | -12.07676969 |
| GO Biological Processes | GO:0010563 | negative regulation of phosphorus metabolic process | 21 | -12.06308504 |
| GO Biological Processes | GO:0001503 | ossification | 18 | -12.00847262 |
| GO Biological Processes | GO:1902806 | regulation of cell cycle G1/S phase transition | 14 | -11.99731672 |
| GO Biological Processes | GO:0090068 | positive regulation of cell cycle process | 16 | -11.988256 |
| GO Biological Processes | GO:2001242 | regulation of intrinsic apoptotic signaling pathway | 13 | -11.95656354 |
| GO Biological Processes | GO:0071383 | cellular response to steroid hormone stimulus | 14 | -11.94004855 |
| GO Biological Processes | GO:0071456 | cellular response to hypoxia | 14 | -11.94004855 |
| GO Biological Processes | GO:1903827 | regulation of cellular protein localization | 20 | -11.89348266 |
| GO Biological Processes | GO:0070555 | response to interleukin-1 | 14 | -11.85526731 |
| GO Biological Processes | GO:0044843 | cell cycle G1/S phase transition | 16 | -11.81551299 |
| GO Biological Processes | GO:1901615 | organic hydroxy compound metabolic process | 20 | -11.77870749 |
| GO Biological Processes | GO:0043523 | regulation of neuron apoptotic process | 14 | -11.77179644 |
| GO Biological Processes | GO:0070661 | leukocyte proliferation | 16 | -11.73098256 |
| GO Biological Processes | GO:0046683 | response to organophosphorus | 12 | -11.72718503 |
| GO Biological Processes | GO:0006633 | fatty acid biosynthetic process | 13 | -11.72663335 |
| GO Biological Processes | GO:0098869 | cellular oxidant detoxification | 11 | -11.67185503 |
| GO Biological Processes | GO:0036294 | cellular response to decreased oxygen levels | 14 | -11.66247545 |
| GO Biological Processes | GO:0043281 | regulation of cysteine-type endopeptidase activity involved in apoptotic process | 14 | -11.66247545 |
| GO Biological Processes | GO:0038034 | signal transduction in absence of ligand | 10 | -11.64282782 |
| GO Biological Processes | GO:0043627 | response to estrogen | 10 | -11.64282782 |
| GO Biological Processes | GO:0097192 | extrinsic apoptotic signaling pathway in absence of ligand | 10 | -11.64282782 |
| GO Biological Processes | GO:0010634 | positive regulation of epithelial cell migration | 13 | -11.59976202 |
| GO Biological Processes | GO:0022411 | cellular component disassembly | 20 | -11.5271999 |
| GO Biological Processes | GO:0051235 | maintenance of location | 16 | -11.52477433 |
| GO Biological Processes | GO:0034762 | regulation of transmembrane transport | 20 | -11.4861577 |
| GO Biological Processes | GO:0048771 | tissue remodeling | 13 | -11.4455084 |
| GO Biological Processes | GO:0042136 | neurotransmitter biosynthetic process | 11 | -11.44245283 |
| GO Biological Processes | GO:0006809 | nitric oxide biosynthetic process | 10 | -11.40962014 |
| GO Biological Processes | GO:0008202 | steroid metabolic process | 16 | -11.3648308 |
| GO Biological Processes | GO:0007507 | heart development | 20 | -11.31108564 |
| GO Biological Processes | GO:0019932 | second-messenger-mediated signaling | 18 | -11.29088141 |
| GO Biological Processes | GO:0006091 | generation of precursor metabolites and energy | 19 | -11.28986205 |
| GO Biological Processes | GO:0055123 | digestive system development | 12 | -11.28556584 |
| GO Biological Processes | GO:0030193 | regulation of blood coagulation | 10 | -11.24305133 |
| GO Biological Processes | GO:0030198 | extracellular matrix organization | 17 | -11.23688922 |
| GO Biological Processes | GO:0003012 | muscle system process | 18 | -11.22803321 |
| GO Biological Processes | GO:0032868 | response to insulin | 15 | -11.22133859 |
| GO Biological Processes | GO:0007346 | regulation of mitotic cell cycle | 21 | -11.21313189 |
| GO Biological Processes | GO:0007249 | I-kappaB kinase/NF-kappaB signaling | 15 | -11.1996411 |
| GO Biological Processes | GO:1900046 | regulation of hemostasis | 10 | -11.18900648 |
| GO Biological Processes | GO:0001819 | positive regulation of cytokine production | 18 | -11.18130086 |
| GO Biological Processes | GO:0051046 | regulation of secretion | 22 | -11.1563537 |
| GO Biological Processes | GO:0007566 | embryo implantation | 9 | -11.14594 |
| GO Biological Processes | GO:0046209 | nitric oxide metabolic process | 10 | -11.13566929 |
| GO Biological Processes | GO:0090087 | regulation of peptide transport | 21 | -11.11669111 |
| GO Biological Processes | GO:0016999 | antibiotic metabolic process | 12 | -11.11303884 |
| GO Biological Processes | GO:0070663 | regulation of leukocyte proliferation | 14 | -11.09808864 |
| GO Biological Processes | GO:0002694 | regulation of leukocyte activation | 20 | -11.07584412 |
| GO Biological Processes | GO:1904645 | response to amyloid-beta | 9 | -11.07576465 |
| GO Biological Processes | GO:1902105 | regulation of leukocyte differentiation | 15 | -11.07119481 |
| GO Biological Processes | GO:0061061 | muscle structure development | 21 | -11.06896286 |
| GO Biological Processes | GO:2000116 | regulation of cysteine-type endopeptidase activity | 14 | -11.04962503 |
| GO Biological Processes | GO:0030336 | negative regulation of cell migration | 16 | -11.02011562 |
| GO Biological Processes | GO:0033559 | unsaturated fatty acid metabolic process | 11 | -11.01653138 |
| GO Biological Processes | GO:0009615 | response to virus | 16 | -11.00154752 |
| GO Biological Processes | GO:0023061 | signal release | 18 | -10.98267849 |
| GO Biological Processes | GO:2001057 | reactive nitrogen species metabolic process | 10 | -10.97972936 |
| GO Biological Processes | GO:1903530 | regulation of secretion by cell | 21 | -10.96274915 |
| GO Biological Processes | GO:1904019 | epithelial cell apoptotic process | 11 | -10.93607618 |
| GO Biological Processes | GO:0050818 | regulation of coagulation | 10 | -10.92905166 |
| GO Biological Processes | GO:0090559 | regulation of membrane permeability | 10 | -10.92905166 |
| GO Biological Processes | GO:1902895 | positive regulation of pri-miRNA transcription by RNA polymerase II | 8 | -10.90099718 |
| GO Biological Processes | GO:0008625 | extrinsic apoptotic signaling pathway via death domain receptors | 10 | -10.87899911 |
| GO Biological Processes | GO:0010632 | regulation of epithelial cell migration | 15 | -10.86348327 |
| GO Biological Processes | GO:0043122 | regulation of I-kappaB kinase/NF-kappaB signaling | 14 | -10.81374761 |
| GO Biological Processes | GO:0001558 | regulation of cell growth | 17 | -10.80304077 |
| GO Biological Processes | GO:0051101 | regulation of DNA binding | 11 | -10.77946189 |
| GO Biological Processes | GO:0045926 | negative regulation of growth | 14 | -10.76781118 |
| GO Biological Processes | GO:0001505 | regulation of neurotransmitter levels | 16 | -10.74763278 |
| GO Biological Processes | GO:2000146 | negative regulation of cell motility | 16 | -10.74763278 |
| GO Biological Processes | GO:1903708 | positive regulation of hemopoiesis | 13 | -10.73802057 |
| GO Biological Processes | GO:2000134 | negative regulation of G1/S transition of mitotic cell cycle | 11 | -10.70320684 |
| GO Biological Processes | GO:0045786 | negative regulation of cell cycle | 20 | -10.70104973 |
| GO Biological Processes | GO:0071229 | cellular response to acid chemical | 13 | -10.65947264 |
| GO Biological Processes | GO:0008631 | intrinsic apoptotic signaling pathway in response to oxidative stress | 8 | -10.63065349 |
| GO Biological Processes | GO:0045931 | positive regulation of mitotic cell cycle | 12 | -10.59994547 |
| GO Biological Processes | GO:0002685 | regulation of leukocyte migration | 13 | -10.58214723 |
| GO Biological Processes | GO:0051223 | regulation of protein transport | 20 | -10.58062237 |
| GO Biological Processes | GO:0045637 | regulation of myeloid cell differentiation | 14 | -10.54396602 |
| GO Biological Processes | GO:0060249 | anatomical structure homeostasis | 17 | -10.53530908 |
| GO Biological Processes | GO:1902532 | negative regulation of intracellular signal transduction | 19 | -10.49034603 |
| GO Biological Processes | GO:1902807 | negative regulation of cell cycle G1/S phase transition | 11 | -10.4821229 |
| GO Biological Processes | GO:0051249 | regulation of lymphocyte activation | 18 | -10.42187617 |
| GO Biological Processes | GO:0090303 | positive regulation of wound healing | 9 | -10.38058516 |
| GO Biological Processes | GO:0048565 | digestive tract development | 11 | -10.3756537 |
| GO Biological Processes | GO:0008585 | female gonad development | 10 | -10.36580056 |
| GO Biological Processes | GO:0034349 | glial cell apoptotic process | 6 | -10.34455311 |
| GO Biological Processes | GO:0090066 | regulation of anatomical structure size | 18 | -10.33990875 |
| GO Biological Processes | GO:0008406 | gonad development | 13 | -10.33278413 |
| GO Biological Processes | GO:0050863 | regulation of T cell activation | 15 | -10.28344348 |
| GO Biological Processes | GO:0045639 | positive regulation of myeloid cell differentiation | 10 | -10.27912887 |
| GO Biological Processes | GO:0009914 | hormone transport | 15 | -10.2651269 |
| GO Biological Processes | GO:0045137 | development of primary sexual characteristics | 13 | -10.18900297 |
| GO Biological Processes | GO:0071347 | cellular response to interleukin-1 | 12 | -10.16527428 |
| GO Biological Processes | GO:2001235 | positive regulation of apoptotic signaling pathway | 12 | -10.16527428 |
| GO Biological Processes | GO:0051271 | negative regulation of cellular component movement | 16 | -10.157848 |
| GO Biological Processes | GO:0006801 | superoxide metabolic process | 9 | -10.15709107 |
| GO Biological Processes | GO:0046545 | development of primary female sexual characteristics | 10 | -10.15253434 |
| GO Biological Processes | GO:0007610 | behavior | 19 | -10.14292946 |
| GO Biological Processes | GO:0046651 | lymphocyte proliferation | 14 | -10.12315825 |
| GO Biological Processes | GO:0052547 | regulation of peptidase activity | 17 | -10.10456397 |
| GO Biological Processes | GO:0032943 | mononuclear cell proliferation | 14 | -10.06288155 |
| GO Biological Processes | GO:0007050 | cell cycle arrest | 13 | -10.04931456 |
| GO Biological Processes | GO:0046902 | regulation of mitochondrial membrane permeability | 9 | -9.997972305 |
| GO Biological Processes | GO:0040013 | negative regulation of locomotion | 16 | -9.968908862 |
| GO Biological Processes | GO:0060627 | regulation of vesicle-mediated transport | 18 | -9.958427009 |
| GO Biological Processes | GO:0031649 | heat generation | 6 | -9.954706817 |
| GO Biological Processes | GO:0035265 | organ growth | 12 | -9.94913043 |
| GO Biological Processes | GO:0044057 | regulation of system process | 19 | -9.940896355 |
| GO Biological Processes | GO:0043269 | regulation of ion transport | 20 | -9.900714092 |
| GO Biological Processes | GO:0010821 | regulation of mitochondrion organization | 12 | -9.844752433 |
| GO Biological Processes | GO:1905475 | regulation of protein localization to membrane | 12 | -9.844752433 |
| GO Biological Processes | GO:0042445 | hormone metabolic process | 13 | -9.825027384 |
| GO Biological Processes | GO:0072594 | establishment of protein localization to organelle | 18 | -9.794859298 |
| GO Biological Processes | GO:0031098 | stress-activated protein kinase signaling cascade | 14 | -9.771654018 |
| GO Biological Processes | GO:0016049 | cell growth | 17 | -9.770406387 |
| GO Biological Processes | GO:0031400 | negative regulation of protein modification process | 19 | -9.767857068 |
| GO Biological Processes | GO:0045807 | positive regulation of endocytosis | 11 | -9.756162659 |
| GO Biological Processes | GO:1903036 | positive regulation of response to wounding | 9 | -9.699114113 |
| GO Biological Processes | GO:0042133 | neurotransmitter metabolic process | 11 | -9.695859997 |
| GO Biological Processes | GO:0045907 | positive regulation of vasoconstriction | 7 | -9.693808457 |
| GO Biological Processes | GO:0018107 | peptidyl-threonine phosphorylation | 10 | -9.682834422 |
| GO Biological Processes | GO:0019216 | regulation of lipid metabolic process | 16 | -9.682141806 |
| GO Biological Processes | GO:0008610 | lipid biosynthetic process | 20 | -9.637067678 |
| GO Biological Processes | GO:0060749 | mammary gland alveolus development | 6 | -9.61713491 |
| GO Biological Processes | GO:0061377 | mammary gland lobule development | 6 | -9.61713491 |
| GO Biological Processes | GO:0046660 | female sex differentiation | 10 | -9.609676207 |
| GO Biological Processes | GO:0030168 | platelet activation | 11 | -9.606972783 |
| GO Biological Processes | GO:1901030 | positive regulation of mitochondrial outer membrane permeabilization involved in apoptotic signaling pathway | 7 | -9.598583988 |
| GO Biological Processes | GO:0042743 | hydrogen peroxide metabolic process | 8 | -9.597711153 |
| GO Biological Processes | GO:0052548 | regulation of endopeptidase activity | 16 | -9.594554683 |
| GO Biological Processes | GO:0090257 | regulation of muscle system process | 13 | -9.58971712 |
| GO Biological Processes | GO:0060326 | cell chemotaxis | 14 | -9.586321046 |
| GO Biological Processes | GO:0051092 | positive regulation of NF-kappaB transcription factor activity | 11 | -9.57775066 |
| GO Biological Processes | GO:0010822 | positive regulation of mitochondrion organization | 10 | -9.573597889 |
| GO Biological Processes | GO:0030072 | peptide hormone secretion | 13 | -9.568882721 |
| GO Biological Processes | GO:0099536 | synaptic signaling | 20 | -9.554932039 |
| GO Biological Processes | GO:1902107 | positive regulation of leukocyte differentiation | 11 | -9.548727639 |
| GO Biological Processes | GO:0002763 | positive regulation of myeloid leukocyte differentiation | 8 | -9.534951668 |
| GO Biological Processes | GO:1904705 | regulation of vascular smooth muscle cell proliferation | 9 | -9.512683323 |
| GO Biological Processes | GO:1990874 | vascular smooth muscle cell proliferation | 9 | -9.512683323 |
| GO Biological Processes | GO:0006066 | alcohol metabolic process | 15 | -9.483367362 |
| GO Biological Processes | GO:0045862 | positive regulation of proteolysis | 15 | -9.483367362 |
| GO Biological Processes | GO:0006636 | unsaturated fatty acid biosynthetic process | 8 | -9.473368304 |
| GO Biological Processes | GO:0006109 | regulation of carbohydrate metabolic process | 12 | -9.473285164 |
| GO Biological Processes | GO:0002761 | regulation of myeloid leukocyte differentiation | 10 | -9.467297725 |
| GO Biological Processes | GO:0046879 | hormone secretion | 14 | -9.425111106 |
| GO Biological Processes | GO:0097755 | positive regulation of blood vessel diameter | 8 | -9.412918836 |
| GO Biological Processes | GO:0030073 | insulin secretion | 12 | -9.402499528 |
| GO Biological Processes | GO:0090276 | regulation of peptide hormone secretion | 12 | -9.402499528 |
| GO Biological Processes | GO:0050870 | positive regulation of T cell activation | 12 | -9.379148198 |
| GO Biological Processes | GO:0009408 | response to heat | 11 | -9.37863132 |
| GO Biological Processes | GO:0018210 | peptidyl-threonine modification | 10 | -9.363786824 |
| GO Biological Processes | GO:0050804 | modulation of chemical synaptic transmission | 16 | -9.353642161 |
| GO Biological Processes | GO:0015850 | organic hydroxy compound transport | 13 | -9.345471756 |
| GO Biological Processes | GO:0099177 | regulation of trans-synaptic signaling | 16 | -9.339792614 |
| GO Biological Processes | GO:1905477 | positive regulation of protein localization to membrane | 10 | -9.329879176 |
| GO Biological Processes | GO:1905952 | regulation of lipid localization | 11 | -9.296075169 |
| GO Biological Processes | GO:1900407 | regulation of cellular response to oxidative stress | 9 | -9.292363767 |
| GO Biological Processes | GO:0050670 | regulation of lymphocyte proliferation | 12 | -9.264168823 |
| GO Biological Processes | GO:0001655 | urogenital system development | 14 | -9.251823978 |
| GO Biological Processes | GO:0032147 | activation of protein kinase activity | 14 | -9.251823978 |
| GO Biological Processes | GO:0014909 | smooth muscle cell migration | 9 | -9.249866217 |
| GO Biological Processes | GO:0030856 | regulation of epithelial cell differentiation | 11 | -9.241919755 |
| GO Biological Processes | GO:0031571 | mitotic G1 DNA damage checkpoint | 8 | -9.237984207 |
| GO Biological Processes | GO:0071375 | cellular response to peptide hormone stimulus | 14 | -9.234819126 |
| GO Biological Processes | GO:0046883 | regulation of hormone secretion | 13 | -9.227865624 |
| GO Biological Processes | GO:0010959 | regulation of metal ion transport | 15 | -9.221163656 |
| GO Biological Processes | GO:0032944 | regulation of mononuclear cell proliferation | 12 | -9.218985789 |
| GO Biological Processes | GO:0001659 | temperature homeostasis | 11 | -9.188450323 |
| GO Biological Processes | GO:0055093 | response to hyperoxia | 6 | -9.182795995 |
| GO Biological Processes | GO:0044783 | G1 DNA damage checkpoint | 8 | -9.181690571 |
| GO Biological Processes | GO:0044819 | mitotic G1/S transition checkpoint | 8 | -9.181690571 |
| GO Biological Processes | GO:0034504 | protein localization to nucleus | 13 | -9.17013273 |
| GO Biological Processes | GO:0016572 | histone phosphorylation | 7 | -9.164143359 |
| GO Biological Processes | GO:0015980 | energy derivation by oxidation of organic compounds | 13 | -9.151043071 |
| GO Biological Processes | GO:0032768 | regulation of monooxygenase activity | 8 | -9.126350349 |
| GO Biological Processes | GO:1903035 | negative regulation of response to wounding | 9 | -9.125298391 |
| GO Biological Processes | GO:0007548 | sex differentiation | 13 | -9.094230098 |
| GO Biological Processes | GO:1902042 | negative regulation of extrinsic apoptotic signaling pathway via death domain receptors | 7 | -9.084523644 |
| GO Biological Processes | GO:0045930 | negative regulation of mitotic cell cycle | 14 | -9.084318551 |
| GO Biological Processes | GO:0002696 | positive regulation of leukocyte activation | 15 | -9.07281772 |
| GO Biological Processes | GO:0048469 | cell maturation | 11 | -9.057673264 |
| GO Biological Processes | GO:1901522 | positive regulation of transcription from RNA polymerase II promoter involved in cellular response to chemical stimulus | 6 | -9.053181507 |
| GO Biological Processes | GO:0036473 | cell death in response to oxidative stress | 9 | -9.044585334 |
| GO Biological Processes | GO:0050796 | regulation of insulin secretion | 11 | -9.006481168 |
| GO Biological Processes | GO:0005975 | carbohydrate metabolic process | 18 | -9.005181002 |
| GO Biological Processes | GO:0001894 | tissue homeostasis | 12 | -8.999648687 |
| GO Biological Processes | GO:0045055 | regulated exocytosis | 20 | -8.99859515 |
| GO Biological Processes | GO:0072511 | divalent inorganic cation transport | 16 | -8.991462922 |
| GO Biological Processes | GO:0043542 | endothelial cell migration | 13 | -8.982607113 |
| GO Biological Processes | GO:0045428 | regulation of nitric oxide biosynthetic process | 8 | -8.965748595 |
| GO Biological Processes | GO:0051403 | stress-activated MAPK cascade | 13 | -8.964256751 |
| GO Biological Processes | GO:0030595 | leukocyte chemotaxis | 12 | -8.957042305 |
| GO Biological Processes | GO:0042326 | negative regulation of phosphorylation | 16 | -8.952666472 |
| GO Biological Processes | GO:0009743 | response to carbohydrate | 12 | -8.935890723 |
| GO Biological Processes | GO:0046688 | response to copper ion | 7 | -8.931546733 |
| GO Biological Processes | GO:0019748 | secondary metabolic process | 8 | -8.913927488 |
| GO Biological Processes | GO:0031334 | positive regulation of protein complex assembly | 13 | -8.909629968 |
| GO Biological Processes | GO:0007268 | chemical synaptic transmission | 19 | -8.889090147 |
| GO Biological Processes | GO:0098916 | anterograde trans-synaptic signaling | 19 | -8.889090147 |
| GO Biological Processes | GO:1902882 | regulation of response to oxidative stress | 9 | -8.888407216 |
| GO Biological Processes | GO:0010876 | lipid localization | 15 | -8.871971958 |
| GO Biological Processes | GO:0050867 | positive regulation of cell activation | 15 | -8.857918733 |
| GO Biological Processes | GO:0034976 | response to endoplasmic reticulum stress | 13 | -8.85562927 |
| GO Biological Processes | GO:0008630 | intrinsic apoptotic signaling pathway in response to DNA damage | 9 | -8.850403788 |
| GO Biological Processes | GO:0044262 | cellular carbohydrate metabolic process | 13 | -8.837765913 |
| GO Biological Processes | GO:0051251 | positive regulation of lymphocyte activation | 14 | -8.827496442 |
| GO Biological Processes | GO:0043279 | response to alkaloid | 9 | -8.81279998 |
| GO Biological Processes | GO:0061180 | mammary gland epithelium development | 8 | -8.812699538 |
| GO Biological Processes | GO:0099537 | trans-synaptic signaling | 19 | -8.810730115 |
| GO Biological Processes | GO:0051146 | striated muscle cell differentiation | 13 | -8.802241679 |
| GO Biological Processes | GO:0043254 | regulation of protein complex assembly | 16 | -8.787691634 |
| GO Biological Processes | GO:0035094 | response to nicotine | 7 | -8.786236937 |
| GO Biological Processes | GO:0045429 | positive regulation of nitric oxide biosynthetic process | 7 | -8.786236937 |
| GO Biological Processes | GO:1901028 | regulation of mitochondrial outer membrane permeabilization involved in apoptotic signaling pathway | 7 | -8.786236937 |
| GO Biological Processes | GO:0033135 | regulation of peptidyl-serine phosphorylation | 10 | -8.76497322 |
| GO Biological Processes | GO:0006935 | chemotaxis | 18 | -8.739551928 |
| GO Biological Processes | GO:0014812 | muscle cell migration | 9 | -8.738759584 |
| GO Biological Processes | GO:0042330 | taxis | 18 | -8.718818333 |
| GO Biological Processes | GO:1904407 | positive regulation of nitric oxide metabolic process | 7 | -8.716228669 |
| GO Biological Processes | GO:0051222 | positive regulation of protein transport | 14 | -8.703916776 |
| GO Biological Processes | GO:0032091 | negative regulation of protein binding | 9 | -8.702307756 |
| GO Biological Processes | GO:1903426 | regulation of reactive oxygen species biosynthetic process | 9 | -8.702307756 |
| GO Biological Processes | GO:0051051 | negative regulation of transport | 16 | -8.688624986 |
| GO Biological Processes | GO:0030225 | macrophage differentiation | 7 | -8.647875704 |
| GO Biological Processes | GO:0098771 | inorganic ion homeostasis | 19 | -8.647550558 |
| GO Biological Processes | GO:0042098 | T cell proliferation | 11 | -8.641312713 |
| GO Biological Processes | GO:0033138 | positive regulation of peptidyl-serine phosphorylation | 9 | -8.630504393 |
| GO Biological Processes | GO:0006606 | protein import into nucleus | 10 | -8.621595693 |
| GO Biological Processes | GO:0070665 | positive regulation of leukocyte proliferation | 10 | -8.621595693 |
| GO Biological Processes | GO:0031100 | animal organ regeneration | 8 | -8.619255272 |
| GO Biological Processes | GO:1902175 | regulation of oxidative stress-induced intrinsic apoptotic signaling pathway | 6 | -8.592625414 |
| GO Biological Processes | GO:0006869 | lipid transport | 14 | -8.583368087 |
| GO Biological Processes | GO:0009409 | response to cold | 7 | -8.581103172 |
| GO Biological Processes | GO:1903524 | positive regulation of blood circulation | 8 | -8.572648511 |
| GO Biological Processes | GO:0018108 | peptidyl-tyrosine phosphorylation | 14 | -8.553688976 |
| GO Biological Processes | GO:0051924 | regulation of calcium ion transport | 12 | -8.552051684 |
| GO Biological Processes | GO:0001933 | negative regulation of protein phosphorylation | 15 | -8.544838176 |
| GO Biological Processes | GO:0019318 | hexose metabolic process | 12 | -8.53274485 |
| GO Biological Processes | GO:0150076 | neuroinflammatory response | 8 | -8.526704762 |
| GO Biological Processes | GO:0006873 | cellular ion homeostasis | 18 | -8.525490437 |
| GO Biological Processes | GO:0018212 | peptidyl-tyrosine modification | 14 | -8.509505804 |
| GO Biological Processes | GO:0009755 | hormone-mediated signaling pathway | 11 | -8.503782457 |
| GO Biological Processes | GO:0001938 | positive regulation of endothelial cell proliferation | 9 | -8.491107555 |
| GO Biological Processes | GO:0032963 | collagen metabolic process | 9 | -8.491107555 |
| GO Biological Processes | GO:0010575 | positive regulation of vascular endothelial growth factor production | 6 | -8.489554073 |
| GO Biological Processes | GO:0030194 | positive regulation of blood coagulation | 6 | -8.489554073 |
| GO Biological Processes | GO:1900048 | positive regulation of hemostasis | 6 | -8.489554073 |
| GO Biological Processes | GO:0042310 | vasoconstriction | 8 | -8.481406052 |
| GO Biological Processes | GO:0071260 | cellular response to mechanical stimulus | 8 | -8.436735129 |
| GO Biological Processes | GO:0009896 | positive regulation of catabolic process | 15 | -8.427356022 |
| GO Biological Processes | GO:0042594 | response to starvation | 11 | -8.414577104 |
| GO Biological Processes | GO:0031349 | positive regulation of defense response | 16 | -8.413636535 |
| GO Biological Processes | GO:0051129 | negative regulation of cellular component organization | 19 | -8.406362271 |
| GO Biological Processes | GO:0043536 | positive regulation of blood vessel endothelial cell migration | 8 | -8.392675424 |
| GO Biological Processes | GO:0036296 | response to increased oxygen levels | 6 | -8.390547836 |
| GO Biological Processes | GO:0050820 | positive regulation of coagulation | 6 | -8.390547836 |
| GO Biological Processes | GO:0046620 | regulation of organ growth | 9 | -8.390043017 |
| GO Biological Processes | GO:0048146 | positive regulation of fibroblast proliferation | 7 | -8.389591553 |
| GO Biological Processes | GO:0050730 | regulation of peptidyl-tyrosine phosphorylation | 12 | -8.362704336 |
| GO Biological Processes | GO:0061045 | negative regulation of wound healing | 8 | -8.349211018 |
| GO Biological Processes | GO:0031331 | positive regulation of cellular catabolic process | 14 | -8.336678909 |
| GO Biological Processes | GO:0050999 | regulation of nitric-oxide synthase activity | 7 | -8.328485354 |
| GO Biological Processes | GO:0051205 | protein insertion into membrane | 7 | -8.328485354 |
| GO Biological Processes | GO:0034764 | positive regulation of transmembrane transport | 11 | -8.327265512 |
| GO Biological Processes | GO:0007259 | JAK-STAT cascade | 10 | -8.297816553 |
| GO Biological Processes | GO:0043467 | regulation of generation of precursor metabolites and energy | 10 | -8.297816553 |
| GO Biological Processes | GO:0001844 | protein insertion into mitochondrial membrane involved in apoptotic signaling pathway | 6 | -8.295301664 |
| GO Biological Processes | GO:1901215 | negative regulation of neuron death | 11 | -8.284296818 |
| GO Biological Processes | GO:0009267 | cellular response to starvation | 10 | -8.272030392 |
| GO Biological Processes | GO:0097366 | response to bronchodilator | 7 | -8.268651893 |
| GO Biological Processes | GO:0010564 | regulation of cell cycle process | 19 | -8.262719878 |
| GO Biological Processes | GO:0006006 | glucose metabolic process | 11 | -8.24177443 |
| GO Biological Processes | GO:0043535 | regulation of blood vessel endothelial cell migration | 10 | -8.220975024 |
| GO Biological Processes | GO:0030857 | negative regulation of epithelial cell differentiation | 7 | -8.210040512 |
| GO Biological Processes | GO:0007093 | mitotic cell cycle checkpoint | 10 | -8.195701528 |
| GO Biological Processes | GO:0061419 | positive regulation of transcription from RNA polymerase II promoter in response to hypoxia | 4 | -8.166219565 |
| GO Biological Processes | GO:0097345 | mitochondrial outer membrane permeabilization | 7 | -8.152603527 |
| GO Biological Processes | GO:0070838 | divalent metal ion transport | 15 | -8.151149481 |
| GO Biological Processes | GO:0051962 | positive regulation of nervous system development | 16 | -8.141173402 |
| GO Biological Processes | GO:0014910 | regulation of smooth muscle cell migration | 8 | -8.140302573 |
| GO Biological Processes | GO:0097756 | negative regulation of blood vessel diameter | 8 | -8.140302573 |
| GO Biological Processes | GO:0050708 | regulation of protein secretion | 14 | -8.128985482 |
| GO Biological Processes | GO:0006977 | DNA damage response, signal transduction by p53 class mediator resulting in cell cycle arrest | 7 | -8.096295993 |
| GO Biological Processes | GO:0043491 | protein kinase B signaling | 12 | -8.093258264 |
| GO Biological Processes | GO:0002009 | morphogenesis of an epithelium | 16 | -8.086152828 |
| GO Biological Processes | GO:0051170 | import into nucleus | 10 | -8.07179363 |
| GO Biological Processes | GO:0097696 | STAT cascade | 10 | -8.047490151 |
| GO Biological Processes | GO:0001541 | ovarian follicle development | 7 | -8.041075499 |
| GO Biological Processes | GO:0072431 | signal transduction involved in mitotic G1 DNA damage checkpoint | 7 | -8.041075499 |
| GO Biological Processes | GO:1902400 | intracellular signal transduction involved in G1 DNA damage checkpoint | 7 | -8.041075499 |
| GO Biological Processes | GO:0050769 | positive regulation of neurogenesis | 15 | -8.030450565 |
| GO Biological Processes | GO:0010039 | response to iron ion | 6 | -8.029544192 |
| GO Biological Processes | GO:0015718 | monocarboxylic acid transport | 10 | -8.0233416 |
| GO Biological Processes | GO:0033209 | tumor necrosis factor-mediated signaling pathway | 10 | -8.0233416 |
| GO Biological Processes | GO:0043405 | regulation of MAP kinase activity | 13 | -7.992416718 |
| GO Biological Processes | GO:0001822 | kidney development | 12 | -7.990006886 |
| GO Biological Processes | GO:0043270 | positive regulation of ion transport | 12 | -7.990006886 |
| GO Biological Processes | GO:0019369 | arachidonic acid metabolic process | 7 | -7.986901984 |
| GO Biological Processes | GO:0010506 | regulation of autophagy | 13 | -7.977592685 |
| GO Biological Processes | GO:0032869 | cellular response to insulin stimulus | 11 | -7.955928919 |
| GO Biological Processes | GO:0051204 | protein insertion into mitochondrial membrane | 6 | -7.94688681 |
| GO Biological Processes | GO:0010720 | positive regulation of cell development | 16 | -7.945770075 |
| GO Biological Processes | GO:0021700 | developmental maturation | 12 | -7.939295514 |
| GO Biological Processes | GO:0055080 | cation homeostasis | 18 | -7.936901724 |
| GO Biological Processes | GO:0050920 | regulation of chemotaxis | 11 | -7.936261761 |
| GO Biological Processes | GO:0031663 | lipopolysaccharide-mediated signaling pathway | 7 | -7.933737563 |
| GO Biological Processes | GO:0043525 | positive regulation of neuron apoptotic process | 7 | -7.933737563 |
| GO Biological Processes | GO:0072413 | signal transduction involved in mitotic cell cycle checkpoint | 7 | -7.933737563 |
| GO Biological Processes | GO:1902402 | signal transduction involved in mitotic DNA damage checkpoint | 7 | -7.933737563 |
| GO Biological Processes | GO:1902403 | signal transduction involved in mitotic DNA integrity checkpoint | 7 | -7.933737563 |
| GO Biological Processes | GO:0050680 | negative regulation of epithelial cell proliferation | 10 | -7.928260442 |
| GO Biological Processes | GO:0072657 | protein localization to membrane | 17 | -7.926574449 |
| GO Biological Processes | GO:0030100 | regulation of endocytosis | 12 | -7.922523965 |
| GO Biological Processes | GO:0001836 | release of cytochrome c from mitochondria | 7 | -7.881546374 |
| GO Biological Processes | GO:0019229 | regulation of vasoconstriction | 7 | -7.881546374 |
| GO Biological Processes | GO:1903428 | positive regulation of reactive oxygen species biosynthetic process | 7 | -7.881546374 |
| GO Biological Processes | GO:0006816 | calcium ion transport | 14 | -7.852569813 |
| GO Biological Processes | GO:0072001 | renal system development | 12 | -7.83963721 |
| GO Biological Processes | GO:1902041 | regulation of extrinsic apoptotic signaling pathway via death domain receptors | 7 | -7.830294436 |
| GO Biological Processes | GO:1902110 | positive regulation of mitochondrial membrane permeability involved in apoptotic process | 7 | -7.830294436 |
| GO Biological Processes | GO:0030003 | cellular cation homeostasis | 17 | -7.830118558 |
| GO Biological Processes | GO:0045444 | fat cell differentiation | 11 | -7.801212633 |
| GO Biological Processes | GO:0051591 | response to cAMP | 8 | -7.795591196 |
| GO Biological Processes | GO:0048729 | tissue morphogenesis | 17 | -7.792020485 |
| GO Biological Processes | GO:0005996 | monosaccharide metabolic process | 12 | -7.790665805 |
| GO Biological Processes | GO:0001959 | regulation of cytokine-mediated signaling pathway | 10 | -7.789988794 |
| GO Biological Processes | GO:0001893 | maternal placenta development | 6 | -7.789362272 |
| GO Biological Processes | GO:0034405 | response to fluid shear stress | 6 | -7.789362272 |
| GO Biological Processes | GO:1901099 | negative regulation of signal transduction in absence of ligand | 6 | -7.789362272 |
| GO Biological Processes | GO:2001240 | negative regulation of extrinsic apoptotic signaling pathway in absence of ligand | 6 | -7.789362272 |
| GO Biological Processes | GO:0050671 | positive regulation of lymphocyte proliferation | 9 | -7.781659199 |
| GO Biological Processes | GO:0051348 | negative regulation of transferase activity | 12 | -7.774466157 |
| GO Biological Processes | GO:0035994 | response to muscle stretch | 5 | -7.770682277 |
| GO Biological Processes | GO:0032946 | positive regulation of mononuclear cell proliferation | 9 | -7.753853931 |
| GO Biological Processes | GO:0002791 | regulation of peptide secretion | 14 | -7.751366697 |
| GO Biological Processes | GO:1902686 | mitochondrial outer membrane permeabilization involved in programmed cell death | 7 | -7.730481022 |
| GO Biological Processes | GO:0008217 | regulation of blood pressure | 10 | -7.700557363 |
| GO Biological Processes | GO:0030098 | lymphocyte differentiation | 13 | -7.690895646 |
| GO Biological Processes | GO:1904035 | regulation of epithelial cell apoptotic process | 8 | -7.688528969 |
| GO Biological Processes | GO:2001243 | negative regulation of intrinsic apoptotic signaling pathway | 8 | -7.688528969 |
| GO Biological Processes | GO:0043123 | positive regulation of I-kappaB kinase/NF-kappaB signaling | 10 | -7.678529358 |
| GO Biological Processes | GO:0009306 | protein secretion | 15 | -7.662838672 |
| GO Biological Processes | GO:0010565 | regulation of cellular ketone metabolic process | 10 | -7.65663042 |
| GO Biological Processes | GO:0071478 | cellular response to radiation | 10 | -7.65663042 |
| GO Biological Processes | GO:0043401 | steroid hormone mediated signaling pathway | 9 | -7.644812312 |
| GO Biological Processes | GO:0032885 | regulation of polysaccharide biosynthetic process | 6 | -7.641215038 |
| GO Biological Processes | GO:0042391 | regulation of membrane potential | 14 | -7.640057013 |
| GO Biological Processes | GO:0030308 | negative regulation of cell growth | 10 | -7.634859151 |
| GO Biological Processes | GO:0043534 | blood vessel endothelial cell migration | 10 | -7.634859151 |
| GO Biological Processes | GO:0010573 | vascular endothelial growth factor production | 7 | -7.634058426 |
| GO Biological Processes | GO:0035794 | positive regulation of mitochondrial membrane permeability | 7 | -7.634058426 |
| GO Biological Processes | GO:1905953 | negative regulation of lipid localization | 7 | -7.634058426 |
| GO Biological Processes | GO:0002687 | positive regulation of leukocyte migration | 9 | -7.618080843 |
| GO Biological Processes | GO:0007006 | mitochondrial membrane organization | 9 | -7.618080843 |
| GO Biological Processes | GO:0044772 | mitotic cell cycle phase transition | 16 | -7.615710189 |
| GO Biological Processes | GO:1901361 | organic cyclic compound catabolic process | 18 | -7.604744837 |
| GO Biological Processes | GO:0046824 | positive regulation of nucleocytoplasmic transport | 7 | -7.587050362 |
| GO Biological Processes | GO:1902108 | regulation of mitochondrial membrane permeability involved in apoptotic process | 7 | -7.587050362 |
| GO Biological Processes | GO:0019217 | regulation of fatty acid metabolic process | 8 | -7.584975885 |
| GO Biological Processes | GO:0044773 | mitotic DNA damage checkpoint | 8 | -7.584975885 |
| GO Biological Processes | GO:1901990 | regulation of mitotic cell cycle phase transition | 14 | -7.579343179 |
| GO Biological Processes | GO:0051052 | regulation of DNA metabolic process | 14 | -7.543290668 |
| GO Biological Processes | GO:1905710 | positive regulation of membrane permeability | 7 | -7.540810613 |
| GO Biological Processes | GO:0050731 | positive regulation of peptidyl-tyrosine phosphorylation | 10 | -7.527870532 |
| GO Biological Processes | GO:1901991 | negative regulation of mitotic cell cycle phase transition | 11 | -7.526283195 |
| GO Biological Processes | GO:0060965 | negative regulation of gene silencing by miRNA | 5 | -7.516379165 |
| GO Biological Processes | GO:0060759 | regulation of response to cytokine stimulus | 10 | -7.506837186 |
| GO Biological Processes | GO:2000273 | positive regulation of signaling receptor activity | 6 | -7.501409372 |
| GO Biological Processes | GO:0048662 | negative regulation of smooth muscle cell proliferation | 7 | -7.450541574 |
| GO Biological Processes | GO:0071230 | cellular response to amino acid stimulus | 7 | -7.450541574 |
| GO Biological Processes | GO:0071236 | cellular response to antibiotic | 9 | -7.436552196 |
| GO Biological Processes | GO:0043112 | receptor metabolic process | 10 | -7.423876608 |
| GO Biological Processes | GO:0048872 | homeostasis of number of cells | 11 | -7.421416109 |
| GO Biological Processes | GO:0071156 | regulation of cell cycle arrest | 8 | -7.419615317 |
| GO Biological Processes | GO:0042698 | ovulation cycle | 7 | -7.406467719 |
| GO Biological Processes | GO:0042180 | cellular ketone metabolic process | 11 | -7.37000692 |
| GO Biological Processes | GO:0042692 | muscle cell differentiation | 13 | -7.369497227 |
| GO Biological Processes | GO:0044774 | mitotic DNA integrity checkpoint | 8 | -7.355836665 |
| GO Biological Processes | GO:0045834 | positive regulation of lipid metabolic process | 9 | -7.336975152 |
| GO Biological Processes | GO:0010888 | negative regulation of lipid storage | 5 | -7.289566772 |
| GO Biological Processes | GO:0042359 | vitamin D metabolic process | 5 | -7.289566772 |
| GO Biological Processes | GO:0017038 | protein import | 10 | -7.283030123 |
| GO Biological Processes | GO:0072401 | signal transduction involved in DNA integrity checkpoint | 7 | -7.278241851 |
| GO Biological Processes | GO:0072422 | signal transduction involved in DNA damage checkpoint | 7 | -7.278241851 |
| GO Biological Processes | GO:0034284 | response to monosaccharide | 10 | -7.263341664 |
| GO Biological Processes | GO:1904659 | glucose transmembrane transport | 8 | -7.262543538 |
| GO Biological Processes | GO:0043406 | positive regulation of MAP kinase activity | 11 | -7.252598547 |
| GO Biological Processes | GO:0002790 | peptide secretion | 15 | -7.24929553 |
| GO Biological Processes | GO:0051121 | hepoxilin metabolic process | 4 | -7.246676438 |
| GO Biological Processes | GO:0051122 | hepoxilin biosynthetic process | 4 | -7.246676438 |
| GO Biological Processes | GO:0032881 | regulation of polysaccharide metabolic process | 6 | -7.243462083 |
| GO Biological Processes | GO:0070371 | ERK1 and ERK2 cascade | 12 | -7.243380042 |
| GO Biological Processes | GO:0072395 | signal transduction involved in cell cycle checkpoint | 7 | -7.236768345 |
| GO Biological Processes | GO:1901988 | negative regulation of cell cycle phase transition | 11 | -7.203335982 |
| GO Biological Processes | GO:0043393 | regulation of protein binding | 10 | -7.185626928 |
| GO Biological Processes | GO:0044770 | cell cycle phase transition | 16 | -7.185257388 |
| GO Biological Processes | GO:0060149 | negative regulation of posttranscriptional gene silencing | 5 | -7.184741587 |
| GO Biological Processes | GO:0060967 | negative regulation of gene silencing by RNA | 5 | -7.184741587 |
| GO Biological Processes | GO:1903798 | regulation of production of miRNAs involved in gene silencing by miRNA | 5 | -7.184741587 |
| GO Biological Processes | GO:1904996 | positive regulation of leukocyte adhesion to vascular endothelial cell | 5 | -7.184741587 |
| GO Biological Processes | GO:0006775 | fat-soluble vitamin metabolic process | 6 | -7.182976744 |
| GO Biological Processes | GO:0034105 | positive regulation of tissue remodeling | 6 | -7.182976744 |
| GO Biological Processes | GO:0007517 | muscle organ development | 13 | -7.179903518 |
| GO Biological Processes | GO:0008645 | hexose transmembrane transport | 8 | -7.17196067 |
| GO Biological Processes | GO:0016101 | diterpenoid metabolic process | 8 | -7.17196067 |
| GO Biological Processes | GO:0044089 | positive regulation of cellular component biogenesis | 15 | -7.158717391 |
| GO Biological Processes | GO:0060537 | muscle tissue development | 13 | -7.14295289 |
| GO Biological Processes | GO:0043620 | regulation of DNA-templated transcription in response to stress | 8 | -7.142343185 |
| GO Biological Processes | GO:1901987 | regulation of cell cycle phase transition | 14 | -7.14223239 |
| GO Biological Processes | GO:0045927 | positive regulation of growth | 11 | -7.138603056 |
| GO Biological Processes | GO:0014015 | positive regulation of gliogenesis | 7 | -7.115908701 |
| GO Biological Processes | GO:0033555 | multicellular organismal response to stress | 7 | -7.115908701 |
| GO Biological Processes | GO:1903201 | regulation of oxidative stress-induced cell death | 7 | -7.115908701 |
| GO Biological Processes | GO:0015749 | monosaccharide transmembrane transport | 8 | -7.113005492 |
| GO Biological Processes | GO:0055065 | metal ion homeostasis | 16 | -7.094462522 |
| GO Biological Processes | GO:0036003 | positive regulation of transcription from RNA polymerase II promoter in response to stress | 5 | -7.084913964 |
| GO Biological Processes | GO:0043500 | muscle adaptation | 8 | -7.083942634 |
| GO Biological Processes | GO:0051341 | regulation of oxidoreductase activity | 8 | -7.083942634 |
| GO Biological Processes | GO:0042572 | retinol metabolic process | 6 | -7.066289779 |
| GO Biological Processes | GO:0070849 | response to epidermal growth factor | 6 | -7.066289779 |
| GO Biological Processes | GO:0007569 | cell aging | 8 | -7.055149782 |
| GO Biological Processes | GO:0034219 | carbohydrate transmembrane transport | 8 | -7.055149782 |
| GO Biological Processes | GO:0000075 | cell cycle checkpoint | 10 | -7.053475583 |
| GO Biological Processes | GO:0002274 | myeloid leukocyte activation | 16 | -7.031891025 |
| GO Biological Processes | GO:0001660 | fever generation | 4 | -7.02640602 |
| GO Biological Processes | GO:1904646 | cellular response to amyloid-beta | 6 | -7.009960141 |
| GO Biological Processes | GO:2001239 | regulation of extrinsic apoptotic signaling pathway in absence of ligand | 6 | -7.009960141 |
| GO Biological Processes | GO:0042129 | regulation of T cell proliferation | 9 | -7.009734757 |
| GO Biological Processes | GO:0071695 | anatomical structure maturation | 9 | -7.009734757 |
| GO Biological Processes | GO:0060557 | positive regulation of vitamin D biosynthetic process | 3 | -6.999343974 |
| GO Biological Processes | GO:0060559 | positive regulation of calcidiol 1-monooxygenase activity | 3 | -6.999343974 |
| GO Biological Processes | GO:0046697 | decidualization | 5 | -6.98963384 |
| GO Biological Processes | GO:0070920 | regulation of production of small RNA involved in gene silencing by RNA | 5 | -6.98963384 |
| GO Biological Processes | GO:0010906 | regulation of glucose metabolic process | 8 | -6.970344842 |
| GO Biological Processes | GO:0019915 | lipid storage | 7 | -6.962489822 |
| GO Biological Processes | GO:0071158 | positive regulation of cell cycle arrest | 7 | -6.962489822 |
| GO Biological Processes | GO:0097529 | myeloid leukocyte migration | 10 | -6.961933593 |
| GO Biological Processes | GO:0006692 | prostanoid metabolic process | 6 | -6.95489474 |
| GO Biological Processes | GO:0006693 | prostaglandin metabolic process | 6 | -6.95489474 |
| GO Biological Processes | GO:0007595 | lactation | 6 | -6.95489474 |
| GO Biological Processes | GO:0006875 | cellular metal ion homeostasis | 15 | -6.9538762 |
| GO Biological Processes | GO:0021782 | glial cell development | 8 | -6.942586188 |
| GO Biological Processes | GO:0042752 | regulation of circadian rhythm | 8 | -6.942586188 |
| GO Biological Processes | GO:0006913 | nucleocytoplasmic transport | 12 | -6.941180728 |
| GO Biological Processes | GO:0034644 | cellular response to UV | 7 | -6.92542019 |
| GO Biological Processes | GO:0043900 | regulation of multi-organism process | 13 | -6.91598225 |
| GO Biological Processes | GO:0045471 | response to ethanol | 8 | -6.915075206 |
| GO Biological Processes | GO:0051169 | nuclear transport | 12 | -6.901643127 |
| GO Biological Processes | GO:0050806 | positive regulation of synaptic transmission | 9 | -6.900134264 |
| GO Biological Processes | GO:0019430 | removal of superoxide radicals | 5 | -6.898509546 |
| GO Biological Processes | GO:1900739 | regulation of protein insertion into mitochondrial membrane involved in apoptotic signaling pathway | 5 | -6.898509546 |
| GO Biological Processes | GO:1900740 | positive regulation of protein insertion into mitochondrial membrane involved in apoptotic signaling pathway | 5 | -6.898509546 |
| GO Biological Processes | GO:0001937 | negative regulation of endothelial cell proliferation | 7 | -6.888838589 |
| GO Biological Processes | GO:0048708 | astrocyte differentiation | 7 | -6.888838589 |
| GO Biological Processes | GO:0002688 | regulation of leukocyte chemotaxis | 8 | -6.887807763 |
| GO Biological Processes | GO:0006721 | terpenoid metabolic process | 8 | -6.887807763 |
| GO Biological Processes | GO:0022612 | gland morphogenesis | 8 | -6.860779828 |
| GO Biological Processes | GO:0051099 | positive regulation of binding | 9 | -6.857275907 |
| GO Biological Processes | GO:0045913 | positive regulation of carbohydrate metabolic process | 7 | -6.852732871 |
| GO Biological Processes | GO:0006984 | ER-nucleus signaling pathway | 6 | -6.848343937 |
| GO Biological Processes | GO:0061028 | establishment of endothelial barrier | 6 | -6.848343937 |
| GO Biological Processes | GO:0034605 | cellular response to heat | 8 | -6.83398747 |
| GO Biological Processes | GO:0033197 | response to vitamin E | 4 | -6.831689513 |
| GO Biological Processes | GO:0046886 | positive regulation of hormone biosynthetic process | 4 | -6.831689513 |
| GO Biological Processes | GO:0001649 | osteoblast differentiation | 10 | -6.820115195 |
| GO Biological Processes | GO:0043154 | negative regulation of cysteine-type endopeptidase activity involved in apoptotic process | 7 | -6.817091333 |
| GO Biological Processes | GO:0071621 | granulocyte chemotaxis | 8 | -6.807426854 |
| GO Biological Processes | GO:0002764 | immune response-regulating signaling pathway | 16 | -6.806282166 |
| GO Biological Processes | GO:0006970 | response to osmotic stress | 7 | -6.7819027 |
| GO Biological Processes | GO:0042446 | hormone biosynthetic process | 7 | -6.7819027 |
| GO Biological Processes | GO:0014013 | regulation of gliogenesis | 8 | -6.781094237 |
| GO Biological Processes | GO:0010469 | regulation of signaling receptor activity | 9 | -6.773170619 |
| GO Biological Processes | GO:0051897 | positive regulation of protein kinase B signaling | 9 | -6.773170619 |
| GO Biological Processes | GO:0034763 | negative regulation of transmembrane transport | 8 | -6.754985967 |
| GO Biological Processes | GO:0061756 | leukocyte adhesion to vascular endothelial cell | 6 | -6.746244254 |
| GO Biological Processes | GO:0032770 | positive regulation of monooxygenase activity | 5 | -6.7273974 |
| GO Biological Processes | GO:0071450 | cellular response to oxygen radical | 5 | -6.7273974 |
| GO Biological Processes | GO:0071451 | cellular response to superoxide | 5 | -6.7273974 |
| GO Biological Processes | GO:1990776 | response to angiotensin | 5 | -6.7273974 |
| GO Biological Processes | GO:0050890 | cognition | 11 | -6.713431123 |
| GO Biological Processes | GO:0010594 | regulation of endothelial cell migration | 10 | -6.70044644 |
| GO Biological Processes | GO:0006749 | glutathione metabolic process | 6 | -6.696753521 |
| GO Biological Processes | GO:0010524 | positive regulation of calcium ion transport into cytosol | 6 | -6.696753521 |
| GO Biological Processes | GO:0032872 | regulation of stress-activated MAPK cascade | 10 | -6.683672435 |
| GO Biological Processes | GO:0007077 | mitotic nuclear envelope disassembly | 4 | -6.657176197 |
| GO Biological Processes | GO:0042178 | xenobiotic catabolic process | 4 | -6.657176197 |
| GO Biological Processes | GO:0042368 | vitamin D biosynthetic process | 4 | -6.657176197 |
| GO Biological Processes | GO:0045080 | positive regulation of chemokine biosynthetic process | 4 | -6.657176197 |
| GO Biological Processes | GO:0010595 | positive regulation of endothelial cell migration | 8 | -6.652726299 |
| GO Biological Processes | GO:0042770 | signal transduction in response to DNA damage | 8 | -6.652726299 |
| GO Biological Processes | GO:0002931 | response to ischemia | 6 | -6.648248615 |
| GO Biological Processes | GO:0032800 | receptor biosynthetic process | 5 | -6.646840016 |
| GO Biological Processes | GO:0048568 | embryonic organ development | 13 | -6.645467995 |
| GO Biological Processes | GO:0001892 | embryonic placenta development | 7 | -6.645465543 |
| GO Biological Processes | GO:0070542 | response to fatty acid | 7 | -6.645465543 |
| GO Biological Processes | GO:0051054 | positive regulation of DNA metabolic process | 10 | -6.63381778 |
| GO Biological Processes | GO:0070302 | regulation of stress-activated protein kinase signaling cascade | 10 | -6.63381778 |
| GO Biological Processes | GO:0032368 | regulation of lipid transport | 8 | -6.627687932 |
| GO Biological Processes | GO:0034754 | cellular hormone metabolic process | 8 | -6.627687932 |
| GO Biological Processes | GO:0048639 | positive regulation of developmental growth | 9 | -6.591352336 |
| GO Biological Processes | GO:0034103 | regulation of tissue remodeling | 7 | -6.579699354 |
| GO Biological Processes | GO:0002366 | leukocyte activation involved in immune response | 16 | -6.574604316 |
| GO Biological Processes | GO:0000303 | response to superoxide | 5 | -6.569288013 |
| GO Biological Processes | GO:0048147 | negative regulation of fibroblast proliferation | 5 | -6.569288013 |
| GO Biological Processes | GO:0071480 | cellular response to gamma radiation | 5 | -6.569288013 |
| GO Biological Processes | GO:0043280 | positive regulation of cysteine-type endopeptidase activity involved in apoptotic process | 8 | -6.553785523 |
| GO Biological Processes | GO:0001701 | in utero embryonic development | 12 | -6.550121329 |
| GO Biological Processes | GO:2000117 | negative regulation of cysteine-type endopeptidase activity | 7 | -6.54739718 |
| GO Biological Processes | GO:0002263 | cell activation involved in immune response | 16 | -6.542410644 |
| GO Biological Processes | GO:1901992 | positive regulation of mitotic cell cycle phase transition | 7 | -6.515470843 |
| GO Biological Processes | GO:0046700 | heterocycle catabolic process | 16 | -6.502475137 |
| GO Biological Processes | GO:0010623 | programmed cell death involved in cell development | 4 | -6.499053103 |
| GO Biological Processes | GO:0000305 | response to oxygen radical | 5 | -6.49452878 |
| GO Biological Processes | GO:0071549 | cellular response to dexamethasone stimulus | 5 | -6.49452878 |
| GO Biological Processes | GO:0014706 | striated muscle tissue development | 12 | -6.490464291 |
| GO Biological Processes | GO:0097194 | execution phase of apoptosis | 7 | -6.483912116 |
| GO Biological Processes | GO:0003158 | endothelium development | 8 | -6.481639278 |
| GO Biological Processes | GO:0072655 | establishment of protein localization to mitochondrion | 8 | -6.481639278 |
| GO Biological Processes | GO:0030217 | T cell differentiation | 10 | -6.472506001 |
| GO Biological Processes | GO:0051896 | regulation of protein kinase B signaling | 10 | -6.472506001 |
| GO Biological Processes | GO:0046456 | icosanoid biosynthetic process | 6 | -6.463371062 |
| GO Biological Processes | GO:0071385 | cellular response to glucocorticoid stimulus | 6 | -6.463371062 |
| GO Biological Processes | GO:0008584 | male gonad development | 8 | -6.457967431 |
| GO Biological Processes | GO:0051188 | cofactor biosynthetic process | 11 | -6.449688582 |
| GO Biological Processes | GO:0046546 | development of primary male sexual characteristics | 8 | -6.434479472 |
| GO Biological Processes | GO:0010165 | response to X-ray | 5 | -6.422371549 |
| GO Biological Processes | GO:0043388 | positive regulation of DNA binding | 6 | -6.419274838 |
| GO Biological Processes | GO:0045598 | regulation of fat cell differentiation | 8 | -6.411172752 |
| GO Biological Processes | GO:0046425 | regulation of JAK-STAT cascade | 8 | -6.411172752 |
| GO Biological Processes | GO:0042176 | regulation of protein catabolic process | 12 | -6.408420669 |
| GO Biological Processes | GO:0043009 | chordate embryonic development | 15 | -6.405479375 |
| GO Biological Processes | GO:0009895 | negative regulation of catabolic process | 11 | -6.396438326 |
| GO Biological Processes | GO:0007005 | mitochondrion organization | 14 | -6.396108643 |
| GO Biological Processes | GO:0006720 | isoprenoid metabolic process | 8 | -6.38804468 |
| GO Biological Processes | GO:0050921 | positive regulation of chemotaxis | 8 | -6.38804468 |
| GO Biological Processes | GO:0070585 | protein localization to mitochondrion | 8 | -6.38804468 |
| GO Biological Processes | GO:0010574 | regulation of vascular endothelial growth factor production | 6 | -6.375968872 |
| GO Biological Processes | GO:0007611 | learning or memory | 10 | -6.363809809 |
| GO Biological Processes | GO:0043255 | regulation of carbohydrate biosynthetic process | 7 | -6.361197854 |
| GO Biological Processes | GO:0097327 | response to antineoplastic agent | 7 | -6.361197854 |
| GO Biological Processes | GO:1903799 | negative regulation of production of miRNAs involved in gene silencing by miRNA | 4 | -6.354502621 |
| GO Biological Processes | GO:0038127 | ERBB signaling pathway | 8 | -6.342314385 |
| GO Biological Processes | GO:0002260 | lymphocyte homeostasis | 6 | -6.3334263 |
| GO Biological Processes | GO:0071384 | cellular response to corticosteroid stimulus | 6 | -6.3334263 |
| GO Biological Processes | GO:2001244 | positive regulation of intrinsic apoptotic signaling pathway | 6 | -6.3334263 |
| GO Biological Processes | GO:1904064 | positive regulation of cation transmembrane transport | 8 | -6.319707249 |
| GO Biological Processes | GO:0060562 | epithelial tube morphogenesis | 11 | -6.317957251 |
| GO Biological Processes | GO:0010522 | regulation of calcium ion transport into cytosol | 7 | -6.301851051 |
| GO Biological Processes | GO:0015908 | fatty acid transport | 7 | -6.301851051 |
| GO Biological Processes | GO:0042102 | positive regulation of T cell proliferation | 7 | -6.301851051 |
| GO Biological Processes | GO:0010675 | regulation of cellular carbohydrate metabolic process | 8 | -6.297268929 |
| GO Biological Processes | GO:0051592 | response to calcium ion | 8 | -6.297268929 |
| GO Biological Processes | GO:0071901 | negative regulation of protein serine/threonine kinase activity | 8 | -6.297268929 |
| GO Biological Processes | GO:0043276 | anoikis | 5 | -6.285192297 |
| GO Biological Processes | GO:0043524 | negative regulation of neuron apoptotic process | 8 | -6.274997095 |
| GO Biological Processes | GO:0010952 | positive regulation of peptidase activity | 9 | -6.273048784 |
| GO Biological Processes | GO:0009792 | embryo development ending in birth or egg hatching | 15 | -6.25553292 |
| GO Biological Processes | GO:0035264 | multicellular organism growth | 8 | -6.252889464 |
| GO Biological Processes | GO:0002066 | columnar/cuboidal epithelial cell development | 6 | -6.250530545 |
| GO Biological Processes | GO:0030593 | neutrophil chemotaxis | 7 | -6.243772025 |
| GO Biological Processes | GO:1901570 | fatty acid derivative biosynthetic process | 7 | -6.243772025 |
| GO Biological Processes | GO:0002683 | negative regulation of immune system process | 13 | -6.239937429 |
| GO Biological Processes | GO:0007420 | brain development | 16 | -6.239599869 |
| GO Biological Processes | GO:0000077 | DNA damage checkpoint | 8 | -6.230943799 |
| GO Biological Processes | GO:0008643 | carbohydrate transport | 8 | -6.230943799 |
| GO Biological Processes | GO:0097530 | granulocyte migration | 8 | -6.230943799 |
| GO Biological Processes | GO:0042362 | fat-soluble vitamin biosynthetic process | 4 | -6.221381405 |
| GO Biological Processes | GO:0045073 | regulation of chemokine biosynthetic process | 4 | -6.221381405 |
| GO Biological Processes | GO:0090594 | inflammatory response to wounding | 4 | -6.221381405 |
| GO Biological Processes | GO:1904031 | positive regulation of cyclin-dependent protein kinase activity | 5 | -6.2198741 |
| GO Biological Processes | GO:0006940 | regulation of smooth muscle contraction | 6 | -6.210130027 |
| GO Biological Processes | GO:2000351 | regulation of endothelial cell apoptotic process | 6 | -6.210130027 |
| GO Biological Processes | GO:1904892 | regulation of STAT cascade | 8 | -6.209157911 |
| GO Biological Processes | GO:2000378 | negative regulation of reactive oxygen species metabolic process | 6 | -6.170398081 |
| GO Biological Processes | GO:0014065 | phosphatidylinositol 3-kinase signaling | 8 | -6.166056916 |
| GO Biological Processes | GO:2001056 | positive regulation of cysteine-type endopeptidase activity | 8 | -6.166056916 |
| GO Biological Processes | GO:0010721 | negative regulation of cell development | 11 | -6.16579385 |
| GO Biological Processes | GO:0016042 | lipid catabolic process | 11 | -6.16579385 |
| GO Biological Processes | GO:0042554 | superoxide anion generation | 5 | -6.156561755 |
| GO Biological Processes | GO:0002262 | myeloid cell homeostasis | 8 | -6.144737644 |
| GO Biological Processes | GO:0048754 | branching morphogenesis of an epithelial tube | 8 | -6.144737644 |
| GO Biological Processes | GO:0030330 | DNA damage response, signal transduction by p53 class mediator | 7 | -6.131219104 |
| GO Biological Processes | GO:0001523 | retinoid metabolic process | 7 | -6.103798163 |
| GO Biological Processes | GO:0071887 | leukocyte apoptotic process | 7 | -6.103798163 |
| GO Biological Processes | GO:0002070 | epithelial cell maturation | 4 | -6.098019832 |
| GO Biological Processes | GO:0030397 | membrane disassembly | 4 | -6.098019832 |
| GO Biological Processes | GO:0032352 | positive regulation of hormone metabolic process | 4 | -6.098019832 |
| GO Biological Processes | GO:0042033 | chemokine biosynthetic process | 4 | -6.098019832 |
| GO Biological Processes | GO:0050755 | chemokine metabolic process | 4 | -6.098019832 |
| GO Biological Processes | GO:0051081 | nuclear envelope disassembly | 4 | -6.098019832 |
| GO Biological Processes | GO:1901550 | regulation of endothelial cell development | 4 | -6.098019832 |
| GO Biological Processes | GO:1903140 | regulation of establishment of endothelial barrier | 4 | -6.098019832 |
| GO Biological Processes | GO:0042108 | positive regulation of cytokine biosynthetic process | 5 | -6.095138341 |
| GO Biological Processes | GO:0090322 | regulation of superoxide metabolic process | 5 | -6.095138341 |
| GO Biological Processes | GO:1904994 | regulation of leukocyte adhesion to vascular endothelial cell | 5 | -6.095138341 |
| GO Biological Processes | GO:0050766 | positive regulation of phagocytosis | 6 | -6.092857103 |
| GO Biological Processes | GO:0043271 | negative regulation of ion transport | 8 | -6.081680593 |
| GO Biological Processes | GO:0050729 | positive regulation of inflammatory response | 8 | -6.081680593 |
| GO Biological Processes | GO:0006939 | smooth muscle contraction | 7 | -6.076653217 |
| GO Biological Processes | GO:0043618 | regulation of transcription from RNA polymerase II promoter in response to stress | 7 | -6.076653217 |
| GO Biological Processes | GO:0090316 | positive regulation of intracellular protein transport | 8 | -6.060955353 |
| GO Biological Processes | GO:0001885 | endothelial cell development | 6 | -6.055009036 |
| GO Biological Processes | GO:1901989 | positive regulation of cell cycle phase transition | 7 | -6.049779078 |
| GO Biological Processes | GO:0046942 | carboxylic acid transport | 11 | -6.043612464 |
| GO Biological Processes | GO:0014912 | negative regulation of smooth muscle cell migration | 5 | -6.035496884 |
| GO Biological Processes | GO:0030224 | monocyte differentiation | 5 | -6.035496884 |
| GO Biological Processes | GO:0032094 | response to food | 5 | -6.035496884 |
| GO Biological Processes | GO:0043029 | T cell homeostasis | 5 | -6.035496884 |
| GO Biological Processes | GO:0045923 | positive regulation of fatty acid metabolic process | 5 | -6.035496884 |
| GO Biological Processes | GO:1903131 | mononuclear cell differentiation | 5 | -6.035496884 |
| GO Biological Processes | GO:0015849 | organic acid transport | 11 | -6.031615154 |
| GO Biological Processes | GO:0031570 | DNA integrity checkpoint | 8 | -6.01993427 |
| GO Biological Processes | GO:0034767 | positive regulation of ion transmembrane transport | 8 | -6.01993427 |
| GO Biological Processes | GO:0072577 | endothelial cell apoptotic process | 6 | -6.017751354 |
| GO Biological Processes | GO:0051301 | cell division | 14 | -6.014758267 |
| GO Biological Processes | GO:0046136 | positive regulation of vitamin metabolic process | 3 | -6.00232777 |
| GO Biological Processes | GO:0080184 | response to phenylpropanoid | 3 | -6.00232777 |
| GO Biological Processes | GO:0046822 | regulation of nucleocytoplasmic transport | 7 | -5.996823178 |
| GO Biological Processes | GO:0030730 | sequestering of triglyceride | 4 | -5.983091231 |
| GO Biological Processes | GO:0045986 | negative regulation of smooth muscle contraction | 4 | -5.983091231 |
| GO Biological Processes | GO:0050665 | hydrogen peroxide biosynthetic process | 4 | -5.983091231 |
| GO Biological Processes | GO:0006936 | muscle contraction | 11 | -5.97221259 |
| GO Biological Processes | GO:0010948 | negative regulation of cell cycle process | 11 | -5.97221259 |
| GO Biological Processes | GO:0005976 | polysaccharide metabolic process | 7 | -5.970731742 |
| GO Biological Processes | GO:0019722 | calcium-mediated signaling | 9 | -5.966825626 |
| GO Biological Processes | GO:0046661 | male sex differentiation | 8 | -5.959449164 |
| GO Biological Processes | GO:0002526 | acute inflammatory response | 9 | -5.934531943 |
| GO Biological Processes | GO:0060485 | mesenchyme development | 10 | -5.93287387 |
| GO Biological Processes | GO:0010613 | positive regulation of cardiac muscle hypertrophy | 5 | -5.921175255 |
| GO Biological Processes | GO:0045740 | positive regulation of DNA replication | 5 | -5.921175255 |
| GO Biological Processes | GO:0051281 | positive regulation of release of sequestered calcium ion into cytosol | 5 | -5.921175255 |
| GO Biological Processes | GO:0032409 | regulation of transporter activity | 10 | -5.919396049 |
| GO Biological Processes | GO:0048640 | negative regulation of developmental growth | 7 | -5.919298687 |
| GO Biological Processes | GO:0010611 | regulation of cardiac muscle hypertrophy | 6 | -5.909350365 |
| GO Biological Processes | GO:0045732 | positive regulation of protein catabolic process | 9 | -5.902561341 |
| GO Biological Processes | GO:0032386 | regulation of intracellular transport | 12 | -5.898029747 |
| GO Biological Processes | GO:0034250 | positive regulation of cellular amide metabolic process | 8 | -5.880683799 |
| GO Biological Processes | GO:0031670 | cellular response to nutrient | 6 | -5.874287587 |
| GO Biological Processes | GO:0051881 | regulation of mitochondrial membrane potential | 6 | -5.874287587 |
| GO Biological Processes | GO:0014742 | positive regulation of muscle hypertrophy | 5 | -5.866321706 |
| GO Biological Processes | GO:0071548 | response to dexamethasone | 5 | -5.866321706 |
| GO Biological Processes | GO:0038095 | Fc-epsilon receptor signaling pathway | 8 | -5.861317516 |
| GO Biological Processes | GO:0030518 | intracellular steroid hormone receptor signaling pathway | 7 | -5.843957694 |
| GO Biological Processes | GO:0016125 | sterol metabolic process | 8 | -5.842078114 |
| GO Biological Processes | GO:0048762 | mesenchymal cell differentiation | 9 | -5.839566333 |
| GO Biological Processes | GO:0042596 | fear response | 5 | -5.812901815 |
| GO Biological Processes | GO:0060969 | negative regulation of gene silencing | 5 | -5.812901815 |
| GO Biological Processes | GO:0014743 | regulation of muscle hypertrophy | 6 | -5.805679651 |
| GO Biological Processes | GO:0033077 | T cell differentiation in thymus | 6 | -5.805679651 |
| GO Biological Processes | GO:0006914 | autophagy | 13 | -5.799221423 |
| GO Biological Processes | GO:0061919 | process utilizing autophagic mechanism | 13 | -5.799221423 |
| GO Biological Processes | GO:0045861 | negative regulation of proteolysis | 11 | -5.788402118 |
| GO Biological Processes | GO:0033189 | response to vitamin A | 4 | -5.774437768 |
| GO Biological Processes | GO:0045780 | positive regulation of bone resorption | 4 | -5.774437768 |
| GO Biological Processes | GO:0046852 | positive regulation of bone remodeling | 4 | -5.774437768 |
| GO Biological Processes | GO:0051546 | keratinocyte migration | 4 | -5.774437768 |
| GO Biological Processes | GO:0060252 | positive regulation of glial cell proliferation | 4 | -5.774437768 |
| GO Biological Processes | GO:1902176 | negative regulation of oxidative stress-induced intrinsic apoptotic signaling pathway | 4 | -5.774437768 |
| GO Biological Processes | GO:0002065 | columnar/cuboidal epithelial cell differentiation | 7 | -5.770687593 |
| GO Biological Processes | GO:0044270 | cellular nitrogen compound catabolic process | 15 | -5.767548068 |
| GO Biological Processes | GO:0071364 | cellular response to epidermal growth factor stimulus | 5 | -5.7608445 |
| GO Biological Processes | GO:0150077 | regulation of neuroinflammatory response | 5 | -5.7608445 |
| GO Biological Processes | GO:1903522 | regulation of blood circulation | 10 | -5.748869411 |
| GO Biological Processes | GO:0051928 | positive regulation of calcium ion transport | 7 | -5.746706201 |
| GO Biological Processes | GO:0046777 | protein autophosphorylation | 9 | -5.732246503 |
| GO Biological Processes | GO:0016241 | regulation of macroautophagy | 8 | -5.729222923 |
| GO Biological Processes | GO:0021543 | pallium development | 8 | -5.729222923 |
| GO Biological Processes | GO:1990266 | neutrophil migration | 7 | -5.722939617 |
| GO Biological Processes | GO:0000271 | polysaccharide biosynthetic process | 6 | -5.706359949 |
| GO Biological Processes | GO:0010827 | regulation of glucose transmembrane transport | 6 | -5.706359949 |
| GO Biological Processes | GO:0060745 | mammary gland branching involved in pregnancy | 3 | -5.702789222 |
| GO Biological Processes | GO:0070141 | response to UV-A | 3 | -5.702789222 |
| GO Biological Processes | GO:0019439 | aromatic compound catabolic process | 15 | -5.680532996 |
| GO Biological Processes | GO:0007252 | I-kappaB phosphorylation | 4 | -5.679104134 |
| GO Biological Processes | GO:0051767 | nitric-oxide synthase biosynthetic process | 4 | -5.679104134 |
| GO Biological Processes | GO:0051769 | regulation of nitric-oxide synthase biosynthetic process | 4 | -5.679104134 |
| GO Biological Processes | GO:1902004 | positive regulation of amyloid-beta formation | 4 | -5.679104134 |
| GO Biological Processes | GO:1903578 | regulation of ATP metabolic process | 7 | -5.67603668 |
| GO Biological Processes | GO:0048678 | response to axon injury | 6 | -5.674161173 |
| GO Biological Processes | GO:0055021 | regulation of cardiac muscle tissue growth | 6 | -5.674161173 |
| GO Biological Processes | GO:1900182 | positive regulation of protein localization to nucleus | 6 | -5.674161173 |
| GO Biological Processes | GO:0014002 | astrocyte development | 5 | -5.6605586 |
| GO Biological Processes | GO:0060443 | mammary gland morphogenesis | 5 | -5.6605586 |
| GO Biological Processes | GO:1905330 | regulation of morphogenesis of an epithelium | 8 | -5.638402558 |
| GO Biological Processes | GO:0016570 | histone modification | 12 | -5.632862463 |
| GO Biological Processes | GO:1900180 | regulation of protein localization to nucleus | 7 | -5.629951269 |
| GO Biological Processes | GO:0032570 | response to progesterone | 5 | -5.612211773 |
| GO Biological Processes | GO:0006469 | negative regulation of protein kinase activity | 9 | -5.599421268 |
| GO Biological Processes | GO:0051346 | negative regulation of hydrolase activity | 12 | -5.595121489 |
| GO Biological Processes | GO:0009110 | vitamin biosynthetic process | 4 | -5.588909944 |
| GO Biological Processes | GO:0017000 | antibiotic biosynthetic process | 4 | -5.588909944 |
| GO Biological Processes | GO:0036499 | PERK-mediated unfolded protein response | 4 | -5.588909944 |
| GO Biological Processes | GO:0061042 | vascular wound healing | 4 | -5.588909944 |
| GO Biological Processes | GO:0071498 | cellular response to fluid shear stress | 4 | -5.588909944 |
| GO Biological Processes | GO:0010950 | positive regulation of endopeptidase activity | 8 | -5.585244174 |
| GO Biological Processes | GO:0060359 | response to ammonium ion | 7 | -5.584657193 |
| GO Biological Processes | GO:0022617 | extracellular matrix disassembly | 6 | -5.580127722 |
| GO Biological Processes | GO:0014075 | response to amine | 5 | -5.564990221 |
| GO Biological Processes | GO:0030574 | collagen catabolic process | 5 | -5.564990221 |
| GO Biological Processes | GO:0071482 | cellular response to light stimulus | 7 | -5.562299061 |
| GO Biological Processes | GO:0038061 | NIK/NF-kappaB signaling | 8 | -5.53304578 |
| GO Biological Processes | GO:0061138 | morphogenesis of a branching epithelium | 8 | -5.53304578 |
| GO Biological Processes | GO:0016569 | covalent chromatin modification | 12 | -5.511578185 |
| GO Biological Processes | GO:0001776 | leukocyte homeostasis | 6 | -5.489719859 |
| GO Biological Processes | GO:0006112 | energy reserve metabolic process | 6 | -5.489719859 |
| GO Biological Processes | GO:0035196 | production of miRNAs involved in gene silencing by miRNA | 5 | -5.473727669 |
| GO Biological Processes | GO:0070231 | T cell apoptotic process | 5 | -5.473727669 |
| GO Biological Processes | GO:0090199 | regulation of release of cytochrome c from mitochondria | 5 | -5.473727669 |
| GO Biological Processes | GO:0034765 | regulation of ion transmembrane transport | 12 | -5.465966684 |
| GO Biological Processes | GO:0031622 | positive regulation of fever generation | 3 | -5.461242321 |
| GO Biological Processes | GO:0035234 | ectopic germ cell programmed cell death | 3 | -5.461242321 |
| GO Biological Processes | GO:0060558 | regulation of calcidiol 1-monooxygenase activity | 3 | -5.461242321 |
| GO Biological Processes | GO:0061044 | negative regulation of vascular wound healing | 3 | -5.461242321 |
| GO Biological Processes | GO:0034637 | cellular carbohydrate biosynthetic process | 6 | -5.460344483 |
| GO Biological Processes | GO:0060420 | regulation of heart growth | 6 | -5.460344483 |
| GO Biological Processes | GO:0071560 | cellular response to transforming growth factor beta stimulus | 9 | -5.458165318 |
| GO Biological Processes | GO:2000027 | regulation of animal organ morphogenesis | 9 | -5.458165318 |
| GO Biological Processes | GO:0042113 | B cell activation | 10 | -5.455070873 |
| GO Biological Processes | GO:0048015 | phosphatidylinositol-mediated signaling | 8 | -5.448097801 |
| GO Biological Processes | GO:0006766 | vitamin metabolic process | 7 | -5.43200972 |
| GO Biological Processes | GO:0032642 | regulation of chemokine production | 6 | -5.431334431 |
| GO Biological Processes | GO:0046889 | positive regulation of lipid biosynthetic process | 6 | -5.431334431 |
| GO Biological Processes | GO:0010951 | negative regulation of endopeptidase activity | 9 | -5.430668797 |
| GO Biological Processes | GO:0097300 | programmed necrotic cell death | 5 | -5.429596796 |
| GO Biological Processes | GO:1904036 | negative regulation of epithelial cell apoptotic process | 5 | -5.429596796 |
| GO Biological Processes | GO:1904707 | positive regulation of vascular smooth muscle cell proliferation | 5 | -5.429596796 |
| GO Biological Processes | GO:0007219 | Notch signaling pathway | 8 | -5.398311029 |
| GO Biological Processes | GO:0071354 | cellular response to interleukin-6 | 5 | -5.386410936 |
| GO Biological Processes | GO:0048017 | inositol lipid-mediated signaling | 8 | -5.381906257 |
| GO Biological Processes | GO:0050728 | negative regulation of inflammatory response | 8 | -5.381906257 |
| GO Biological Processes | GO:0071559 | response to transforming growth factor beta | 9 | -5.376398927 |
| GO Biological Processes | GO:0006919 | activation of cysteine-type endopeptidase activity involved in apoptotic process | 6 | -5.374376554 |
| GO Biological Processes | GO:0007187 | G protein-coupled receptor signaling pathway, coupled to cyclic nucleotide second messenger | 9 | -5.362979392 |
| GO Biological Processes | GO:0006694 | steroid biosynthetic process | 8 | -5.349377021 |
| GO Biological Processes | GO:0046427 | positive regulation of JAK-STAT cascade | 6 | -5.346412581 |
| GO Biological Processes | GO:0046849 | bone remodeling | 6 | -5.346412581 |
| GO Biological Processes | GO:0060333 | interferon-gamma-mediated signaling pathway | 6 | -5.346412581 |
| GO Biological Processes | GO:1902993 | positive regulation of amyloid precursor protein catabolic process | 4 | -5.344331657 |
| GO Biological Processes | GO:0010883 | regulation of lipid storage | 5 | -5.344131802 |
| GO Biological Processes | GO:0019933 | cAMP-mediated signaling | 8 | -5.333250637 |
| GO Biological Processes | GO:1905897 | regulation of response to endoplasmic reticulum stress | 6 | -5.318781607 |
| GO Biological Processes | GO:0015711 | organic anion transport | 12 | -5.306301047 |
| GO Biological Processes | GO:0001763 | morphogenesis of a branching structure | 8 | -5.301269647 |
| GO Biological Processes | GO:0006839 | mitochondrial transport | 9 | -5.296746731 |
| GO Biological Processes | GO:0033673 | negative regulation of kinase activity | 9 | -5.296746731 |
| GO Biological Processes | GO:0072331 | signal transduction by p53 class mediator | 9 | -5.296746731 |
| GO Biological Processes | GO:0019058 | viral life cycle | 10 | -5.294977748 |
| GO Biological Processes | GO:0045185 | maintenance of protein location | 6 | -5.291476222 |
| GO Biological Processes | GO:0048598 | embryonic morphogenesis | 13 | -5.276378458 |
| GO Biological Processes | GO:0090150 | establishment of protein localization to membrane | 10 | -5.272732186 |
| GO Biological Processes | GO:0009404 | toxin metabolic process | 4 | -5.270186338 |
| GO Biological Processes | GO:0010869 | regulation of receptor biosynthetic process | 4 | -5.270186338 |
| GO Biological Processes | GO:0050927 | positive regulation of positive chemotaxis | 4 | -5.270186338 |
| GO Biological Processes | GO:1904385 | cellular response to angiotensin | 4 | -5.270186338 |
| GO Biological Processes | GO:0009746 | response to hexose | 8 | -5.269644938 |
| GO Biological Processes | GO:0034308 | primary alcohol metabolic process | 6 | -5.26448926 |
| GO Biological Processes | GO:1904894 | positive regulation of STAT cascade | 6 | -5.26448926 |
| GO Biological Processes | GO:0031050 | dsRNA processing | 5 | -5.262151794 |
| GO Biological Processes | GO:0032757 | positive regulation of interleukin-8 production | 5 | -5.262151794 |
| GO Biological Processes | GO:0070918 | production of small RNA involved in gene silencing by RNA | 5 | -5.262151794 |
| GO Biological Processes | GO:0002246 | wound healing involved in inflammatory response | 3 | -5.258613184 |
| GO Biological Processes | GO:0031620 | regulation of fever generation | 3 | -5.258613184 |
| GO Biological Processes | GO:0033084 | regulation of immature T cell proliferation in thymus | 3 | -5.258613184 |
| GO Biological Processes | GO:0008286 | insulin receptor signaling pathway | 7 | -5.248025428 |
| GO Biological Processes | GO:0002768 | immune response-regulating cell surface receptor signaling pathway | 12 | -5.246049924 |
| GO Biological Processes | GO:0010466 | negative regulation of peptidase activity | 9 | -5.244772585 |
| GO Biological Processes | GO:0032602 | chemokine production | 6 | -5.237813789 |
| GO Biological Processes | GO:1901888 | regulation of cell junction assembly | 6 | -5.237813789 |
| GO Biological Processes | GO:0051353 | positive regulation of oxidoreductase activity | 5 | -5.22238506 |
| GO Biological Processes | GO:0070741 | response to interleukin-6 | 5 | -5.22238506 |
| GO Biological Processes | GO:1904062 | regulation of cation transmembrane transport | 10 | -5.217773724 |
| GO Biological Processes | GO:0002690 | positive regulation of leukocyte chemotaxis | 6 | -5.211443101 |
| GO Biological Processes | GO:0007189 | adenylate cyclase-activating G protein-coupled receptor signaling pathway | 7 | -5.208856235 |
| GO Biological Processes | GO:0046890 | regulation of lipid biosynthetic process | 8 | -5.207436116 |
| GO Biological Processes | GO:0035902 | response to immobilization stress | 4 | -5.199210869 |
| GO Biological Processes | GO:0042730 | fibrinolysis | 4 | -5.199210869 |
| GO Biological Processes | GO:0050926 | regulation of positive chemotaxis | 4 | -5.199210869 |
| GO Biological Processes | GO:0006575 | cellular modified amino acid metabolic process | 8 | -5.192095723 |
| GO Biological Processes | GO:1900542 | regulation of purine nucleotide metabolic process | 7 | -5.189492619 |
| GO Biological Processes | GO:0007589 | body fluid secretion | 6 | -5.185370702 |
| GO Biological Processes | GO:0042100 | B cell proliferation | 6 | -5.185370702 |
| GO Biological Processes | GO:1901184 | regulation of ERBB signaling pathway | 6 | -5.185370702 |
| GO Biological Processes | GO:0048010 | vascular endothelial growth factor receptor signaling pathway | 6 | -5.159590305 |
| GO Biological Processes | GO:0050764 | regulation of phagocytosis | 6 | -5.159590305 |
| GO Biological Processes | GO:0045840 | positive regulation of mitotic nuclear division | 5 | -5.145147198 |
| GO Biological Processes | GO:0030278 | regulation of ossification | 8 | -5.131556818 |
| GO Biological Processes | GO:0001963 | synaptic transmission, dopaminergic | 4 | -5.131149941 |
| GO Biological Processes | GO:0009651 | response to salt stress | 4 | -5.131149941 |
| GO Biological Processes | GO:0033081 | regulation of T cell differentiation in thymus | 4 | -5.131149941 |
| GO Biological Processes | GO:0045672 | positive regulation of osteoclast differentiation | 4 | -5.131149941 |
| GO Biological Processes | GO:0046885 | regulation of hormone biosynthetic process | 4 | -5.131149941 |
| GO Biological Processes | GO:0048143 | astrocyte activation | 4 | -5.131149941 |
| GO Biological Processes | GO:0006140 | regulation of nucleotide metabolic process | 7 | -5.113464744 |
| GO Biological Processes | GO:1903749 | positive regulation of establishment of protein localization to mitochondrion | 5 | -5.107620623 |
| GO Biological Processes | GO:0007254 | JNK cascade | 8 | -5.101769946 |
| GO Biological Processes | GO:0016051 | carbohydrate biosynthetic process | 8 | -5.101769946 |
| GO Biological Processes | GO:0031589 | cell-substrate adhesion | 10 | -5.100048508 |
| GO Biological Processes | GO:0033044 | regulation of chromosome organization | 10 | -5.100048508 |
| GO Biological Processes | GO:0016202 | regulation of striated muscle tissue development | 7 | -5.094804838 |
| GO Biological Processes | GO:0033080 | immature T cell proliferation in thymus | 3 | -5.084012469 |
| GO Biological Processes | GO:0060556 | regulation of vitamin D biosynthetic process | 3 | -5.084012469 |
| GO Biological Processes | GO:0071550 | death-inducing signaling complex assembly | 3 | -5.084012469 |
| GO Biological Processes | GO:1901857 | positive regulation of cellular respiration | 3 | -5.084012469 |
| GO Biological Processes | GO:0008203 | cholesterol metabolic process | 7 | -5.076280057 |
| GO Biological Processes | GO:0051701 | interaction with host | 8 | -5.072296667 |
| GO Biological Processes | GO:0033598 | mammary gland epithelial cell proliferation | 4 | -5.065777953 |
| GO Biological Processes | GO:0045932 | negative regulation of muscle contraction | 4 | -5.065777953 |
| GO Biological Processes | GO:0071880 | adenylate cyclase-activating adrenergic receptor signaling pathway | 4 | -5.065777953 |
| GO Biological Processes | GO:0000079 | regulation of cyclin-dependent protein serine/threonine kinase activity | 6 | -5.059269688 |
| GO Biological Processes | GO:0043502 | regulation of muscle adaptation | 6 | -5.059269688 |
| GO Biological Processes | GO:0044264 | cellular polysaccharide metabolic process | 6 | -5.059269688 |
| GO Biological Processes | GO:0055024 | regulation of cardiac muscle tissue development | 6 | -5.059269688 |
| GO Biological Processes | GO:1903076 | regulation of protein localization to plasma membrane | 6 | -5.059269688 |
| GO Biological Processes | GO:1901861 | regulation of muscle tissue development | 7 | -5.039628764 |
| GO Biological Processes | GO:0070498 | interleukin-1-mediated signaling pathway | 6 | -5.034861586 |
| GO Biological Processes | GO:0050994 | regulation of lipid catabolic process | 5 | -5.034624507 |
| GO Biological Processes | GO:0061900 | glial cell activation | 5 | -5.034624507 |
| GO Biological Processes | GO:0000187 | activation of MAPK activity | 7 | -5.021498784 |
| GO Biological Processes | GO:0048634 | regulation of muscle organ development | 7 | -5.021498784 |
| GO Biological Processes | GO:1902652 | secondary alcohol metabolic process | 7 | -5.021498784 |
| GO Biological Processes | GO:0003300 | cardiac muscle hypertrophy | 6 | -5.010711618 |
| GO Biological Processes | GO:0060402 | calcium ion transport into cytosol | 7 | -5.003496993 |
| GO Biological Processes | GO:0060968 | regulation of gene silencing | 7 | -5.003496993 |
| GO Biological Processes | GO:0001782 | B cell homeostasis | 4 | -5.002894568 |
| GO Biological Processes | GO:0032722 | positive regulation of chemokine production | 5 | -4.999107845 |
| GO Biological Processes | GO:0035051 | cardiocyte differentiation | 7 | -4.985621742 |
| GO Biological Processes | GO:0010803 | regulation of tumor necrosis factor-mediated signaling pathway | 5 | -4.964216022 |
| GO Biological Processes | GO:1904029 | regulation of cyclin-dependent protein kinase activity | 6 | -4.963165962 |
| GO Biological Processes | GO:0019935 | cyclic-nucleotide-mediated signaling | 8 | -4.957424277 |
| GO Biological Processes | GO:0051047 | positive regulation of secretion | 10 | -4.956198593 |
| GO Biological Processes | GO:0045088 | regulation of innate immune response | 11 | -4.954721783 |
| GO Biological Processes | GO:0002275 | myeloid cell activation involved in immune response | 12 | -4.943379082 |
| GO Biological Processes | GO:0001516 | prostaglandin biosynthetic process | 4 | -4.942321067 |
| GO Biological Processes | GO:0005979 | regulation of glycogen biosynthetic process | 4 | -4.942321067 |
| GO Biological Processes | GO:0010962 | regulation of glucan biosynthetic process | 4 | -4.942321067 |
| GO Biological Processes | GO:0043457 | regulation of cellular respiration | 4 | -4.942321067 |
| GO Biological Processes | GO:0046457 | prostanoid biosynthetic process | 4 | -4.942321067 |
| GO Biological Processes | GO:0060055 | angiogenesis involved in wound healing | 4 | -4.942321067 |
| GO Biological Processes | GO:0014897 | striated muscle hypertrophy | 6 | -4.939760575 |
| GO Biological Processes | GO:0032611 | interleukin-1 beta production | 6 | -4.939760575 |
| GO Biological Processes | GO:0055017 | cardiac muscle tissue growth | 6 | -4.939760575 |
| GO Biological Processes | GO:0010887 | negative regulation of cholesterol storage | 3 | -4.930600751 |
| GO Biological Processes | GO:0032025 | response to cobalt ion | 3 | -4.930600751 |
| GO Biological Processes | GO:0033083 | regulation of immature T cell proliferation | 3 | -4.930600751 |
| GO Biological Processes | GO:0071394 | cellular response to testosterone stimulus | 3 | -4.930600751 |
| GO Biological Processes | GO:0097267 | omega-hydroxylase P450 pathway | 3 | -4.930600751 |
| GO Biological Processes | GO:0032507 | maintenance of protein location in cell | 5 | -4.929928343 |
| GO Biological Processes | GO:0046622 | positive regulation of organ growth | 5 | -4.929928343 |
| GO Biological Processes | GO:0032388 | positive regulation of intracellular transport | 8 | -4.915544315 |
| GO Biological Processes | GO:0031214 | biomineral tissue development | 7 | -4.915354209 |
| GO Biological Processes | GO:0000904 | cell morphogenesis involved in differentiation | 14 | -4.907703728 |
| GO Biological Processes | GO:0006959 | humoral immune response | 10 | -4.90635949 |
| GO Biological Processes | GO:0046165 | alcohol biosynthetic process | 7 | -4.898087976 |
| GO Biological Processes | GO:0045453 | bone resorption | 5 | -4.896225122 |
| GO Biological Processes | GO:0014896 | muscle hypertrophy | 6 | -4.893661471 |
| GO Biological Processes | GO:0007162 | negative regulation of cell adhesion | 9 | -4.892497659 |
| GO Biological Processes | GO:0002675 | positive regulation of acute inflammatory response | 4 | -4.883897344 |
| GO Biological Processes | GO:0045737 | positive regulation of cyclin-dependent protein serine/threonine kinase activity | 4 | -4.883897344 |
| GO Biological Processes | GO:0006820 | anion transport | 13 | -4.879229693 |
| GO Biological Processes | GO:0070265 | necrotic cell death | 5 | -4.863087622 |
| GO Biological Processes | GO:0034440 | lipid oxidation | 6 | -4.848481824 |
| GO Biological Processes | GO:0007188 | adenylate cyclase-modulating G protein-coupled receptor signaling pathway | 8 | -4.833645426 |
| GO Biological Processes | GO:0008210 | estrogen metabolic process | 4 | -4.827479404 |
| GO Biological Processes | GO:0038128 | ERBB2 signaling pathway | 4 | -4.827479404 |
| GO Biological Processes | GO:0046320 | regulation of fatty acid oxidation | 4 | -4.827479404 |
| GO Biological Processes | GO:0051385 | response to mineralocorticoid | 4 | -4.827479404 |
| GO Biological Processes | GO:0051968 | positive regulation of synaptic transmission, glutamatergic | 4 | -4.827479404 |
| GO Biological Processes | GO:0071875 | adrenergic receptor signaling pathway | 4 | -4.827479404 |
| GO Biological Processes | GO:0006275 | regulation of DNA replication | 6 | -4.826226215 |
| GO Biological Processes | GO:0055002 | striated muscle cell development | 7 | -4.813487409 |
| GO Biological Processes | GO:2000278 | regulation of DNA biosynthetic process | 6 | -4.804188004 |
| GO Biological Processes | GO:0006879 | cellular iron ion homeostasis | 5 | -4.798439214 |
| GO Biological Processes | GO:0042035 | regulation of cytokine biosynthetic process | 5 | -4.798439214 |
| GO Biological Processes | GO:0045670 | regulation of osteoclast differentiation | 5 | -4.798439214 |
| GO Biological Processes | GO:0050918 | positive chemotaxis | 5 | -4.798439214 |
| GO Biological Processes | GO:0031652 | positive regulation of heat generation | 3 | -4.793787993 |
| GO Biological Processes | GO:0033079 | immature T cell proliferation | 3 | -4.793787993 |
| GO Biological Processes | GO:0033483 | gas homeostasis | 3 | -4.793787993 |
| GO Biological Processes | GO:0043619 | regulation of transcription from RNA polymerase II promoter in response to oxidative stress | 3 | -4.793787993 |
| GO Biological Processes | GO:0051302 | regulation of cell division | 7 | -4.780428909 |
| GO Biological Processes | GO:0001975 | response to amphetamine | 4 | -4.772937271 |
| GO Biological Processes | GO:1902003 | regulation of amyloid-beta formation | 4 | -4.772937271 |
| GO Biological Processes | GO:0070372 | regulation of ERK1 and ERK2 cascade | 9 | -4.769053067 |
| GO Biological Processes | GO:0040014 | regulation of multicellular organism growth | 5 | -4.766895051 |
| GO Biological Processes | GO:0045123 | cellular extravasation | 5 | -4.766895051 |
| GO Biological Processes | GO:0071479 | cellular response to ionizing radiation | 5 | -4.766895051 |
| GO Biological Processes | GO:0060419 | heart growth | 6 | -4.760748193 |
| GO Biological Processes | GO:0033157 | regulation of intracellular protein transport | 8 | -4.754121176 |
| GO Biological Processes | GO:0006937 | regulation of muscle contraction | 7 | -4.747801209 |
| GO Biological Processes | GO:0060401 | cytosolic calcium ion transport | 7 | -4.747801209 |
| GO Biological Processes | GO:0060964 | regulation of gene silencing by miRNA | 6 | -4.739339061 |
| GO Biological Processes | GO:0032370 | positive regulation of lipid transport | 5 | -4.735849997 |
| GO Biological Processes | GO:0038093 | Fc receptor signaling pathway | 8 | -4.728119852 |
| GO Biological Processes | GO:0016242 | negative regulation of macroautophagy | 4 | -4.720153218 |
| GO Biological Processes | GO:0042311 | vasodilation | 4 | -4.720153218 |
| GO Biological Processes | GO:0046685 | response to arsenic-containing substance | 4 | -4.720153218 |
| GO Biological Processes | GO:0051187 | cofactor catabolic process | 5 | -4.705289241 |
| GO Biological Processes | GO:0072347 | response to anesthetic | 5 | -4.705289241 |
| GO Biological Processes | GO:0014823 | response to activity | 5 | -4.675198619 |
| GO Biological Processes | GO:0032371 | regulation of sterol transport | 5 | -4.675198619 |
| GO Biological Processes | GO:0032374 | regulation of cholesterol transport | 5 | -4.675198619 |
| GO Biological Processes | GO:0033627 | cell adhesion mediated by integrin | 5 | -4.675198619 |
| GO Biological Processes | GO:0051785 | positive regulation of nuclear division | 5 | -4.675198619 |
| GO Biological Processes | GO:0006983 | ER overload response | 3 | -4.670338895 |
| GO Biological Processes | GO:0051549 | positive regulation of keratinocyte migration | 3 | -4.670338895 |
| GO Biological Processes | GO:0006691 | leukotriene metabolic process | 4 | -4.669020277 |
| GO Biological Processes | GO:0060251 | regulation of glial cell proliferation | 4 | -4.669020277 |
| GO Biological Processes | GO:0071312 | cellular response to alkaloid | 4 | -4.669020277 |
| GO Biological Processes | GO:0007204 | positive regulation of cytosolic calcium ion concentration | 9 | -4.661166762 |
| GO Biological Processes | GO:0060147 | regulation of posttranscriptional gene silencing | 6 | -4.655691022 |
| GO Biological Processes | GO:0060966 | regulation of gene silencing by RNA | 6 | -4.655691022 |
| GO Biological Processes | GO:0042593 | glucose homeostasis | 8 | -4.651577248 |
| GO Biological Processes | GO:0033692 | cellular polysaccharide biosynthetic process | 5 | -4.645564581 |
| GO Biological Processes | GO:0042089 | cytokine biosynthetic process | 5 | -4.645564581 |
| GO Biological Processes | GO:1904427 | positive regulation of calcium ion transmembrane transport | 5 | -4.645564581 |
| GO Biological Processes | GO:0033500 | carbohydrate homeostasis | 8 | -4.639027997 |
| GO Biological Processes | GO:0021987 | cerebral cortex development | 6 | -4.635259191 |
| GO Biological Processes | GO:0051193 | regulation of cofactor metabolic process | 6 | -4.635259191 |
| GO Biological Processes | GO:0045742 | positive regulation of epidermal growth factor receptor signaling pathway | 4 | -4.619440965 |
| GO Biological Processes | GO:0070873 | regulation of glycogen metabolic process | 4 | -4.619440965 |
| GO Biological Processes | GO:1905332 | positive regulation of morphogenesis of an epithelium | 4 | -4.619440965 |
| GO Biological Processes | GO:0005977 | glycogen metabolic process | 5 | -4.616374151 |
| GO Biological Processes | GO:0042107 | cytokine metabolic process | 5 | -4.616374151 |
| GO Biological Processes | GO:0051966 | regulation of synaptic transmission, glutamatergic | 5 | -4.616374151 |
| GO Biological Processes | GO:0070227 | lymphocyte apoptotic process | 5 | -4.616374151 |
| GO Biological Processes | GO:1903747 | regulation of establishment of protein localization to mitochondrion | 5 | -4.616374151 |
| GO Biological Processes | GO:0032612 | interleukin-1 production | 6 | -4.615012952 |
| GO Biological Processes | GO:0045446 | endothelial cell differentiation | 6 | -4.615012952 |
| GO Biological Processes | GO:1901222 | regulation of NIK/NF-kappaB signaling | 6 | -4.615012952 |
| GO Biological Processes | GO:0071346 | cellular response to interferon-gamma | 7 | -4.606045228 |
| GO Biological Processes | GO:1904375 | regulation of protein localization to cell periphery | 6 | -4.594949211 |
| GO Biological Processes | GO:0006986 | response to unfolded protein | 7 | -4.590783521 |
| GO Biological Processes | GO:0043433 | negative regulation of DNA-binding transcription factor activity | 7 | -4.590783521 |
| GO Biological Processes | GO:0055001 | muscle cell development | 7 | -4.590783521 |
| GO Biological Processes | GO:0042119 | neutrophil activation | 11 | -4.58808672 |
| GO Biological Processes | GO:0006073 | cellular glucan metabolic process | 5 | -4.587614904 |
| GO Biological Processes | GO:0044042 | glucan metabolic process | 5 | -4.587614904 |
| GO Biological Processes | GO:0030212 | hyaluronan metabolic process | 4 | -4.571326191 |
| GO Biological Processes | GO:0046328 | regulation of JNK cascade | 7 | -4.560540468 |
| GO Biological Processes | GO:0045685 | regulation of glial cell differentiation | 5 | -4.559274925 |
| GO Biological Processes | GO:0046323 | glucose import | 5 | -4.559274925 |
| GO Biological Processes | GO:0010745 | negative regulation of macrophage derived foam cell differentiation | 3 | -4.557884878 |
| GO Biological Processes | GO:0016264 | gap junction assembly | 3 | -4.557884878 |
| GO Biological Processes | GO:0030656 | regulation of vitamin metabolic process | 3 | -4.557884878 |
| GO Biological Processes | GO:0031650 | regulation of heat generation | 3 | -4.557884878 |
| GO Biological Processes | GO:0061043 | regulation of vascular wound healing | 3 | -4.557884878 |
| GO Biological Processes | GO:0030968 | endoplasmic reticulum unfolded protein response | 6 | -4.555357211 |
| GO Biological Processes | GO:0036230 | granulocyte activation | 11 | -4.541137326 |
| GO Biological Processes | GO:0016485 | protein processing | 9 | -4.526657293 |
| GO Biological Processes | GO:0010742 | macrophage derived foam cell differentiation | 4 | -4.524594321 |
| GO Biological Processes | GO:0090077 | foam cell differentiation | 4 | -4.524594321 |
| GO Biological Processes | GO:1901186 | positive regulation of ERBB signaling pathway | 4 | -4.524594321 |
| GO Biological Processes | GO:0072507 | divalent inorganic cation homeostasis | 11 | -4.517917454 |
| GO Biological Processes | GO:0034329 | cell junction assembly | 8 | -4.51665422 |
| GO Biological Processes | GO:0014066 | regulation of phosphatidylinositol 3-kinase signaling | 6 | -4.516459909 |
| GO Biological Processes | GO:0007422 | peripheral nervous system development | 5 | -4.503807531 |
| GO Biological Processes | GO:0010508 | positive regulation of autophagy | 6 | -4.497264799 |
| GO Biological Processes | GO:0051607 | defense response to virus | 8 | -4.49283669 |
| GO Biological Processes | GO:0001501 | skeletal system development | 11 | -4.479588267 |
| GO Biological Processes | GO:0014037 | Schwann cell differentiation | 4 | -4.479170367 |
| GO Biological Processes | GO:0034205 | amyloid-beta formation | 4 | -4.479170367 |
| GO Biological Processes | GO:1904706 | negative regulation of vascular smooth muscle cell proliferation | 4 | -4.479170367 |
| GO Biological Processes | GO:0072332 | intrinsic apoptotic signaling pathway by p53 class mediator | 5 | -4.476658621 |
| GO Biological Processes | GO:0034248 | regulation of cellular amide metabolic process | 11 | -4.471977367 |
| GO Biological Processes | GO:0098542 | defense response to other organism | 12 | -4.46121432 |
| GO Biological Processes | GO:0034101 | erythrocyte homeostasis | 6 | -4.459368305 |
| GO Biological Processes | GO:0021537 | telencephalon development | 8 | -4.457505191 |
| GO Biological Processes | GO:1901617 | organic hydroxy compound biosynthetic process | 8 | -4.457505191 |
| GO Biological Processes | GO:1903532 | positive regulation of secretion by cell | 9 | -4.456756404 |
| GO Biological Processes | GO:0030213 | hyaluronan biosynthetic process | 3 | -4.454638559 |
| GO Biological Processes | GO:0051547 | regulation of keratinocyte migration | 3 | -4.454638559 |
| GO Biological Processes | GO:0090399 | replicative senescence | 3 | -4.454638559 |
| GO Biological Processes | GO:1902894 | negative regulation of pri-miRNA transcription by RNA polymerase II | 3 | -4.454638559 |
| GO Biological Processes | GO:0001662 | behavioral fear response | 4 | -4.434985285 |
| GO Biological Processes | GO:0007190 | activation of adenylate cyclase activity | 4 | -4.434985285 |
| GO Biological Processes | GO:0032350 | regulation of hormone metabolic process | 4 | -4.434985285 |
| GO Biological Processes | GO:0042307 | positive regulation of protein import into nucleus | 4 | -4.434985285 |
| GO Biological Processes | GO:0045746 | negative regulation of Notch signaling pathway | 4 | -4.434985285 |
| GO Biological Processes | GO:1902991 | regulation of amyloid precursor protein catabolic process | 4 | -4.434985285 |
| GO Biological Processes | GO:2000279 | negative regulation of DNA biosynthetic process | 4 | -4.434985285 |
| GO Biological Processes | GO:1901224 | positive regulation of NIK/NF-kappaB signaling | 5 | -4.423479779 |
| GO Biological Processes | GO:0042476 | odontogenesis | 6 | -4.422113023 |
| GO Biological Processes | GO:0051279 | regulation of release of sequestered calcium ion into cytosol | 5 | -4.397430772 |
| GO Biological Processes | GO:0002209 | behavioral defense response | 4 | -4.39197536 |
| GO Biological Processes | GO:0031348 | negative regulation of defense response | 8 | -4.388223831 |
| GO Biological Processes | GO:0009749 | response to glucose | 7 | -4.372563681 |
| GO Biological Processes | GO:0030258 | lipid modification | 8 | -4.365528105 |
| GO Biological Processes | GO:0045651 | positive regulation of macrophage differentiation | 3 | -4.359217275 |
| GO Biological Processes | GO:0051044 | positive regulation of membrane protein ectodomain proteolysis | 3 | -4.359217275 |
| GO Biological Processes | GO:0072595 | maintenance of protein localization in organelle | 4 | -4.350081673 |
| GO Biological Processes | GO:1904591 | positive regulation of protein import | 4 | -4.350081673 |
| GO Biological Processes | GO:0014855 | striated muscle cell proliferation | 5 | -4.34636858 |
| GO Biological Processes | GO:0055013 | cardiac muscle cell development | 5 | -4.34636858 |
| GO Biological Processes | GO:1902930 | regulation of alcohol biosynthetic process | 5 | -4.34636858 |
| GO Biological Processes | GO:0002253 | activation of immune response | 13 | -4.337026125 |
| GO Biological Processes | GO:0051651 | maintenance of location in cell | 5 | -4.32133839 |
| GO Biological Processes | GO:0055072 | iron ion homeostasis | 5 | -4.32133839 |
| GO Biological Processes | GO:0034341 | response to interferon-gamma | 7 | -4.317530199 |
| GO Biological Processes | GO:0043299 | leukocyte degranulation | 11 | -4.316234265 |
| GO Biological Processes | GO:0022898 | regulation of transmembrane transporter activity | 8 | -4.309631603 |
| GO Biological Processes | GO:0045124 | regulation of bone resorption | 4 | -4.30924963 |
| GO Biological Processes | GO:0051480 | regulation of cytosolic calcium ion concentration | 9 | -4.303166884 |
| GO Biological Processes | GO:0045844 | positive regulation of striated muscle tissue development | 5 | -4.296631318 |
| GO Biological Processes | GO:0048636 | positive regulation of muscle organ development | 5 | -4.296631318 |
| GO Biological Processes | GO:0002221 | pattern recognition receptor signaling pathway | 7 | -4.277063504 |
| GO Biological Processes | GO:0035966 | response to topologically incorrect protein | 7 | -4.277063504 |
| GO Biological Processes | GO:0009791 | post-embryonic development | 5 | -4.272239613 |
| GO Biological Processes | GO:0045445 | myoblast differentiation | 5 | -4.272239613 |
| GO Biological Processes | GO:1901863 | positive regulation of muscle tissue development | 5 | -4.272239613 |
| GO Biological Processes | GO:0019372 | lipoxygenase pathway | 3 | -4.27052907 |
| GO Biological Processes | GO:0032225 | regulation of synaptic transmission, dopaminergic | 3 | -4.27052907 |
| GO Biological Processes | GO:0051770 | positive regulation of nitric-oxide synthase biosynthetic process | 3 | -4.27052907 |
| GO Biological Processes | GO:0060576 | intestinal epithelial cell development | 3 | -4.27052907 |
| GO Biological Processes | GO:0071732 | cellular response to nitric oxide | 3 | -4.27052907 |
| GO Biological Processes | GO:1900451 | positive regulation of glutamate receptor signaling pathway | 3 | -4.27052907 |
| GO Biological Processes | GO:0033173 | calcineurin-NFAT signaling cascade | 4 | -4.269428548 |
| GO Biological Processes | GO:0045687 | positive regulation of glial cell differentiation | 4 | -4.269428548 |
| GO Biological Processes | GO:0007179 | transforming growth factor beta receptor signaling pathway | 7 | -4.263724517 |
| GO Biological Processes | GO:0001889 | liver development | 6 | -4.261891942 |
| GO Biological Processes | GO:0007043 | cell-cell junction assembly | 6 | -4.261891942 |
| GO Biological Processes | GO:0009308 | amine metabolic process | 6 | -4.261891942 |
| GO Biological Processes | GO:0050714 | positive regulation of protein secretion | 7 | -4.250459255 |
| GO Biological Processes | GO:0010507 | negative regulation of autophagy | 5 | -4.248155802 |
| GO Biological Processes | GO:0042509 | regulation of tyrosine phosphorylation of STAT protein | 5 | -4.248155802 |
| GO Biological Processes | GO:0051781 | positive regulation of cell division | 5 | -4.248155802 |
| GO Biological Processes | GO:0034198 | cellular response to amino acid starvation | 4 | -4.230571292 |
| GO Biological Processes | GO:0070266 | necroptotic process | 4 | -4.230571292 |
| GO Biological Processes | GO:0090311 | regulation of protein deacetylation | 4 | -4.230571292 |
| GO Biological Processes | GO:0070098 | chemokine-mediated signaling pathway | 5 | -4.224372672 |
| GO Biological Processes | GO:0016579 | protein deubiquitination | 8 | -4.222611169 |
| GO Biological Processes | GO:0006874 | cellular calcium ion homeostasis | 10 | -4.217790004 |
| GO Biological Processes | GO:0042737 | drug catabolic process | 6 | -4.211004361 |
| GO Biological Processes | GO:0061008 | hepaticobiliary system development | 6 | -4.211004361 |
| GO Biological Processes | GO:0106106 | cold-induced thermogenesis | 6 | -4.211004361 |
| GO Biological Processes | GO:0120161 | regulation of cold-induced thermogenesis | 6 | -4.211004361 |
| GO Biological Processes | GO:0055006 | cardiac cell development | 5 | -4.200883262 |
| GO Biological Processes | GO:0002757 | immune response-activating signal transduction | 12 | -4.199518046 |
| GO Biological Processes | GO:0007612 | learning | 6 | -4.19430454 |
| GO Biological Processes | GO:0005978 | glycogen biosynthetic process | 4 | -4.192633951 |
| GO Biological Processes | GO:0009250 | glucan biosynthetic process | 4 | -4.192633951 |
| GO Biological Processes | GO:0010874 | regulation of cholesterol efflux | 4 | -4.192633951 |
| GO Biological Processes | GO:0048538 | thymus development | 4 | -4.192633951 |
| GO Biological Processes | GO:0002444 | myeloid leukocyte mediated immunity | 11 | -4.188643638 |
| GO Biological Processes | GO:0010224 | response to UV-B | 3 | -4.187696306 |
| GO Biological Processes | GO:0030949 | positive regulation of vascular endothelial growth factor receptor signaling pathway | 3 | -4.187696306 |
| GO Biological Processes | GO:0070242 | thymocyte apoptotic process | 3 | -4.187696306 |
| GO Biological Processes | GO:2001267 | regulation of cysteine-type endopeptidase activity involved in apoptotic signaling pathway | 3 | -4.187696306 |
| GO Biological Processes | GO:0034620 | cellular response to unfolded protein | 6 | -4.177733002 |
| GO Biological Processes | GO:0007260 | tyrosine phosphorylation of STAT protein | 5 | -4.177680849 |
| GO Biological Processes | GO:0042058 | regulation of epidermal growth factor receptor signaling pathway | 5 | -4.177680849 |
| GO Biological Processes | GO:0006953 | acute-phase response | 4 | -4.155575546 |
| GO Biological Processes | GO:0022602 | ovulation cycle process | 4 | -4.155575546 |
| GO Biological Processes | GO:0042475 | odontogenesis of dentin-containing tooth | 5 | -4.15475894 |
| GO Biological Processes | GO:2000177 | regulation of neural precursor cell proliferation | 5 | -4.15475894 |
| GO Biological Processes | GO:0030260 | entry into host cell | 6 | -4.14496768 |
| GO Biological Processes | GO:0044409 | entry into host | 6 | -4.14496768 |
| GO Biological Processes | GO:0051806 | entry into cell of other organism involved in symbiotic interaction | 6 | -4.14496768 |
| GO Biological Processes | GO:0051828 | entry into other organism involved in symbiotic interaction | 6 | -4.128770436 |
| GO Biological Processes | GO:0055074 | calcium ion homeostasis | 10 | -4.124586595 |
| GO Biological Processes | GO:0010810 | regulation of cell-substrate adhesion | 7 | -4.121707746 |
| GO Biological Processes | GO:0033628 | regulation of cell adhesion mediated by integrin | 4 | -4.11935778 |
| GO Biological Processes | GO:0055023 | positive regulation of cardiac muscle tissue growth | 4 | -4.11935778 |
| GO Biological Processes | GO:0060324 | face development | 4 | -4.11935778 |
| GO Biological Processes | GO:1903580 | positive regulation of ATP metabolic process | 4 | -4.11935778 |
| GO Biological Processes | GO:1990928 | response to amino acid starvation | 4 | -4.11935778 |
| GO Biological Processes | GO:0045580 | regulation of T cell differentiation | 6 | -4.112694558 |
| GO Biological Processes | GO:0010885 | regulation of cholesterol storage | 3 | -4.110002877 |
| GO Biological Processes | GO:0032677 | regulation of interleukin-8 production | 5 | -4.109731732 |
| GO Biological Processes | GO:1905954 | positive regulation of lipid localization | 5 | -4.109731732 |
| GO Biological Processes | GO:0050768 | negative regulation of neurogenesis | 8 | -4.107538323 |
| GO Biological Processes | GO:0034655 | nucleobase-containing compound catabolic process | 12 | -4.094394035 |
| GO Biological Processes | GO:0045664 | regulation of neuron differentiation | 12 | -4.088316909 |
| GO Biological Processes | GO:0002686 | negative regulation of leukocyte migration | 4 | -4.083944805 |
| GO Biological Processes | GO:0042149 | cellular response to glucose starvation | 4 | -4.083944805 |
| GO Biological Processes | GO:0045601 | regulation of endothelial cell differentiation | 4 | -4.083944805 |
| GO Biological Processes | GO:0048512 | circadian behavior | 4 | -4.083944805 |
| GO Biological Processes | GO:0060711 | labyrinthine layer development | 4 | -4.083944805 |
| GO Biological Processes | GO:0097720 | calcineurin-mediated signaling | 4 | -4.083944805 |
| GO Biological Processes | GO:1900087 | positive regulation of G1/S transition of mitotic cell cycle | 4 | -4.083944805 |
| GO Biological Processes | GO:0001837 | epithelial to mesenchymal transition | 6 | -4.065178859 |
| GO Biological Processes | GO:0031056 | regulation of histone modification | 6 | -4.065178859 |
| GO Biological Processes | GO:0071774 | response to fibroblast growth factor | 6 | -4.065178859 |
| GO Biological Processes | GO:0007622 | rhythmic behavior | 4 | -4.049303022 |
| GO Biological Processes | GO:0014009 | glial cell proliferation | 4 | -4.049303022 |
| GO Biological Processes | GO:0030195 | negative regulation of blood coagulation | 4 | -4.049303022 |
| GO Biological Processes | GO:0042987 | amyloid precursor protein catabolic process | 4 | -4.049303022 |
| GO Biological Processes | GO:0046850 | regulation of bone remodeling | 4 | -4.049303022 |
| GO Biological Processes | GO:0050873 | brown fat cell differentiation | 4 | -4.049303022 |
| GO Biological Processes | GO:0070646 | protein modification by small protein removal | 8 | -4.046879492 |
| GO Biological Processes | GO:0010660 | regulation of muscle cell apoptotic process | 5 | -4.044144363 |
| GO Biological Processes | GO:0035249 | synaptic transmission, glutamatergic | 5 | -4.044144363 |
| GO Biological Processes | GO:0032930 | positive regulation of superoxide anion generation | 3 | -4.036856771 |
| GO Biological Processes | GO:1902170 | cellular response to reactive nitrogen species | 3 | -4.036856771 |
| GO Biological Processes | GO:0002224 | toll-like receptor signaling pathway | 6 | -4.03407931 |
| GO Biological Processes | GO:1990845 | adaptive thermogenesis | 6 | -4.03407931 |
| GO Biological Processes | GO:0120162 | positive regulation of cold-induced thermogenesis | 5 | -4.022780647 |
| GO Biological Processes | GO:1990868 | response to chemokine | 5 | -4.022780647 |
| GO Biological Processes | GO:1990869 | cellular response to chemokine | 5 | -4.022780647 |
| GO Biological Processes | GO:0030902 | hindbrain development | 6 | -4.018698265 |
| GO Biological Processes | GO:0050435 | amyloid-beta metabolic process | 4 | -4.015400889 |
| GO Biological Processes | GO:0051972 | regulation of telomerase activity | 4 | -4.015400889 |
| GO Biological Processes | GO:0071675 | regulation of mononuclear cell migration | 4 | -4.015400889 |
| GO Biological Processes | GO:1900047 | negative regulation of hemostasis | 4 | -4.015400889 |
| GO Biological Processes | GO:2001020 | regulation of response to DNA damage stimulus | 7 | -4.011525997 |
| GO Biological Processes | GO:0030900 | forebrain development | 9 | -4.002464862 |
| GO Biological Processes | GO:0030316 | osteoclast differentiation | 5 | -4.001657569 |
| GO Biological Processes | GO:0032651 | regulation of interleukin-1 beta production | 5 | -4.001657569 |
| GO Biological Processes | GO:0071674 | mononuclear cell migration | 5 | -4.001657569 |
| GO Biological Processes | GO:0002758 | innate immune response-activating signal transduction | 8 | -3.997411427 |
| GO Biological Processes | GO:0002793 | positive regulation of peptide secretion | 7 | -3.987727554 |
| GO Biological Processes | GO:0043312 | neutrophil degranulation | 10 | -3.982880967 |
| GO Biological Processes | GO:0000186 | activation of MAPKK activity | 4 | -3.982208766 |
| GO Biological Processes | GO:0006998 | nuclear envelope organization | 4 | -3.982208766 |
| GO Biological Processes | GO:0060421 | positive regulation of heart growth | 4 | -3.982208766 |
| GO Biological Processes | GO:0050810 | regulation of steroid biosynthetic process | 5 | -3.980770137 |
| GO Biological Processes | GO:0010875 | positive regulation of cholesterol efflux | 3 | -3.967762904 |
| GO Biological Processes | GO:0010878 | cholesterol storage | 3 | -3.967762904 |
| GO Biological Processes | GO:0019373 | epoxygenase P450 pathway | 3 | -3.967762904 |
| GO Biological Processes | GO:0031998 | regulation of fatty acid beta-oxidation | 3 | -3.967762904 |
| GO Biological Processes | GO:0051412 | response to corticosterone | 3 | -3.967762904 |
| GO Biological Processes | GO:0060353 | regulation of cell adhesion molecule production | 3 | -3.967762904 |
| GO Biological Processes | GO:0071731 | response to nitric oxide | 3 | -3.967762904 |
| GO Biological Processes | GO:1900409 | positive regulation of cellular response to oxidative stress | 3 | -3.967762904 |
| GO Biological Processes | GO:0002283 | neutrophil activation involved in immune response | 10 | -3.961132751 |
| GO Biological Processes | GO:0010657 | muscle cell apoptotic process | 5 | -3.96011351 |
| GO Biological Processes | GO:0070301 | cellular response to hydrogen peroxide | 5 | -3.96011351 |
| GO Biological Processes | GO:0007160 | cell-matrix adhesion | 7 | -3.952478538 |
| GO Biological Processes | GO:0015893 | drug transport | 7 | -3.952478538 |
| GO Biological Processes | GO:0032731 | positive regulation of interleukin-1 beta production | 4 | -3.949698755 |
| GO Biological Processes | GO:0043124 | negative regulation of I-kappaB kinase/NF-kappaB signaling | 4 | -3.949698755 |
| GO Biological Processes | GO:0043392 | negative regulation of DNA binding | 4 | -3.949698755 |
| GO Biological Processes | GO:0045661 | regulation of myoblast differentiation | 4 | -3.949698755 |
| GO Biological Processes | GO:0048260 | positive regulation of receptor-mediated endocytosis | 4 | -3.949698755 |
| GO Biological Processes | GO:0046034 | ATP metabolic process | 8 | -3.948891615 |
| GO Biological Processes | GO:0072503 | cellular divalent inorganic cation homeostasis | 10 | -3.946725712 |
| GO Biological Processes | GO:0048738 | cardiac muscle tissue development | 7 | -3.940846404 |
| GO Biological Processes | GO:0032637 | interleukin-8 production | 5 | -3.939682996 |
| GO Biological Processes | GO:0043903 | regulation of symbiosis, encompassing mutualism through parasitism | 7 | -3.929272184 |
| GO Biological Processes | GO:0002673 | regulation of acute inflammatory response | 6 | -3.914047825 |
| GO Biological Processes | GO:0032990 | cell part morphogenesis | 12 | -3.911257115 |
| GO Biological Processes | GO:0016236 | macroautophagy | 8 | -3.910737862 |
| GO Biological Processes | GO:0010829 | negative regulation of glucose transmembrane transport | 3 | -3.902303017 |
| GO Biological Processes | GO:0010893 | positive regulation of steroid biosynthetic process | 3 | -3.902303017 |
| GO Biological Processes | GO:0019370 | leukotriene biosynthetic process | 3 | -3.902303017 |
| GO Biological Processes | GO:0043651 | linoleic acid metabolic process | 3 | -3.902303017 |
| GO Biological Processes | GO:0046827 | positive regulation of protein export from nucleus | 3 | -3.902303017 |
| GO Biological Processes | GO:0090312 | positive regulation of protein deacetylation | 3 | -3.902303017 |
| GO Biological Processes | GO:0097709 | connective tissue replacement | 3 | -3.902303017 |
| GO Biological Processes | GO:0051604 | protein maturation | 9 | -3.887686904 |
| GO Biological Processes | GO:0030199 | collagen fibril organization | 4 | -3.886621432 |
| GO Biological Processes | GO:0050819 | negative regulation of coagulation | 4 | -3.886621432 |
| GO Biological Processes | GO:0007088 | regulation of mitotic nuclear division | 6 | -3.885077131 |
| GO Biological Processes | GO:0002446 | neutrophil mediated immunity | 10 | -3.882785394 |
| GO Biological Processes | GO:0034330 | cell junction organization | 8 | -3.88249887 |
| GO Biological Processes | GO:0051961 | negative regulation of nervous system development | 8 | -3.88249887 |
| GO Biological Processes | GO:0034766 | negative regulation of ion transmembrane transport | 5 | -3.879703295 |
| GO Biological Processes | GO:2000060 | positive regulation of ubiquitin-dependent protein catabolic process | 5 | -3.879703295 |
| GO Biological Processes | GO:0035967 | cellular response to topologically incorrect protein | 6 | -3.870740319 |
| GO Biological Processes | GO:0031295 | T cell costimulation | 4 | -3.856005915 |
| GO Biological Processes | GO:0060688 | regulation of morphogenesis of a branching structure | 4 | -3.856005915 |
| GO Biological Processes | GO:0090183 | regulation of kidney development | 4 | -3.856005915 |
| GO Biological Processes | GO:0007276 | gamete generation | 12 | -3.843164251 |
| GO Biological Processes | GO:0019233 | sensory perception of pain | 5 | -3.840767482 |
| GO Biological Processes | GO:0019395 | fatty acid oxidation | 5 | -3.840767482 |
| GO Biological Processes | GO:0030301 | cholesterol transport | 5 | -3.840767482 |
| GO Biological Processes | GO:0032928 | regulation of superoxide anion generation | 3 | -3.840120534 |
| GO Biological Processes | GO:0051000 | positive regulation of nitric-oxide synthase activity | 3 | -3.840120534 |
| GO Biological Processes | GO:0060352 | cell adhesion molecule production | 3 | -3.840120534 |
| GO Biological Processes | GO:1901685 | glutathione derivative metabolic process | 3 | -3.840120534 |
| GO Biological Processes | GO:1901687 | glutathione derivative biosynthetic process | 3 | -3.840120534 |
| GO Biological Processes | GO:1902884 | positive regulation of response to oxidative stress | 3 | -3.840120534 |
| GO Biological Processes | GO:0030520 | intracellular estrogen receptor signaling pathway | 4 | -3.825975891 |
| GO Biological Processes | GO:0048016 | inositol phosphate-mediated signaling | 4 | -3.825975891 |
| GO Biological Processes | GO:0071398 | cellular response to fatty acid | 4 | -3.825975891 |
| GO Biological Processes | GO:0008593 | regulation of Notch signaling pathway | 5 | -3.802634753 |
| GO Biological Processes | GO:0032526 | response to retinoic acid | 5 | -3.802634753 |
| GO Biological Processes | GO:0030307 | positive regulation of cell growth | 6 | -3.800494066 |
| GO Biological Processes | GO:0031294 | lymphocyte costimulation | 4 | -3.796510414 |
| GO Biological Processes | GO:1900408 | negative regulation of cellular response to oxidative stress | 4 | -3.796510414 |
| GO Biological Processes | GO:1903202 | negative regulation of oxidative stress-induced cell death | 4 | -3.796510414 |
| GO Biological Processes | GO:0021761 | limbic system development | 5 | -3.783860085 |
| GO Biological Processes | GO:0002218 | activation of innate immune response | 8 | -3.781612516 |
| GO Biological Processes | GO:0031639 | plasminogen activation | 3 | -3.780908985 |
| GO Biological Processes | GO:0045649 | regulation of macrophage differentiation | 3 | -3.780908985 |
| GO Biological Processes | GO:0051043 | regulation of membrane protein ectodomain proteolysis | 3 | -3.780908985 |
| GO Biological Processes | GO:2000810 | regulation of bicellular tight junction assembly | 3 | -3.780908985 |
| GO Biological Processes | GO:0031638 | zymogen activation | 4 | -3.767589644 |
| GO Biological Processes | GO:0042306 | regulation of protein import into nucleus | 4 | -3.767589644 |
| GO Biological Processes | GO:0045454 | cell redox homeostasis | 4 | -3.767589644 |
| GO Biological Processes | GO:0045981 | positive regulation of nucleotide metabolic process | 4 | -3.767589644 |
| GO Biological Processes | GO:0048008 | platelet-derived growth factor receptor signaling pathway | 4 | -3.767589644 |
| GO Biological Processes | GO:0060043 | regulation of cardiac muscle cell proliferation | 4 | -3.767589644 |
| GO Biological Processes | GO:1900544 | positive regulation of purine nucleotide metabolic process | 4 | -3.767589644 |
| GO Biological Processes | GO:0042303 | molting cycle | 5 | -3.746876137 |
| GO Biological Processes | GO:0042633 | hair cycle | 5 | -3.746876137 |
| GO Biological Processes | GO:0010921 | regulation of phosphatase activity | 6 | -3.745954897 |
| GO Biological Processes | GO:0045216 | cell-cell junction organization | 6 | -3.745954897 |
| GO Biological Processes | GO:0032732 | positive regulation of interleukin-1 production | 4 | -3.739194768 |
| GO Biological Processes | GO:1902808 | positive regulation of cell cycle G1/S phase transition | 4 | -3.739194768 |
| GO Biological Processes | GO:1902883 | negative regulation of response to oxidative stress | 4 | -3.739194768 |
| GO Biological Processes | GO:1990778 | protein localization to cell periphery | 8 | -3.737081597 |
| GO Biological Processes | GO:0032652 | regulation of interleukin-1 production | 5 | -3.728659954 |
| GO Biological Processes | GO:0042738 | exogenous drug catabolic process | 3 | -3.724403023 |
| GO Biological Processes | GO:0050995 | negative regulation of lipid catabolic process | 3 | -3.724403023 |
| GO Biological Processes | GO:0051195 | negative regulation of cofactor metabolic process | 3 | -3.724403023 |
| GO Biological Processes | GO:0060444 | branching involved in mammary gland duct morphogenesis | 3 | -3.724403023 |
| GO Biological Processes | GO:1901889 | negative regulation of cell junction assembly | 3 | -3.724403023 |
| GO Biological Processes | GO:0030324 | lung development | 6 | -3.719214449 |
| GO Biological Processes | GO:0043901 | negative regulation of multi-organism process | 6 | -3.719214449 |
| GO Biological Processes | GO:0033013 | tetrapyrrole metabolic process | 4 | -3.71130793 |
| GO Biological Processes | GO:0033619 | membrane protein proteolysis | 4 | -3.71130793 |
| GO Biological Processes | GO:0046324 | regulation of glucose import | 4 | -3.71130793 |
| GO Biological Processes | GO:1903078 | positive regulation of protein localization to plasma membrane | 4 | -3.71130793 |
| GO Biological Processes | GO:0062014 | negative regulation of small molecule metabolic process | 5 | -3.710623184 |
| GO Biological Processes | GO:0032411 | positive regulation of transporter activity | 5 | -3.692762605 |
| GO Biological Processes | GO:0046916 | cellular transition metal ion homeostasis | 5 | -3.692762605 |
| GO Biological Processes | GO:0009205 | purine ribonucleoside triphosphate metabolic process | 8 | -3.684690597 |
| GO Biological Processes | GO:0030888 | regulation of B cell proliferation | 4 | -3.68391217 |
| GO Biological Processes | GO:0055025 | positive regulation of cardiac muscle tissue development | 4 | -3.68391217 |
| GO Biological Processes | GO:1904589 | regulation of protein import | 4 | -3.68391217 |
| GO Biological Processes | GO:0002053 | positive regulation of mesenchymal cell proliferation | 3 | -3.670371374 |
| GO Biological Processes | GO:0045662 | negative regulation of myoblast differentiation | 3 | -3.670371374 |
| GO Biological Processes | GO:0060330 | regulation of response to interferon-gamma | 3 | -3.670371374 |
| GO Biological Processes | GO:0060334 | regulation of interferon-gamma-mediated signaling pathway | 3 | -3.670371374 |
| GO Biological Processes | GO:0060571 | morphogenesis of an epithelial fold | 3 | -3.670371374 |
| GO Biological Processes | GO:0060575 | intestinal epithelial cell differentiation | 3 | -3.670371374 |
| GO Biological Processes | GO:0071677 | positive regulation of mononuclear cell migration | 3 | -3.670371374 |
| GO Biological Processes | GO:2000679 | positive regulation of transcription regulatory region DNA binding | 3 | -3.670371374 |
| GO Biological Processes | GO:0030323 | respiratory tube development | 6 | -3.666751864 |
| GO Biological Processes | GO:0045619 | regulation of lymphocyte differentiation | 6 | -3.666751864 |
| GO Biological Processes | GO:0031623 | receptor internalization | 5 | -3.657557557 |
| GO Biological Processes | GO:0046888 | negative regulation of hormone secretion | 4 | -3.656991361 |
| GO Biological Processes | GO:0070059 | intrinsic apoptotic signaling pathway in response to endoplasmic reticulum stress | 4 | -3.656991361 |
| GO Biological Processes | GO:2001252 | positive regulation of chromosome organization | 6 | -3.653842808 |
| GO Biological Processes | GO:2001257 | regulation of cation channel activity | 6 | -3.653842808 |
| GO Biological Processes | GO:0043666 | regulation of phosphoprotein phosphatase activity | 5 | -3.640207061 |
| GO Biological Processes | GO:0072676 | lymphocyte migration | 5 | -3.640207061 |
| GO Biological Processes | GO:0009199 | ribonucleoside triphosphate metabolic process | 8 | -3.633402624 |
| GO Biological Processes | GO:0033344 | cholesterol efflux | 4 | -3.630530162 |
| GO Biological Processes | GO:0007265 | Ras protein signal transduction | 8 | -3.624959141 |
| GO Biological Processes | GO:0009144 | purine nucleoside triphosphate metabolic process | 8 | -3.624959141 |
| GO Biological Processes | GO:0015918 | sterol transport | 5 | -3.623020697 |
| GO Biological Processes | GO:0051209 | release of sequestered calcium ion into cytosol | 5 | -3.623020697 |
| GO Biological Processes | GO:0002092 | positive regulation of receptor internalization | 3 | -3.618611234 |
| GO Biological Processes | GO:0006706 | steroid catabolic process | 3 | -3.618611234 |
| GO Biological Processes | GO:0046434 | organophosphate catabolic process | 7 | -3.61673739 |
| GO Biological Processes | GO:0009167 | purine ribonucleoside monophosphate metabolic process | 8 | -3.616545054 |
| GO Biological Processes | GO:0051283 | negative regulation of sequestering of calcium ion | 5 | -3.605995641 |
| GO Biological Processes | GO:0051926 | negative regulation of calcium ion transport | 4 | -3.604513957 |
| GO Biological Processes | GO:0009126 | purine nucleoside monophosphate metabolic process | 8 | -3.59147747 |
| GO Biological Processes | GO:0045333 | cellular respiration | 6 | -3.590491766 |
| GO Biological Processes | GO:0032922 | circadian regulation of gene expression | 4 | -3.578928822 |
| GO Biological Processes | GO:0051282 | regulation of sequestering of calcium ion | 5 | -3.57241853 |
| GO Biological Processes | GO:0010971 | positive regulation of G2/M transition of mitotic cell cycle | 3 | -3.568943776 |
| GO Biological Processes | GO:0030218 | erythrocyte differentiation | 5 | -3.555861182 |
| GO Biological Processes | GO:0046626 | regulation of insulin receptor signaling pathway | 4 | -3.553761472 |
| GO Biological Processes | GO:0072678 | T cell migration | 4 | -3.553761472 |
| GO Biological Processes | GO:0051783 | regulation of nuclear division | 6 | -3.553405633 |
| GO Biological Processes | GO:0022408 | negative regulation of cell-cell adhesion | 6 | -3.541192726 |
| GO Biological Processes | GO:0016525 | negative regulation of angiogenesis | 6 | -3.52905307 |
| GO Biological Processes | GO:1904377 | positive regulation of protein localization to cell periphery | 4 | -3.52899923 |
| GO Biological Processes | GO:0007173 | epidermal growth factor receptor signaling pathway | 5 | -3.52319616 |
| GO Biological Processes | GO:0051208 | sequestering of calcium ion | 5 | -3.52319616 |
| GO Biological Processes | GO:0002360 | T cell lineage commitment | 3 | -3.521210511 |
| GO Biological Processes | GO:0071280 | cellular response to copper ion | 3 | -3.521210511 |
| GO Biological Processes | GO:0090200 | positive regulation of release of cytochrome c from mitochondria | 3 | -3.521210511 |
| GO Biological Processes | GO:1902932 | positive regulation of alcohol biosynthetic process | 3 | -3.521210511 |
| GO Biological Processes | GO:1903579 | negative regulation of ATP metabolic process | 3 | -3.521210511 |
| GO Biological Processes | GO:0019362 | pyridine nucleotide metabolic process | 6 | -3.516985892 |
| GO Biological Processes | GO:0046496 | nicotinamide nucleotide metabolic process | 6 | -3.516985892 |
| GO Biological Processes | GO:0001952 | regulation of cell-matrix adhesion | 5 | -3.507083578 |
| GO Biological Processes | GO:2000181 | negative regulation of blood vessel morphogenesis | 6 | -3.50499043 |
| GO Biological Processes | GO:0060038 | cardiac muscle cell proliferation | 4 | -3.504629985 |
| GO Biological Processes | GO:0009161 | ribonucleoside monophosphate metabolic process | 8 | -3.501741394 |
| GO Biological Processes | GO:0055007 | cardiac muscle cell differentiation | 5 | -3.491114443 |
| GO Biological Processes | GO:0006260 | DNA replication | 7 | -3.489562976 |
| GO Biological Processes | GO:0007178 | transmembrane receptor protein serine/threonine kinase signaling pathway | 8 | -3.485782536 |
| GO Biological Processes | GO:0071242 | cellular response to ammonium ion | 4 | -3.480642163 |
| GO Biological Processes | GO:0045165 | cell fate commitment | 7 | -3.480076262 |
| GO Biological Processes | GO:0019079 | viral genome replication | 5 | -3.475286446 |
| GO Biological Processes | GO:0046031 | ADP metabolic process | 5 | -3.475286446 |
| GO Biological Processes | GO:0008209 | androgen metabolic process | 3 | -3.475270311 |
| GO Biological Processes | GO:0015721 | bile acid and bile salt transport | 3 | -3.475270311 |
| GO Biological Processes | GO:0031063 | regulation of histone deacetylation | 3 | -3.475270311 |
| GO Biological Processes | GO:0032373 | positive regulation of sterol transport | 3 | -3.475270311 |
| GO Biological Processes | GO:0032376 | positive regulation of cholesterol transport | 3 | -3.475270311 |
| GO Biological Processes | GO:0050901 | leukocyte tethering or rolling | 3 | -3.475270311 |
| GO Biological Processes | GO:0009141 | nucleoside triphosphate metabolic process | 8 | -3.469930408 |
| GO Biological Processes | GO:1903052 | positive regulation of proteolysis involved in cellular protein catabolic process | 5 | -3.459597336 |
| GO Biological Processes | GO:0042982 | amyloid precursor protein metabolic process | 4 | -3.457024691 |
| GO Biological Processes | GO:2001259 | positive regulation of cation channel activity | 4 | -3.457024691 |
| GO Biological Processes | GO:1902275 | regulation of chromatin organization | 6 | -3.446063092 |
| GO Biological Processes | GO:0010256 | endomembrane system organization | 9 | -3.445744472 |
| GO Biological Processes | GO:0002576 | platelet degranulation | 5 | -3.444044913 |
| GO Biological Processes | GO:0031330 | negative regulation of cellular catabolic process | 7 | -3.44253366 |
| GO Biological Processes | GO:0071897 | DNA biosynthetic process | 6 | -3.434482616 |
| GO Biological Processes | GO:0072524 | pyridine-containing compound metabolic process | 6 | -3.434482616 |
| GO Biological Processes | GO:0042531 | positive regulation of tyrosine phosphorylation of STAT protein | 4 | -3.433766973 |
| GO Biological Processes | GO:0045600 | positive regulation of fat cell differentiation | 4 | -3.433766973 |
| GO Biological Processes | GO:0035666 | TRIF-dependent toll-like receptor signaling pathway | 3 | -3.430996962 |
| GO Biological Processes | GO:1902751 | positive regulation of cell cycle G2/M phase transition | 3 | -3.430996962 |
| GO Biological Processes | GO:0006790 | sulfur compound metabolic process | 8 | -3.430759354 |
| GO Biological Processes | GO:0072659 | protein localization to plasma membrane | 7 | -3.414793025 |
| GO Biological Processes | GO:0019218 | regulation of steroid metabolic process | 5 | -3.413341588 |
| GO Biological Processes | GO:0060047 | heart contraction | 7 | -3.396493266 |
| GO Biological Processes | GO:0007423 | sensory organ development | 10 | -3.394887226 |
| GO Biological Processes | GO:0060541 | respiratory system development | 6 | -3.38882119 |
| GO Biological Processes | GO:0006635 | fatty acid beta-oxidation | 4 | -3.388290631 |
| GO Biological Processes | GO:0045940 | positive regulation of steroid metabolic process | 3 | -3.388277133 |
| GO Biological Processes | GO:1900543 | negative regulation of purine nucleotide metabolic process | 3 | -3.388277133 |
| GO Biological Processes | GO:1900745 | positive regulation of p38MAPK cascade | 3 | -3.388277133 |
| GO Biological Processes | GO:0035924 | cellular response to vascular endothelial growth factor stimulus | 4 | -3.366052957 |
| GO Biological Processes | GO:0050805 | negative regulation of synaptic transmission | 4 | -3.366052957 |
| GO Biological Processes | GO:0072088 | nephron epithelium morphogenesis | 4 | -3.366052957 |
| GO Biological Processes | GO:1901343 | negative regulation of vasculature development | 6 | -3.355250361 |
| GO Biological Processes | GO:0046718 | viral entry into host cell | 5 | -3.353483972 |
| GO Biological Processes | GO:0046887 | positive regulation of hormone secretion | 5 | -3.353483972 |
| GO Biological Processes | GO:0010743 | regulation of macrophage derived foam cell differentiation | 3 | -3.347008678 |
| GO Biological Processes | GO:0042744 | hydrogen peroxide catabolic process | 3 | -3.347008678 |
| GO Biological Processes | GO:0045980 | negative regulation of nucleotide metabolic process | 3 | -3.347008678 |
| GO Biological Processes | GO:0060603 | mammary gland duct morphogenesis | 3 | -3.347008678 |
| GO Biological Processes | GO:0097421 | liver regeneration | 3 | -3.347008678 |
| GO Biological Processes | GO:0098810 | neurotransmitter reuptake | 3 | -3.347008678 |
| GO Biological Processes | GO:0009123 | nucleoside monophosphate metabolic process | 8 | -3.346818317 |
| GO Biological Processes | GO:0008306 | associative learning | 4 | -3.344136893 |
| GO Biological Processes | GO:0072091 | regulation of stem cell proliferation | 4 | -3.344136893 |
| GO Biological Processes | GO:1900076 | regulation of cellular response to insulin stimulus | 4 | -3.344136893 |
| GO Biological Processes | GO:0055076 | transition metal ion homeostasis | 5 | -3.338830934 |
| GO Biological Processes | GO:0035303 | regulation of dephosphorylation | 6 | -3.333181929 |
| GO Biological Processes | GO:0051048 | negative regulation of secretion | 6 | -3.333181929 |
| GO Biological Processes | GO:0001654 | eye development | 8 | -3.331876 |
| GO Biological Processes | GO:0030183 | B cell differentiation | 5 | -3.324298725 |
| GO Biological Processes | GO:1903038 | negative regulation of leukocyte cell-cell adhesion | 5 | -3.324298725 |
| GO Biological Processes | GO:0061418 | regulation of transcription from RNA polymerase II promoter in response to hypoxia | 4 | -3.322533851 |
| GO Biological Processes | GO:0072028 | nephron morphogenesis | 4 | -3.322533851 |
| GO Biological Processes | GO:0009165 | nucleotide biosynthetic process | 8 | -3.30964179 |
| GO Biological Processes | GO:0003015 | heart process | 7 | -3.307241659 |
| GO Biological Processes | GO:0010464 | regulation of mesenchymal cell proliferation | 3 | -3.307099209 |
| GO Biological Processes | GO:0042573 | retinoic acid metabolic process | 3 | -3.307099209 |
| GO Biological Processes | GO:0050849 | negative regulation of calcium-mediated signaling | 3 | -3.307099209 |
| GO Biological Processes | GO:0045089 | positive regulation of innate immune response | 8 | -3.30227769 |
| GO Biological Processes | GO:0150063 | visual system development | 8 | -3.30227769 |
| GO Biological Processes | GO:0007272 | ensheathment of neurons | 5 | -3.295589689 |
| GO Biological Processes | GO:0008366 | axon ensheathment | 5 | -3.295589689 |
| GO Biological Processes | GO:0009135 | purine nucleoside diphosphate metabolic process | 5 | -3.295589689 |
| GO Biological Processes | GO:0009179 | purine ribonucleoside diphosphate metabolic process | 5 | -3.295589689 |
| GO Biological Processes | GO:0035304 | regulation of protein dephosphorylation | 5 | -3.295589689 |
| GO Biological Processes | GO:0006733 | oxidoreduction coenzyme metabolic process | 6 | -3.289772592 |
| GO Biological Processes | GO:1901293 | nucleoside phosphate biosynthetic process | 8 | -3.287619707 |
| GO Biological Processes | GO:0097553 | calcium ion transmembrane import into cytosol | 5 | -3.281409406 |
| GO Biological Processes | GO:0042246 | tissue regeneration | 4 | -3.280234157 |
| GO Biological Processes | GO:0016311 | dephosphorylation | 9 | -3.275816539 |
| GO Biological Processes | GO:0002756 | MyD88-independent toll-like receptor signaling pathway | 3 | -3.268464892 |
| GO Biological Processes | GO:0032148 | activation of protein kinase B activity | 3 | -3.268464892 |
| GO Biological Processes | GO:0033198 | response to ATP | 3 | -3.268464892 |
| GO Biological Processes | GO:0035633 | maintenance of permeability of blood-brain barrier | 3 | -3.268464892 |
| GO Biological Processes | GO:0046627 | negative regulation of insulin receptor signaling pathway | 3 | -3.268464892 |
| GO Biological Processes | GO:1902692 | regulation of neuroblast proliferation | 3 | -3.268464892 |
| GO Biological Processes | GO:2000300 | regulation of synaptic vesicle exocytosis | 4 | -3.259521956 |
| GO Biological Processes | GO:0048880 | sensory system development | 8 | -3.258581229 |
| GO Biological Processes | GO:0009185 | ribonucleoside diphosphate metabolic process | 5 | -3.253388948 |
| GO Biological Processes | GO:0072073 | kidney epithelium development | 5 | -3.253388948 |
| GO Biological Processes | GO:0050792 | regulation of viral process | 6 | -3.247302815 |
| GO Biological Processes | GO:1902749 | regulation of cell cycle G2/M phase transition | 6 | -3.247302815 |
| GO Biological Processes | GO:0048667 | cell morphogenesis involved in neuron differentiation | 10 | -3.246600451 |
| GO Biological Processes | GO:0009117 | nucleotide metabolic process | 11 | -3.246169706 |
| GO Biological Processes | GO:0021766 | hippocampus development | 4 | -3.239091646 |
| GO Biological Processes | GO:0030947 | regulation of vascular endothelial growth factor receptor signaling pathway | 3 | -3.231029424 |
| GO Biological Processes | GO:0051194 | positive regulation of cofactor metabolic process | 3 | -3.231029424 |
| GO Biological Processes | GO:0070884 | regulation of calcineurin-NFAT signaling cascade | 3 | -3.231029424 |
| GO Biological Processes | GO:0110111 | negative regulation of animal organ morphogenesis | 3 | -3.231029424 |
| GO Biological Processes | GO:0070374 | positive regulation of ERK1 and ERK2 cascade | 6 | -3.22640931 |
| GO Biological Processes | GO:0060078 | regulation of postsynaptic membrane potential | 5 | -3.225811234 |
| GO Biological Processes | GO:0006753 | nucleoside phosphate metabolic process | 11 | -3.211387376 |
| GO Biological Processes | GO:0030811 | regulation of nucleotide catabolic process | 4 | -3.199048731 |
| GO Biological Processes | GO:0071277 | cellular response to calcium ion | 4 | -3.199048731 |
| GO Biological Processes | GO:0044344 | cellular response to fibroblast growth factor stimulus | 5 | -3.198663858 |
| GO Biological Processes | GO:0071322 | cellular response to carbohydrate stimulus | 5 | -3.198663858 |
| GO Biological Processes | GO:0010543 | regulation of platelet activation | 3 | -3.19472315 |
| GO Biological Processes | GO:0033280 | response to vitamin D | 3 | -3.19472315 |
| GO Biological Processes | GO:0106056 | regulation of calcineurin-mediated signaling | 3 | -3.19472315 |
| GO Biological Processes | GO:1900077 | negative regulation of cellular response to insulin stimulus | 3 | -3.19472315 |
| GO Biological Processes | GO:2000352 | negative regulation of endothelial cell apoptotic process | 3 | -3.19472315 |
| GO Biological Processes | GO:0019359 | nicotinamide nucleotide biosynthetic process | 5 | -3.185247811 |
| GO Biological Processes | GO:0019363 | pyridine nucleotide biosynthetic process | 5 | -3.185247811 |
| GO Biological Processes | GO:0051588 | regulation of neurotransmitter transport | 5 | -3.185247811 |
| GO Biological Processes | GO:0015844 | monoamine transport | 4 | -3.179422784 |
| GO Biological Processes | GO:0090287 | regulation of cellular response to growth factor stimulus | 7 | -3.171746684 |
| GO Biological Processes | GO:0007264 | small GTPase mediated signal transduction | 9 | -3.164736434 |
| GO Biological Processes | GO:0110110 | positive regulation of animal organ morphogenesis | 4 | -3.160052019 |
| GO Biological Processes | GO:0070423 | nucleotide-binding oligomerization domain containing signaling pathway | 3 | -3.159482321 |
| GO Biological Processes | GO:0071542 | dopaminergic neuron differentiation | 3 | -3.159482321 |
| GO Biological Processes | GO:0090050 | positive regulation of cell migration involved in sprouting angiogenesis | 3 | -3.159482321 |
| GO Biological Processes | GO:0017157 | regulation of exocytosis | 6 | -3.155003884 |
| GO Biological Processes | GO:0072521 | purine-containing compound metabolic process | 10 | -3.148760696 |
| GO Biological Processes | GO:0072525 | pyridine-containing compound biosynthetic process | 5 | -3.145613033 |
| GO Biological Processes | GO:1903364 | positive regulation of cellular protein catabolic process | 5 | -3.145613033 |
| GO Biological Processes | GO:0070664 | negative regulation of leukocyte proliferation | 4 | -3.14093035 |
| GO Biological Processes | GO:1902803 | regulation of synaptic vesicle transport | 4 | -3.14093035 |
| GO Biological Processes | GO:1903169 | regulation of calcium ion transmembrane transport | 5 | -3.132601287 |
| GO Biological Processes | GO:0010661 | positive regulation of muscle cell apoptotic process | 3 | -3.125248435 |
| GO Biological Processes | GO:0035872 | nucleotide-binding domain, leucine rich repeat containing receptor signaling pathway | 3 | -3.125248435 |
| GO Biological Processes | GO:0048009 | insulin-like growth factor receptor signaling pathway | 3 | -3.125248435 |
| GO Biological Processes | GO:0060045 | positive regulation of cardiac muscle cell proliferation | 3 | -3.125248435 |
| GO Biological Processes | GO:0071392 | cellular response to estradiol stimulus | 3 | -3.125248435 |
| GO Biological Processes | GO:1905898 | positive regulation of response to endoplasmic reticulum stress | 3 | -3.125248435 |
| GO Biological Processes | GO:2000144 | positive regulation of DNA-templated transcription, initiation | 3 | -3.125248435 |
| GO Biological Processes | GO:1905114 | cell surface receptor signaling pathway involved in cell-cell signaling | 10 | -3.122245639 |
| GO Biological Processes | GO:0001942 | hair follicle development | 4 | -3.122051908 |
| GO Biological Processes | GO:0061351 | neural precursor cell proliferation | 5 | -3.119687228 |
| GO Biological Processes | GO:0014068 | positive regulation of phosphatidylinositol 3-kinase signaling | 4 | -3.103411028 |
| GO Biological Processes | GO:0032436 | positive regulation of proteasomal ubiquitin-dependent protein catabolic process | 4 | -3.103411028 |
| GO Biological Processes | GO:0032755 | positive regulation of interleukin-6 production | 4 | -3.103411028 |
| GO Biological Processes | GO:0001678 | cellular glucose homeostasis | 5 | -3.094146999 |
| GO Biological Processes | GO:0006778 | porphyrin-containing compound metabolic process | 3 | -3.091967685 |
| GO Biological Processes | GO:0033146 | regulation of intracellular estrogen receptor signaling pathway | 3 | -3.091967685 |
| GO Biological Processes | GO:0022404 | molting cycle process | 4 | -3.085002242 |
| GO Biological Processes | GO:0022405 | hair cycle process | 4 | -3.085002242 |
| GO Biological Processes | GO:0006090 | pyruvate metabolic process | 5 | -3.081518303 |
| GO Biological Processes | GO:0051053 | negative regulation of DNA metabolic process | 5 | -3.081518303 |
| GO Biological Processes | GO:0006470 | protein dephosphorylation | 7 | -3.07554922 |
| GO Biological Processes | GO:0043470 | regulation of carbohydrate catabolic process | 4 | -3.066820267 |
| GO Biological Processes | GO:0098773 | skin epidermis development | 4 | -3.066820267 |
| GO Biological Processes | GO:0050851 | antigen receptor-mediated signaling pathway | 7 | -3.059939781 |
| GO Biological Processes | GO:0001656 | metanephros development | 4 | -3.048859997 |
| GO Biological Processes | GO:0051899 | membrane depolarization | 4 | -3.048859997 |
| GO Biological Processes | GO:0044242 | cellular lipid catabolic process | 6 | -3.04789287 |
| GO Biological Processes | GO:0061564 | axon development | 9 | -3.047088244 |
| GO Biological Processes | GO:0043161 | proteasome-mediated ubiquitin-dependent protein catabolic process | 8 | -3.045325683 |
| GO Biological Processes | GO:0043010 | camera-type eye development | 7 | -3.044447314 |
| GO Biological Processes | GO:0007519 | skeletal muscle tissue development | 5 | -3.044183257 |
| GO Biological Processes | GO:0009132 | nucleoside diphosphate metabolic process | 5 | -3.044183257 |
| GO Biological Processes | GO:0050777 | negative regulation of immune response | 5 | -3.031917993 |
| GO Biological Processes | GO:0031058 | positive regulation of histone modification | 4 | -3.0311165 |
| GO Biological Processes | GO:0045582 | positive regulation of T cell differentiation | 4 | -3.0311165 |
| GO Biological Processes | GO:1903321 | negative regulation of protein modification by small protein conjugation or removal | 4 | -3.0311165 |
| GO Biological Processes | GO:0007618 | mating | 3 | -3.028070937 |
| GO Biological Processes | GO:0033574 | response to testosterone | 3 | -3.028070937 |
| GO Biological Processes | GO:0038083 | peptidyl-tyrosine autophosphorylation | 3 | -3.028070937 |
| GO Biological Processes | GO:0046825 | regulation of protein export from nucleus | 3 | -3.028070937 |
| GO Biological Processes | GO:1900117 | regulation of execution phase of apoptosis | 3 | -3.028070937 |
| GO Biological Processes | GO:2000008 | regulation of protein localization to cell surface | 3 | -3.028070937 |
| GO Biological Processes | GO:0032088 | negative regulation of NF-kappaB transcription factor activity | 4 | -3.013585002 |
| GO Biological Processes | GO:0072080 | nephron tubule development | 4 | -3.013585002 |
| GO Biological Processes | GO:2000058 | regulation of ubiquitin-dependent protein catabolic process | 5 | -3.007650255 |
| GO Biological Processes | GO:1903320 | regulation of protein modification by small protein conjugation or removal | 6 | -3.001131349 |
| GO Biological Processes | GO:0009268 | response to pH | 3 | -2.997366675 |
| GO Biological Processes | GO:0010907 | positive regulation of glucose metabolic process | 3 | -2.997366675 |
| GO Biological Processes | GO:0046621 | negative regulation of organ growth | 3 | -2.997366675 |
| GO Biological Processes | GO:0045778 | positive regulation of ossification | 4 | -2.99626089 |
| GO Biological Processes | GO:0006754 | ATP biosynthetic process | 5 | -2.995645577 |
| GO Biological Processes | GO:0035821 | modification of morphology or physiology of other organism | 5 | -2.995645577 |
| GO Biological Processes | GO:0006898 | receptor-mediated endocytosis | 7 | -2.983618669 |
| GO Biological Processes | GO:0003073 | regulation of systemic arterial blood pressure | 4 | -2.979139695 |
| GO Biological Processes | GO:0060993 | kidney morphogenesis | 4 | -2.979139695 |
| GO Biological Processes | GO:0061326 | renal tubule development | 4 | -2.979139695 |
| GO Biological Processes | GO:0046006 | regulation of activated T cell proliferation | 3 | -2.967438321 |
| GO Biological Processes | GO:0071470 | cellular response to osmotic stress | 3 | -2.967438321 |
| GO Biological Processes | GO:1903146 | regulation of autophagy of mitochondrion | 3 | -2.967438321 |
| GO Biological Processes | GO:2000648 | positive regulation of stem cell proliferation | 3 | -2.967438321 |
| GO Biological Processes | GO:0051196 | regulation of coenzyme metabolic process | 4 | -2.962217094 |
| GO Biological Processes | GO:1903305 | regulation of regulated secretory pathway | 5 | -2.960135515 |
| GO Biological Processes | GO:0009150 | purine ribonucleotide metabolic process | 9 | -2.951536489 |
| GO Biological Processes | GO:0010463 | mesenchymal cell proliferation | 3 | -2.938249299 |
| GO Biological Processes | GO:0042088 | T-helper 1 type immune response | 3 | -2.938249299 |
| GO Biological Processes | GO:1903053 | regulation of extracellular matrix organization | 3 | -2.938249299 |
| GO Biological Processes | GO:0001657 | ureteric bud development | 4 | -2.928951054 |
| GO Biological Processes | GO:0006476 | protein deacetylation | 4 | -2.928951054 |
| GO Biological Processes | GO:0045833 | negative regulation of lipid metabolic process | 4 | -2.928951054 |
| GO Biological Processes | GO:2001169 | regulation of ATP biosynthetic process | 4 | -2.928951054 |
| GO Biological Processes | GO:0060538 | skeletal muscle organ development | 5 | -2.925359937 |
| GO Biological Processes | GO:0048812 | neuron projection morphogenesis | 10 | -2.919823983 |
| GO Biological Processes | GO:0072163 | mesonephric epithelium development | 4 | -2.912599628 |
| GO Biological Processes | GO:0072164 | mesonephric tubule development | 4 | -2.912599628 |
| GO Biological Processes | GO:0090277 | positive regulation of peptide hormone secretion | 4 | -2.912599628 |
| GO Biological Processes | GO:0006509 | membrane protein ectodomain proteolysis | 3 | -2.909765561 |
| GO Biological Processes | GO:0042771 | intrinsic apoptotic signaling pathway in response to DNA damage by p53 class mediator | 3 | -2.909765561 |
| GO Biological Processes | GO:0032874 | positive regulation of stress-activated MAPK cascade | 5 | -2.902571183 |
| GO Biological Processes | GO:0045069 | regulation of viral genome replication | 4 | -2.896430809 |
| GO Biological Processes | GO:0050848 | regulation of calcium-mediated signaling | 4 | -2.896430809 |
| GO Biological Processes | GO:0010863 | positive regulation of phospholipase C activity | 3 | -2.881955357 |
| GO Biological Processes | GO:0043330 | response to exogenous dsRNA | 3 | -2.881955357 |
| GO Biological Processes | GO:2000142 | regulation of DNA-templated transcription, initiation | 3 | -2.881955357 |
| GO Biological Processes | GO:0042116 | macrophage activation | 4 | -2.880440903 |
| GO Biological Processes | GO:0070304 | positive regulation of stress-activated protein kinase signaling cascade | 5 | -2.880089652 |
| GO Biological Processes | GO:0009259 | ribonucleotide metabolic process | 9 | -2.870406226 |
| GO Biological Processes | GO:0009206 | purine ribonucleoside triphosphate biosynthetic process | 5 | -2.868961833 |
| GO Biological Processes | GO:0048525 | negative regulation of viral process | 4 | -2.864626325 |
| GO Biological Processes | GO:0009145 | purine nucleoside triphosphate biosynthetic process | 5 | -2.857908138 |
| GO Biological Processes | GO:0044282 | small molecule catabolic process | 8 | -2.856865015 |
| GO Biological Processes | GO:0007157 | heterophilic cell-cell adhesion via plasma membrane cell adhesion molecules | 3 | -2.854789034 |
| GO Biological Processes | GO:0008542 | visual learning | 3 | -2.854789034 |
| GO Biological Processes | GO:0042551 | neuron maturation | 3 | -2.854789034 |
| GO Biological Processes | GO:0045776 | negative regulation of blood pressure | 3 | -2.854789034 |
| GO Biological Processes | GO:0048806 | genitalia development | 3 | -2.854789034 |
| GO Biological Processes | GO:0120039 | plasma membrane bounded cell projection morphogenesis | 10 | -2.85280804 |
| GO Biological Processes | GO:0003007 | heart morphogenesis | 6 | -2.850349899 |
| GO Biological Processes | GO:0009108 | coenzyme biosynthetic process | 6 | -2.850349899 |
| GO Biological Processes | GO:0001823 | mesonephros development | 4 | -2.848983598 |
| GO Biological Processes | GO:0002042 | cell migration involved in sprouting angiogenesis | 4 | -2.848983598 |
| GO Biological Processes | GO:0048709 | oligodendrocyte differentiation | 4 | -2.848983598 |
| GO Biological Processes | GO:0060079 | excitatory postsynaptic potential | 4 | -2.848983598 |
| GO Biological Processes | GO:0006941 | striated muscle contraction | 5 | -2.846927706 |
| GO Biological Processes | GO:0048858 | cell projection morphogenesis | 10 | -2.834004487 |
| GO Biological Processes | GO:0031952 | regulation of protein autophosphorylation | 3 | -2.828238856 |
| GO Biological Processes | GO:0042220 | response to cocaine | 3 | -2.828238856 |
| GO Biological Processes | GO:0050798 | activated T cell proliferation | 3 | -2.828238856 |
| GO Biological Processes | GO:1900744 | regulation of p38MAPK cascade | 3 | -2.828238856 |
| GO Biological Processes | GO:0044070 | regulation of anion transport | 4 | -2.818200293 |
| GO Biological Processes | GO:0045621 | positive regulation of lymphocyte differentiation | 4 | -2.818200293 |
| GO Biological Processes | GO:0009201 | ribonucleoside triphosphate biosynthetic process | 5 | -2.80372188 |
| GO Biological Processes | GO:0032414 | positive regulation of ion transmembrane transporter activity | 4 | -2.803053256 |
| GO Biological Processes | GO:0046928 | regulation of neurotransmitter secretion | 4 | -2.803053256 |
| GO Biological Processes | GO:1905269 | positive regulation of chromatin organization | 4 | -2.803053256 |
| GO Biological Processes | GO:1900274 | regulation of phospholipase C activity | 3 | -2.802278839 |
| GO Biological Processes | GO:1990090 | cellular response to nerve growth factor stimulus | 3 | -2.802278839 |
| GO Biological Processes | GO:0032412 | regulation of ion transmembrane transporter activity | 6 | -2.799941335 |
| GO Biological Processes | GO:0048863 | stem cell differentiation | 6 | -2.799941335 |
| GO Biological Processes | GO:0090407 | organophosphate biosynthetic process | 10 | -2.783060417 |
| GO Biological Processes | GO:0001774 | microglial cell activation | 3 | -2.77688461 |
| GO Biological Processes | GO:0002269 | leukocyte activation involved in inflammatory response | 3 | -2.77688461 |
| GO Biological Processes | GO:0010718 | positive regulation of epithelial to mesenchymal transition | 3 | -2.77688461 |
| GO Biological Processes | GO:0048546 | digestive tract morphogenesis | 3 | -2.77688461 |
| GO Biological Processes | GO:0071715 | icosanoid transport | 3 | -2.77688461 |
| GO Biological Processes | GO:1901571 | fatty acid derivative transport | 3 | -2.77688461 |
| GO Biological Processes | GO:0035601 | protein deacylation | 4 | -2.773232951 |
| GO Biological Processes | GO:0008361 | regulation of cell size | 5 | -2.772046693 |
| GO Biological Processes | GO:0090596 | sensory organ morphogenesis | 6 | -2.767093759 |
| GO Biological Processes | GO:0019693 | ribose phosphate metabolic process | 9 | -2.766586179 |
| GO Biological Processes | GO:0002286 | T cell activation involved in immune response | 4 | -2.758553759 |
| GO Biological Processes | GO:0018958 | phenol-containing compound metabolic process | 4 | -2.758553759 |
| GO Biological Processes | GO:0001504 | neurotransmitter uptake | 3 | -2.752033274 |
| GO Biological Processes | GO:0032873 | negative regulation of stress-activated MAPK cascade | 3 | -2.752033274 |
| GO Biological Processes | GO:0035272 | exocrine system development | 3 | -2.752033274 |
| GO Biological Processes | GO:0045912 | negative regulation of carbohydrate metabolic process | 3 | -2.752033274 |
| GO Biological Processes | GO:0070303 | negative regulation of stress-activated protein kinase signaling cascade | 3 | -2.752033274 |
| GO Biological Processes | GO:1903573 | negative regulation of response to endoplasmic reticulum stress | 3 | -2.752033274 |
| GO Biological Processes | GO:0022900 | electron transport chain | 5 | -2.751265568 |
| GO Biological Processes | GO:1903531 | negative regulation of secretion by cell | 5 | -2.751265568 |
| GO Biological Processes | GO:0002821 | positive regulation of adaptive immune response | 4 | -2.74402473 |
| GO Biological Processes | GO:0072009 | nephron epithelium development | 4 | -2.74402473 |
| GO Biological Processes | GO:1901800 | positive regulation of proteasomal protein catabolic process | 4 | -2.74402473 |
| GO Biological Processes | GO:0009168 | purine ribonucleoside monophosphate biosynthetic process | 5 | -2.740973488 |
| GO Biological Processes | GO:0006163 | purine nucleotide metabolic process | 9 | -2.736364251 |
| GO Biological Processes | GO:0044839 | cell cycle G2/M phase transition | 6 | -2.734831517 |
| GO Biological Processes | GO:0009166 | nucleotide catabolic process | 5 | -2.730746106 |
| GO Biological Processes | GO:0043488 | regulation of mRNA stability | 5 | -2.730746106 |
| GO Biological Processes | GO:0048259 | regulation of receptor-mediated endocytosis | 4 | -2.729643102 |
| GO Biological Processes | GO:1903008 | organelle disassembly | 4 | -2.729643102 |
| GO Biological Processes | GO:0008544 | epidermis development | 8 | -2.728507245 |
| GO Biological Processes | GO:0031103 | axon regeneration | 3 | -2.727703298 |
| GO Biological Processes | GO:0071622 | regulation of granulocyte chemotaxis | 3 | -2.727703298 |
| GO Biological Processes | GO:0101023 | vascular endothelial cell proliferation | 3 | -2.727703298 |
| GO Biological Processes | GO:1905562 | regulation of vascular endothelial cell proliferation | 3 | -2.727703298 |
| GO Biological Processes | GO:1990089 | response to nerve growth factor | 3 | -2.727703298 |
| GO Biological Processes | GO:2000677 | regulation of transcription regulatory region DNA binding | 3 | -2.727703298 |
| GO Biological Processes | GO:0009127 | purine nucleoside monophosphate biosynthetic process | 5 | -2.720582719 |
| GO Biological Processes | GO:0043409 | negative regulation of MAPK cascade | 5 | -2.720582719 |
| GO Biological Processes | GO:0007281 | germ cell development | 6 | -2.718914475 |
| GO Biological Processes | GO:0098732 | macromolecule deacylation | 4 | -2.71540619 |
| GO Biological Processes | GO:0099565 | chemical synaptic transmission, postsynaptic | 4 | -2.71540619 |
| GO Biological Processes | GO:0002285 | lymphocyte activation involved in immune response | 5 | -2.710482634 |
| GO Biological Processes | GO:0051147 | regulation of muscle cell differentiation | 5 | -2.710482634 |
| GO Biological Processes | GO:0002429 | immune response-activating cell surface receptor signaling pathway | 8 | -2.705993183 |
| GO Biological Processes | GO:0043087 | regulation of GTPase activity | 8 | -2.705993183 |
| GO Biological Processes | GO:0007632 | visual behavior | 3 | -2.703874406 |
| GO Biological Processes | GO:0032964 | collagen biosynthetic process | 3 | -2.703874406 |
| GO Biological Processes | GO:0038066 | p38MAPK cascade | 3 | -2.703874406 |
| GO Biological Processes | GO:0043331 | response to dsRNA | 3 | -2.703874406 |
| GO Biological Processes | GO:2001258 | negative regulation of cation channel activity | 3 | -2.703874406 |
| GO Biological Processes | GO:0002223 | stimulatory C-type lectin receptor signaling pathway | 4 | -2.701311383 |
| GO Biological Processes | GO:0017158 | regulation of calcium ion-dependent exocytosis | 4 | -2.701311383 |
| GO Biological Processes | GO:1903510 | mucopolysaccharide metabolic process | 4 | -2.701311383 |
| GO Biological Processes | GO:0010498 | proteasomal protein catabolic process | 8 | -2.694827451 |
| GO Biological Processes | GO:0009142 | nucleoside triphosphate biosynthetic process | 5 | -2.690469659 |
| GO Biological Processes | GO:0009062 | fatty acid catabolic process | 4 | -2.687356138 |
| GO Biological Processes | GO:0022037 | metencephalon development | 4 | -2.687356138 |
| GO Biological Processes | GO:0098693 | regulation of synaptic vesicle cycle | 4 | -2.687356138 |
| GO Biological Processes | GO:1901185 | negative regulation of ERBB signaling pathway | 3 | -2.68052748 |
| GO Biological Processes | GO:2000179 | positive regulation of neural precursor cell proliferation | 3 | -2.68052748 |
| GO Biological Processes | GO:0006096 | glycolytic process | 4 | -2.673537979 |
| GO Biological Processes | GO:0007009 | plasma membrane organization | 4 | -2.673537979 |
| GO Biological Processes | GO:0045666 | positive regulation of neuron differentiation | 7 | -2.672040107 |
| GO Biological Processes | GO:0009152 | purine ribonucleotide biosynthetic process | 6 | -2.671994778 |
| GO Biological Processes | GO:0140014 | mitotic nuclear division | 6 | -2.671994778 |
| GO Biological Processes | GO:0044272 | sulfur compound biosynthetic process | 5 | -2.670701865 |
| GO Biological Processes | GO:1901292 | nucleoside phosphate catabolic process | 5 | -2.670701865 |
| GO Biological Processes | GO:0120035 | regulation of plasma membrane bounded cell projection organization | 10 | -2.666938182 |
| GO Biological Processes | GO:0006836 | neurotransmitter transport | 6 | -2.664293452 |
| GO Biological Processes | GO:0002220 | innate immune response activating cell surface receptor signaling pathway | 4 | -2.659854496 |
| GO Biological Processes | GO:0006757 | ATP generation from ADP | 4 | -2.659854496 |
| GO Biological Processes | GO:0006909 | phagocytosis | 7 | -2.659277566 |
| GO Biological Processes | GO:0050879 | multicellular organismal movement | 3 | -2.657644476 |
| GO Biological Processes | GO:0050881 | musculoskeletal movement | 3 | -2.657644476 |
| GO Biological Processes | GO:0030282 | bone mineralization | 4 | -2.646303344 |
| GO Biological Processes | GO:0043487 | regulation of RNA stability | 5 | -2.641498665 |
| GO Biological Processes | GO:0070228 | regulation of lymphocyte apoptotic process | 3 | -2.635208344 |
| GO Biological Processes | GO:0031344 | regulation of cell projection organization | 10 | -2.623811189 |
| GO Biological Processes | GO:0014902 | myotube differentiation | 4 | -2.619588942 |
| GO Biological Processes | GO:0042866 | pyruvate biosynthetic process | 4 | -2.619588942 |
| GO Biological Processes | GO:0031529 | ruffle organization | 3 | -2.613202957 |
| GO Biological Processes | GO:0051603 | proteolysis involved in cellular protein catabolic process | 10 | -2.606790404 |
| GO Biological Processes | GO:1900371 | regulation of purine nucleotide biosynthetic process | 4 | -2.606421294 |
| GO Biological Processes | GO:0002040 | sprouting angiogenesis | 5 | -2.603372452 |
| GO Biological Processes | GO:0007626 | locomotory behavior | 5 | -2.603372452 |
| GO Biological Processes | GO:0009156 | ribonucleoside monophosphate biosynthetic process | 5 | -2.603372452 |
| GO Biological Processes | GO:0051640 | organelle localization | 10 | -2.598328575 |
| GO Biological Processes | GO:0010389 | regulation of G2/M transition of mitotic cell cycle | 5 | -2.593981851 |
| GO Biological Processes | GO:0016052 | carbohydrate catabolic process | 5 | -2.593981851 |
| GO Biological Processes | GO:0007613 | memory | 4 | -2.593377175 |
| GO Biological Processes | GO:0016079 | synaptic vesicle exocytosis | 4 | -2.593377175 |
| GO Biological Processes | GO:0030808 | regulation of nucleotide biosynthetic process | 4 | -2.593377175 |
| GO Biological Processes | GO:0051153 | regulation of striated muscle cell differentiation | 4 | -2.593377175 |
| GO Biological Processes | GO:0002090 | regulation of receptor internalization | 3 | -2.591613046 |
| GO Biological Processes | GO:0009260 | ribonucleotide biosynthetic process | 6 | -2.574404312 |
| GO Biological Processes | GO:0001658 | branching involved in ureteric bud morphogenesis | 3 | -2.570424137 |
| GO Biological Processes | GO:0017001 | antibiotic catabolic process | 3 | -2.570424137 |
| GO Biological Processes | GO:0031102 | neuron projection regeneration | 3 | -2.570424137 |
| GO Biological Processes | GO:0060348 | bone development | 5 | -2.556966325 |
| GO Biological Processes | GO:0032515 | negative regulation of phosphoprotein phosphatase activity | 3 | -2.5496225 |
| GO Biological Processes | GO:0048747 | muscle fiber development | 3 | -2.5496225 |
| GO Biological Processes | GO:0098930 | axonal transport | 3 | -2.5496225 |
| GO Biological Processes | GO:0003014 | renal system process | 4 | -2.542395479 |
| GO Biological Processes | GO:0035270 | endocrine system development | 4 | -2.542395479 |
| GO Biological Processes | GO:0050866 | negative regulation of cell activation | 5 | -2.529763802 |
| GO Biological Processes | GO:0061013 | regulation of mRNA catabolic process | 5 | -2.520800012 |
| GO Biological Processes | GO:0046390 | ribose phosphate biosynthetic process | 6 | -2.516946406 |
| GO Biological Processes | GO:0010975 | regulation of neuron projection development | 8 | -2.513827154 |
| GO Biological Processes | GO:0009124 | nucleoside monophosphate biosynthetic process | 5 | -2.511887241 |
| GO Biological Processes | GO:0007405 | neuroblast proliferation | 3 | -2.509129545 |
| GO Biological Processes | GO:0010518 | positive regulation of phospholipase activity | 3 | -2.509129545 |
| GO Biological Processes | GO:0010676 | positive regulation of cellular carbohydrate metabolic process | 3 | -2.509129545 |
| GO Biological Processes | GO:0010823 | negative regulation of mitochondrion organization | 3 | -2.509129545 |
| GO Biological Processes | GO:0072089 | stem cell proliferation | 4 | -2.505360074 |
| GO Biological Processes | GO:0006732 | coenzyme metabolic process | 7 | -2.50072739 |
| GO Biological Processes | GO:0016055 | Wnt signaling pathway | 8 | -2.498615092 |
| GO Biological Processes | GO:0006164 | purine nucleotide biosynthetic process | 6 | -2.49588256 |
| GO Biological Processes | GO:0031396 | regulation of protein ubiquitination | 5 | -2.494212785 |
| GO Biological Processes | GO:0046717 | acid secretion | 4 | -2.493234002 |
| GO Biological Processes | GO:0006511 | ubiquitin-dependent protein catabolic process | 9 | -2.490799063 |
| GO Biological Processes | GO:0034113 | heterotypic cell-cell adhesion | 3 | -2.48941405 |
| GO Biological Processes | GO:0198738 | cell-cell signaling by wnt | 8 | -2.488538578 |
| GO Biological Processes | GO:0002437 | inflammatory response to antigenic stimulus | 3 | -2.470037397 |
| GO Biological Processes | GO:0042130 | negative regulation of T cell proliferation | 3 | -2.470037397 |
| GO Biological Processes | GO:1900449 | regulation of glutamate receptor signaling pathway | 3 | -2.470037397 |
| GO Biological Processes | GO:2000401 | regulation of lymphocyte migration | 3 | -2.470037397 |
| GO Biological Processes | GO:0019941 | modification-dependent protein catabolic process | 9 | -2.464223598 |
| GO Biological Processes | GO:0032434 | regulation of proteasomal ubiquitin-dependent protein catabolic process | 4 | -2.457489205 |
| GO Biological Processes | GO:0044106 | cellular amine metabolic process | 4 | -2.457489205 |
| GO Biological Processes | GO:0010830 | regulation of myotube differentiation | 3 | -2.450988897 |
| GO Biological Processes | GO:0035308 | negative regulation of protein dephosphorylation | 3 | -2.450988897 |
| GO Biological Processes | GO:0048247 | lymphocyte chemotaxis | 3 | -2.450988897 |
| GO Biological Processes | GO:0060675 | ureteric bud morphogenesis | 3 | -2.450988897 |
| GO Biological Processes | GO:0006165 | nucleoside diphosphate phosphorylation | 4 | -2.445780012 |
| GO Biological Processes | GO:1901137 | carbohydrate derivative biosynthetic process | 10 | -2.435615466 |
| GO Biological Processes | GO:0045995 | regulation of embryonic development | 4 | -2.434171081 |
| GO Biological Processes | GO:0097164 | ammonium ion metabolic process | 5 | -2.433888958 |
| GO Biological Processes | GO:0034394 | protein localization to cell surface | 3 | -2.432258359 |
| GO Biological Processes | GO:0050922 | negative regulation of chemotaxis | 3 | -2.432258359 |
| GO Biological Processes | GO:0072171 | mesonephric tubule morphogenesis | 3 | -2.432258359 |
| GO Biological Processes | GO:0046939 | nucleotide phosphorylation | 4 | -2.422660901 |
| GO Biological Processes | GO:0050853 | B cell receptor signaling pathway | 4 | -2.422660901 |
| GO Biological Processes | GO:0071333 | cellular response to glucose stimulus | 4 | -2.422660901 |
| GO Biological Processes | GO:0072522 | purine-containing compound biosynthetic process | 6 | -2.420794548 |
| GO Biological Processes | GO:0043632 | modification-dependent macromolecule catabolic process | 9 | -2.416379147 |
| GO Biological Processes | GO:0071300 | cellular response to retinoic acid | 3 | -2.413836059 |
| GO Biological Processes | GO:1903672 | positive regulation of sprouting angiogenesis | 3 | -2.413836059 |
| GO Biological Processes | GO:1905207 | regulation of cardiocyte differentiation | 3 | -2.413836059 |
| GO Biological Processes | GO:0006997 | nucleus organization | 4 | -2.411247993 |
| GO Biological Processes | GO:0045727 | positive regulation of translation | 4 | -2.411247993 |
| GO Biological Processes | GO:0043588 | skin development | 7 | -2.403913202 |
| GO Biological Processes | GO:0042552 | myelination | 4 | -2.399930913 |
| GO Biological Processes | GO:0060048 | cardiac muscle contraction | 4 | -2.399930913 |
| GO Biological Processes | GO:0071331 | cellular response to hexose stimulus | 4 | -2.399930913 |
| GO Biological Processes | GO:0002548 | monocyte chemotaxis | 3 | -2.395712711 |
| GO Biological Processes | GO:0016239 | positive regulation of macroautophagy | 3 | -2.395712711 |
| GO Biological Processes | GO:0051656 | establishment of organelle localization | 8 | -2.395321284 |
| GO Biological Processes | GO:0019730 | antimicrobial humoral response | 4 | -2.388708248 |
| GO Biological Processes | GO:0046330 | positive regulation of JNK cascade | 4 | -2.388708248 |
| GO Biological Processes | GO:0071326 | cellular response to monosaccharide stimulus | 4 | -2.388708248 |
| GO Biological Processes | GO:0072329 | monocarboxylic acid catabolic process | 4 | -2.388708248 |
| GO Biological Processes | GO:0002753 | cytoplasmic pattern recognition receptor signaling pathway | 3 | -2.377879445 |
| GO Biological Processes | GO:0044257 | cellular protein catabolic process | 10 | -2.370033496 |
| GO Biological Processes | GO:1903050 | regulation of proteolysis involved in cellular protein catabolic process | 5 | -2.367723677 |
| GO Biological Processes | GO:0050715 | positive regulation of cytokine secretion | 3 | -2.360327776 |
| GO Biological Processes | GO:0007292 | female gamete generation | 4 | -2.344734537 |
| GO Biological Processes | GO:0034404 | nucleobase-containing small molecule biosynthetic process | 5 | -2.343634147 |
| GO Biological Processes | GO:0008088 | axo-dendritic transport | 3 | -2.343049587 |
| GO Biological Processes | GO:0050795 | regulation of behavior | 3 | -2.343049587 |
| GO Biological Processes | GO:0051937 | catecholamine transport | 3 | -2.326037108 |
| GO Biological Processes | GO:0070988 | demethylation | 3 | -2.326037108 |
| GO Biological Processes | GO:0072006 | nephron development | 4 | -2.323279622 |
| GO Biological Processes | GO:0019882 | antigen processing and presentation | 5 | -2.319922484 |
| GO Biological Processes | GO:0002700 | regulation of production of molecular mediator of immune response | 4 | -2.312680784 |
| GO Biological Processes | GO:0072078 | nephron tubule morphogenesis | 3 | -2.309282893 |
| GO Biological Processes | GO:0006305 | DNA alkylation | 3 | -2.292779805 |
| GO Biological Processes | GO:0006306 | DNA methylation | 3 | -2.292779805 |
| GO Biological Processes | GO:0010517 | regulation of phospholipase activity | 3 | -2.292779805 |
| GO Biological Processes | GO:0032024 | positive regulation of insulin secretion | 3 | -2.292779805 |
| GO Biological Processes | GO:0032481 | positive regulation of type I interferon production | 3 | -2.292779805 |
| GO Biological Processes | GO:0002831 | regulation of response to biotic stimulus | 4 | -2.291734403 |
| GO Biological Processes | GO:0048588 | developmental cell growth | 5 | -2.281216201 |
| GO Biological Processes | GO:0002479 | antigen processing and presentation of exogenous peptide antigen via MHC class I, TAP-dependent | 3 | -2.276520999 |
| GO Biological Processes | GO:0015909 | long-chain fatty acid transport | 3 | -2.276520999 |
| GO Biological Processes | GO:0061844 | antimicrobial humoral immune response mediated by antimicrobial peptide | 3 | -2.276520999 |
| GO Biological Processes | GO:0090263 | positive regulation of canonical Wnt signaling pathway | 4 | -2.260925956 |
| GO Biological Processes | GO:1903670 | regulation of sprouting angiogenesis | 4 | -2.260925956 |
| GO Biological Processes | GO:0060193 | positive regulation of lipase activity | 3 | -2.260499906 |
| GO Biological Processes | GO:0006081 | cellular aldehyde metabolic process | 3 | -2.244710217 |
| GO Biological Processes | GO:0019674 | NAD metabolic process | 3 | -2.244710217 |
| GO Biological Processes | GO:0030500 | regulation of bone mineralization | 3 | -2.244710217 |
| GO Biological Processes | GO:0061333 | renal tubule morphogenesis | 3 | -2.244710217 |
| GO Biological Processes | GO:0070830 | bicellular tight junction assembly | 3 | -2.244710217 |
| GO Biological Processes | GO:1903311 | regulation of mRNA metabolic process | 6 | -2.243589937 |
| GO Biological Processes | GO:0007605 | sensory perception of sound | 4 | -2.240781636 |
| GO Biological Processes | GO:0030433 | ubiquitin-dependent ERAD pathway | 3 | -2.229145872 |
| GO Biological Processes | GO:0032413 | negative regulation of ion transmembrane transporter activity | 3 | -2.229145872 |
| GO Biological Processes | GO:0033143 | regulation of intracellular steroid hormone receptor signaling pathway | 3 | -2.229145872 |
| GO Biological Processes | GO:0050688 | regulation of defense response to virus | 3 | -2.229145872 |
| GO Biological Processes | GO:0002250 | adaptive immune response | 9 | -2.227841631 |
| GO Biological Processes | GO:0017156 | calcium ion regulated exocytosis | 4 | -2.220943268 |
| GO Biological Processes | GO:0000422 | autophagy of mitochondrion | 3 | -2.213801043 |
| GO Biological Processes | GO:0006446 | regulation of translational initiation | 3 | -2.213801043 |
| GO Biological Processes | GO:0031016 | pancreas development | 3 | -2.213801043 |
| GO Biological Processes | GO:0043407 | negative regulation of MAP kinase activity | 3 | -2.213801043 |
| GO Biological Processes | GO:0050672 | negative regulation of lymphocyte proliferation | 3 | -2.213801043 |
| GO Biological Processes | GO:0060395 | SMAD protein signal transduction | 3 | -2.213801043 |
| GO Biological Processes | GO:0061726 | mitochondrion disassembly | 3 | -2.213801043 |
| GO Biological Processes | GO:0099601 | regulation of neurotransmitter receptor activity | 3 | -2.213801043 |
| GO Biological Processes | GO:1900034 | regulation of cellular response to heat | 3 | -2.213801043 |
| GO Biological Processes | GO:0048592 | eye morphogenesis | 4 | -2.201402854 |
| GO Biological Processes | GO:0006110 | regulation of glycolytic process | 3 | -2.198670128 |
| GO Biological Processes | GO:0016575 | histone deacetylation | 3 | -2.198670128 |
| GO Biological Processes | GO:0031397 | negative regulation of protein ubiquitination | 3 | -2.198670128 |
| GO Biological Processes | GO:0032204 | regulation of telomere maintenance | 3 | -2.198670128 |
| GO Biological Processes | GO:0032945 | negative regulation of mononuclear cell proliferation | 3 | -2.198670128 |
| GO Biological Processes | GO:0042590 | antigen processing and presentation of exogenous peptide antigen via MHC class I | 3 | -2.198670128 |
| GO Biological Processes | GO:0120192 | tight junction assembly | 3 | -2.198670128 |
| GO Biological Processes | GO:0048489 | synaptic vesicle transport | 4 | -2.191741963 |
| GO Biological Processes | GO:0097480 | establishment of synaptic vesicle localization | 4 | -2.191741963 |
| GO Biological Processes | GO:1903900 | regulation of viral life cycle | 4 | -2.191741963 |
| GO Biological Processes | GO:0001960 | negative regulation of cytokine-mediated signaling pathway | 3 | -2.183747735 |
| GO Biological Processes | GO:0003151 | outflow tract morphogenesis | 3 | -2.183747735 |
| GO Biological Processes | GO:0033238 | regulation of cellular amine metabolic process | 3 | -2.183747735 |
| GO Biological Processes | GO:0030203 | glycosaminoglycan metabolic process | 4 | -2.1821527 |
| GO Biological Processes | GO:1903707 | negative regulation of hemopoiesis | 4 | -2.1821527 |
| GO Biological Processes | GO:2000241 | regulation of reproductive process | 4 | -2.1821527 |
| GO Biological Processes | GO:0032675 | regulation of interleukin-6 production | 4 | -2.172634149 |
| GO Biological Processes | GO:0002697 | regulation of immune effector process | 7 | -2.170367509 |
| GO Biological Processes | GO:0000086 | G2/M transition of mitotic cell cycle | 5 | -2.156701533 |
| GO Biological Processes | GO:0001570 | vasculogenesis | 3 | -2.154507941 |
| GO Biological Processes | GO:0002718 | regulation of cytokine production involved in immune response | 3 | -2.154507941 |
| GO Biological Processes | GO:0031145 | anaphase-promoting complex-dependent catabolic process | 3 | -2.154507941 |
| GO Biological Processes | GO:0120193 | tight junction organization | 3 | -2.154507941 |
| GO Biological Processes | GO:0030279 | negative regulation of ossification | 3 | -2.140180723 |
| GO Biological Processes | GO:0034502 | protein localization to chromosome | 3 | -2.140180723 |
| GO Biological Processes | GO:0008016 | regulation of heart contraction | 5 | -2.135789353 |
| GO Biological Processes | GO:0001764 | neuron migration | 4 | -2.126071204 |
| GO Biological Processes | GO:0014031 | mesenchymal cell development | 3 | -2.126042374 |
| GO Biological Processes | GO:0032092 | positive regulation of protein binding | 3 | -2.126042374 |
| GO Biological Processes | GO:0043297 | apical junction assembly | 3 | -2.126042374 |
| GO Biological Processes | GO:1903362 | regulation of cellular protein catabolic process | 5 | -2.115176248 |
| GO Biological Processes | GO:0007409 | axonogenesis | 7 | -2.113067001 |
| GO Biological Processes | GO:2000106 | regulation of leukocyte apoptotic process | 3 | -2.112088414 |
| GO Biological Processes | GO:0030512 | negative regulation of transforming growth factor beta receptor signaling pathway | 3 | -2.098314521 |
| GO Biological Processes | GO:0060291 | long-term synaptic potentiation | 3 | -2.098314521 |
| GO Biological Processes | GO:0060761 | negative regulation of response to cytokine stimulus | 3 | -2.098314521 |
| GO Biological Processes | GO:0090049 | regulation of cell migration involved in sprouting angiogenesis | 3 | -2.098314521 |
| GO Biological Processes | GO:0002819 | regulation of adaptive immune response | 4 | -2.090006374 |
| GO Biological Processes | GO:0097479 | synaptic vesicle localization | 4 | -2.090006374 |
| GO Biological Processes | GO:0022412 | cellular process involved in reproduction in multicellular organism | 6 | -2.089298946 |
| GO Biological Processes | GO:0061448 | connective tissue development | 5 | -2.088145345 |
| GO Biological Processes | GO:0002460 | adaptive immune response based on somatic recombination of immune receptors built from immunoglobulin superfamily domains | 6 | -2.083864713 |
| GO Biological Processes | GO:0032635 | interleukin-6 production | 4 | -2.072351701 |
| GO Biological Processes | GO:0032410 | negative regulation of transporter activity | 3 | -2.071290387 |
| GO Biological Processes | GO:0043506 | regulation of JUN kinase activity | 3 | -2.071290387 |
| GO Biological Processes | GO:1903845 | negative regulation of cellular response to transforming growth factor beta stimulus | 3 | -2.071290387 |
| GO Biological Processes | GO:0030111 | regulation of Wnt signaling pathway | 6 | -2.062316326 |
| GO Biological Processes | GO:0006022 | aminoglycan metabolic process | 4 | -2.054940892 |
| GO Biological Processes | GO:0032680 | regulation of tumor necrosis factor production | 4 | -2.046325144 |
| GO Biological Processes | GO:0050954 | sensory perception of mechanical stimulus | 4 | -2.046325144 |
| GO Biological Processes | GO:0097306 | cellular response to alcohol | 3 | -2.044938271 |
| GO Biological Processes | GO:0022604 | regulation of cell morphogenesis | 7 | -2.035111803 |
| GO Biological Processes | GO:0032760 | positive regulation of tumor necrosis factor production | 3 | -2.03200489 |
| GO Biological Processes | GO:1904063 | negative regulation of cation transmembrane transport | 3 | -2.03200489 |
| GO Biological Processes | GO:0007269 | neurotransmitter secretion | 4 | -2.029269482 |
| GO Biological Processes | GO:0099643 | signal release from synapse | 4 | -2.029269482 |
| GO Biological Processes | GO:0032535 | regulation of cellular component size | 6 | -2.025317052 |
| GO Biological Processes | GO:1903555 | regulation of tumor necrosis factor superfamily cytokine production | 4 | -2.020828214 |
| GO Biological Processes | GO:0061097 | regulation of protein tyrosine kinase activity | 3 | -2.019228564 |
| GO Biological Processes | GO:0002478 | antigen processing and presentation of exogenous peptide antigen | 4 | -2.012443771 |
| GO Biological Processes | GO:0030641 | regulation of cellular pH | 3 | -2.006605887 |
| GO Biological Processes | GO:0060349 | bone morphogenesis | 3 | -2.006605887 |
| GO Biological Processes | GO:1903557 | positive regulation of tumor necrosis factor superfamily cytokine production | 3 | -2.006605887 |
| GO Biological Processes | GO:0032640 | tumor necrosis factor production | 4 | -2.004115504 |
| GO Cellular Components | GO:0045121 | membrane raft | 18 | -13.64221261 |
| GO Cellular Components | GO:0098857 | membrane microdomain | 18 | -13.61968108 |
| GO Cellular Components | GO:0098589 | membrane region | 18 | -13.33341382 |
| GO Cellular Components | GO:0005925 | focal adhesion | 11 | -5.336738816 |
| GO Cellular Components | GO:0005924 | cell-substrate adherens junction | 11 | -5.307295508 |
| GO Cellular Components | GO:0030055 | cell-substrate junction | 11 | -5.258773686 |
| GO Cellular Components | GO:0005912 | adherens junction | 12 | -4.919935271 |
| GO Cellular Components | GO:0070161 | anchoring junction | 12 | -4.79749438 |
| GO Cellular Components | GO:0044853 | plasma membrane raft | 6 | -4.782363269 |
| GO Cellular Components | GO:0005901 | caveola | 5 | -4.34636858 |
| GO Cellular Components | GO:1902911 | protein kinase complex | 9 | -8.81279998 |
| GO Cellular Components | GO:1902554 | serine/threonine protein kinase complex | 8 | -8.02120529 |
| GO Cellular Components | GO:0000307 | cyclin-dependent protein kinase holoenzyme complex | 6 | -7.30546851 |
| GO Cellular Components | GO:0061695 | transferase complex, transferring phosphorus-containing groups | 10 | -6.472506001 |
| GO Cellular Components | GO:1990234 | transferase complex | 11 | -2.989136923 |
| GO Cellular Components | GO:0005815 | microtubule organizing center | 11 | -2.930929602 |
| GO Cellular Components | GO:0005819 | spindle | 7 | -2.757115466 |
| GO Cellular Components | GO:0005813 | centrosome | 8 | -2.204169857 |
| GO Cellular Components | GO:0031983 | vesicle lumen | 12 | -7.130909309 |
| GO Cellular Components | GO:0034774 | secretory granule lumen | 11 | -6.436304986 |
| GO Cellular Components | GO:0060205 | cytoplasmic vesicle lumen | 11 | -6.215824024 |
| GO Cellular Components | GO:0031093 | platelet alpha granule lumen | 4 | -3.553761472 |
| GO Cellular Components | GO:0031091 | platelet alpha granule | 4 | -3.048859997 |
| GO Cellular Components | GO:0031012 | extracellular matrix | 15 | -7.119008417 |
| GO Cellular Components | GO:0062023 | collagen-containing extracellular matrix | 12 | -6.064349822 |
| GO Cellular Components | GO:0031968 | organelle outer membrane | 10 | -7.072063819 |
| GO Cellular Components | GO:0019867 | outer membrane | 10 | -7.034981781 |
| GO Cellular Components | GO:0005741 | mitochondrial outer membrane | 9 | -6.494492312 |
| GO Cellular Components | GO:0005740 | mitochondrial envelope | 15 | -5.470526475 |
| GO Cellular Components | GO:0031966 | mitochondrial membrane | 13 | -4.415632217 |
| GO Cellular Components | GO:0005667 | transcription factor complex | 12 | -7.048521724 |
| GO Cellular Components | GO:0090575 | RNA polymerase II transcription factor complex | 7 | -5.003496993 |
| GO Cellular Components | GO:0044798 | nuclear transcription factor complex | 7 | -4.731645742 |
| GO Cellular Components | GO:0098797 | plasma membrane protein complex | 15 | -6.024115195 |
| GO Cellular Components | GO:0043235 | receptor complex | 10 | -3.772587225 |
| GO Cellular Components | GO:0098802 | plasma membrane receptor complex | 6 | -2.481982111 |
| GO Cellular Components | GO:0045177 | apical part of cell | 12 | -5.990697146 |
| GO Cellular Components | GO:0016324 | apical plasma membrane | 9 | -4.256841536 |
| GO Cellular Components | GO:0005769 | early endosome | 8 | -3.331876 |
| GO Cellular Components | GO:1904813 | ficolin-1-rich granule lumen | 7 | -5.69938427 |
| GO Cellular Components | GO:0101002 | ficolin-1-rich granule | 7 | -4.560540468 |
| GO Cellular Components | GO:1904724 | tertiary granule lumen | 3 | -2.657644476 |
| GO Cellular Components | GO:0070820 | tertiary granule | 4 | -2.126071204 |
| GO Cellular Components | GO:0044297 | cell body | 13 | -5.354780022 |
| GO Cellular Components | GO:0043025 | neuronal cell body | 12 | -5.178399652 |
| GO Cellular Components | GO:0030425 | dendrite | 13 | -4.971014074 |
| GO Cellular Components | GO:0097447 | dendritic tree | 13 | -4.956742618 |
| GO Cellular Components | GO:0098552 | side of membrane | 13 | -5.102006175 |
| GO Cellular Components | GO:0009897 | external side of plasma membrane | 10 | -4.551491986 |
| GO Cellular Components | GO:0030424 | axon | 13 | -4.963871406 |
| GO Cellular Components | GO:0098978 | glutamatergic synapse | 8 | -3.509761221 |
| GO Cellular Components | GO:0099055 | integral component of postsynaptic membrane | 4 | -2.646303344 |
| GO Cellular Components | GO:0098936 | intrinsic component of postsynaptic membrane | 4 | -2.580454522 |
| GO Cellular Components | GO:0033267 | axon part | 7 | -2.512459306 |
| GO Cellular Components | GO:0098794 | postsynapse | 9 | -2.486345968 |
| GO Cellular Components | GO:0043679 | axon terminus | 4 | -2.481214599 |
| GO Cellular Components | GO:0098793 | presynapse | 8 | -2.473520095 |
| GO Cellular Components | GO:0099056 | integral component of presynaptic membrane | 3 | -2.292779805 |
| GO Cellular Components | GO:0044306 | neuron projection terminus | 4 | -2.271115437 |
| GO Cellular Components | GO:0099699 | integral component of synaptic membrane | 4 | -2.220943268 |
| GO Cellular Components | GO:0042734 | presynaptic membrane | 4 | -2.201402854 |
| GO Cellular Components | GO:0098889 | intrinsic component of presynaptic membrane | 3 | -2.154507941 |
| GO Cellular Components | GO:0099240 | intrinsic component of synaptic membrane | 4 | -2.107910829 |
| GO Cellular Components | GO:0005635 | nuclear envelope | 11 | -4.833583349 |
| GO Cellular Components | GO:0031965 | nuclear membrane | 7 | -3.255415031 |
| GO Cellular Components | GO:0000323 | lytic vacuole | 13 | -4.496075012 |
| GO Cellular Components | GO:0005764 | lysosome | 13 | -4.496075012 |
| GO Cellular Components | GO:0030139 | endocytic vesicle | 6 | -2.440946616 |
| GO Cellular Components | GO:0048471 | perinuclear region of cytoplasm | 13 | -4.40952123 |
| GO Cellular Components | GO:0043209 | myelin sheath | 4 | -4.155575546 |
| GO Cellular Components | GO:0005902 | microvillus | 4 | -3.013585002 |
| GO Cellular Components | GO:0044445 | cytosolic part | 7 | -3.751569189 |
| GO Cellular Components | GO:0005788 | endoplasmic reticulum lumen | 7 | -3.196501766 |
| GO Cellular Components | GO:0120111 | neuron projection cytoplasm | 4 | -3.122051908 |
| GO Cellular Components | GO:1904115 | axon cytoplasm | 3 | -2.613202957 |
| GO Cellular Components | GO:0099568 | cytoplasmic region | 8 | -2.570624795 |
| GO Cellular Components | GO:0032838 | plasma membrane bounded cell projection cytoplasm | 5 | -2.485450134 |
| GO Cellular Components | GO:0030665 | clathrin-coated vesicle membrane | 4 | -2.632882235 |
| GO Cellular Components | GO:0005911 | cell-cell junction | 7 | -2.554112794 |
| GO Cellular Components | GO:0032993 | protein-DNA complex | 5 | -2.547846446 |
| GO Cellular Components | GO:0098685 | Schaffer collateral - CA1 synapse | 3 | -2.140180723 |
| GO Cellular Components | GO:1905368 | peptidase complex | 3 | -2.098314521 |
| GO Cellular Components | GO:0031970 | organelle envelope lumen | 3 | -2.058032224 |
| GO Molecular Functions | GO:0008134 | transcription factor binding | 31 | -22.02467927 |
| GO Molecular Functions | GO:0001085 | RNA polymerase II transcription factor binding | 14 | -14.11370595 |
| GO Molecular Functions | GO:0001228 | DNA-binding transcription activator activity, RNA polymerase II-specific | 17 | -9.467202822 |
| GO Molecular Functions | GO:0003682 | chromatin binding | 17 | -8.522831858 |
| GO Molecular Functions | GO:0004879 | nuclear receptor activity | 11 | -15.10336693 |
| GO Molecular Functions | GO:0098531 | transcription factor activity, direct ligand regulated sequence-specific DNA binding | 11 | -15.10336693 |
| GO Molecular Functions | GO:0003707 | steroid hormone receptor activity | 7 | -10.59297961 |
| GO Molecular Functions | GO:0001223 | transcription coactivator binding | 7 | -10.22278953 |
| GO Molecular Functions | GO:0001221 | transcription cofactor binding | 8 | -10.00165981 |
| GO Molecular Functions | GO:0008289 | lipid binding | 20 | -9.092708527 |
| GO Molecular Functions | GO:0005496 | steroid binding | 8 | -7.517783985 |
| GO Molecular Functions | GO:0001091 | RNA polymerase II basal transcription factor binding | 4 | -5.774437768 |
| GO Molecular Functions | GO:0001225 | RNA polymerase II transcription coactivator binding | 3 | -5.702789222 |
| GO Molecular Functions | GO:0001224 | RNA polymerase II transcription cofactor binding | 3 | -5.258613184 |
| GO Molecular Functions | GO:0001098 | basal transcription machinery binding | 4 | -3.433766973 |
| GO Molecular Functions | GO:0001099 | basal RNA polymerase II transcription machinery binding | 4 | -3.433766973 |
| GO Molecular Functions | GO:0008013 | beta-catenin binding | 4 | -3.14093035 |
| GO Molecular Functions | GO:0019904 | protein domain specific binding | 25 | -14.43941013 |
| GO Molecular Functions | GO:0044389 | ubiquitin-like protein ligase binding | 14 | -9.514038204 |
| GO Molecular Functions | GO:0031625 | ubiquitin protein ligase binding | 12 | -7.774466157 |
| GO Molecular Functions | GO:0019901 | protein kinase binding | 23 | -13.12940748 |
| GO Molecular Functions | GO:0019900 | kinase binding | 24 | -12.96095126 |
| GO Molecular Functions | GO:0004672 | protein kinase activity | 20 | -11.4861577 |
| GO Molecular Functions | GO:0016773 | phosphotransferase activity, alcohol group as acceptor | 21 | -11.06896286 |
| GO Molecular Functions | GO:0016301 | kinase activity | 21 | -10.32540368 |
| GO Molecular Functions | GO:0004674 | protein serine/threonine kinase activity | 14 | -7.713969301 |
| GO Molecular Functions | GO:0042803 | protein homodimerization activity | 22 | -12.22939077 |
| GO Molecular Functions | GO:0005126 | cytokine receptor binding | 14 | -10.39278156 |
| GO Molecular Functions | GO:0005125 | cytokine activity | 11 | -7.820230272 |
| GO Molecular Functions | GO:0030545 | receptor regulator activity | 15 | -7.478209366 |
| GO Molecular Functions | GO:0048018 | receptor ligand activity | 14 | -7.131273848 |
| GO Molecular Functions | GO:0042379 | chemokine receptor binding | 5 | -4.705289241 |
| GO Molecular Functions | GO:0045236 | CXCR chemokine receptor binding | 3 | -4.110002877 |
| GO Molecular Functions | GO:0008009 | chemokine activity | 4 | -4.083944805 |
| GO Molecular Functions | GO:1901681 | sulfur compound binding | 7 | -3.596692864 |
| GO Molecular Functions | GO:0001664 | G protein-coupled receptor binding | 7 | -3.324799294 |
| GO Molecular Functions | GO:0008201 | heparin binding | 4 | -2.08114819 |
| GO Molecular Functions | GO:0016209 | antioxidant activity | 9 | -9.558401762 |
| GO Molecular Functions | GO:0016491 | oxidoreductase activity | 20 | -9.393763175 |
| GO Molecular Functions | GO:0020037 | heme binding | 10 | -8.975050604 |
| GO Molecular Functions | GO:0046906 | tetrapyrrole binding | 10 | -8.678310198 |
| GO Molecular Functions | GO:0004601 | peroxidase activity | 7 | -8.328485354 |
| GO Molecular Functions | GO:0016684 | oxidoreductase activity, acting on peroxide as acceptor | 7 | -8.096295993 |
| GO Molecular Functions | GO:0048037 | cofactor binding | 15 | -7.752202094 |
| GO Molecular Functions | GO:0016705 | oxidoreductase activity, acting on paired donors, with incorporation or reduction of molecular oxygen | 7 | -4.915354209 |
| GO Molecular Functions | GO:0051213 | dioxygenase activity | 3 | -2.044938271 |
| GO Molecular Functions | GO:0002020 | protease binding | 10 | -9.006009565 |
| GO Molecular Functions | GO:0070491 | repressing transcription factor binding | 8 | -8.66654378 |
| GO Molecular Functions | GO:0051059 | NF-kappaB binding | 4 | -4.883897344 |
| GO Molecular Functions | GO:0001103 | RNA polymerase II repressing transcription factor binding | 3 | -3.231029424 |
| GO Molecular Functions | GO:0001227 | DNA-binding transcription repressor activity, RNA polymerase II-specific | 7 | -2.983618669 |
| GO Molecular Functions | GO:0004252 | serine-type endopeptidase activity | 10 | -8.07179363 |
| GO Molecular Functions | GO:0008236 | serine-type peptidase activity | 10 | -7.65663042 |
| GO Molecular Functions | GO:0004175 | endopeptidase activity | 14 | -7.627850948 |
| GO Molecular Functions | GO:0017171 | serine hydrolase activity | 10 | -7.570297735 |
| GO Molecular Functions | GO:0070011 | peptidase activity, acting on L-amino acid peptides | 16 | -7.468075905 |
| GO Molecular Functions | GO:0008233 | peptidase activity | 16 | -7.296462928 |
| GO Molecular Functions | GO:0008237 | metallopeptidase activity | 6 | -3.565692575 |
| GO Molecular Functions | GO:0004222 | metalloendopeptidase activity | 4 | -2.773232951 |
| GO Molecular Functions | GO:0035173 | histone kinase activity | 5 | -7.910380643 |
| GO Molecular Functions | GO:0030332 | cyclin binding | 3 | -3.430996962 |
| GO Molecular Functions | GO:0035257 | nuclear hormone receptor binding | 9 | -7.56523129 |
| GO Molecular Functions | GO:0051427 | hormone receptor binding | 9 | -6.79399941 |
| GO Molecular Functions | GO:0035258 | steroid hormone receptor binding | 5 | -4.397430772 |
| GO Molecular Functions | GO:0030331 | estrogen receptor binding | 4 | -4.350081673 |
| GO Molecular Functions | GO:0016922 | nuclear receptor binding | 3 | -3.967762904 |
| GO Molecular Functions | GO:0030374 | nuclear receptor transcription coactivator activity | 3 | -2.68052748 |
| GO Molecular Functions | GO:0005178 | integrin binding | 9 | -7.56523129 |
| GO Molecular Functions | GO:0050839 | cell adhesion molecule binding | 13 | -6.008881723 |
| GO Molecular Functions | GO:0019902 | phosphatase binding | 10 | -7.549023625 |
| GO Molecular Functions | GO:0019903 | protein phosphatase binding | 8 | -6.274997095 |
| GO Molecular Functions | GO:0051721 | protein phosphatase 2A binding | 4 | -4.827479404 |
| GO Molecular Functions | GO:0101020 | estrogen 16-alpha-hydroxylase activity | 4 | -7.500370417 |
| GO Molecular Functions | GO:0101021 | estrogen 2-hydroxylase activity | 3 | -6.00232777 |
| GO Molecular Functions | GO:0070330 | aromatase activity | 4 | -5.270186338 |
| GO Molecular Functions | GO:0016712 | oxidoreductase activity, acting on paired donors, with incorporation or reduction of molecular oxygen, reduced flavin or flavoprotein as one donor, and incorporation of one atom of oxygen | 4 | -4.669020277 |
| GO Molecular Functions | GO:0008395 | steroid hydroxylase activity | 4 | -4.524594321 |
| GO Molecular Functions | GO:0019825 | oxygen binding | 3 | -3.19472315 |
| GO Molecular Functions | GO:0005506 | iron ion binding | 5 | -3.132601287 |
| GO Molecular Functions | GO:0004497 | monooxygenase activity | 4 | -2.864626325 |
| GO Molecular Functions | GO:0046982 | protein heterodimerization activity | 12 | -7.403172311 |
| GO Molecular Functions | GO:0051434 | BH3 domain binding | 3 | -5.702789222 |
| GO Molecular Functions | GO:0051400 | BH domain binding | 3 | -4.793787993 |
| GO Molecular Functions | GO:0070513 | death domain binding | 3 | -4.793787993 |
| GO Molecular Functions | GO:0001046 | core promoter sequence-specific DNA binding | 6 | -7.066289779 |
| GO Molecular Functions | GO:0001047 | core promoter binding | 6 | -7.066289779 |
| GO Molecular Functions | GO:0000979 | RNA polymerase II core promoter sequence-specific DNA binding | 3 | -3.724403023 |
| GO Molecular Functions | GO:0033613 | activating transcription factor binding | 7 | -7.000060108 |
| GO Molecular Functions | GO:0001102 | RNA polymerase II activating transcription factor binding | 4 | -4.155575546 |
| GO Molecular Functions | GO:0070412 | R-SMAD binding | 3 | -3.780908985 |
| GO Molecular Functions | GO:0046332 | SMAD binding | 4 | -3.280234157 |
| GO Molecular Functions | GO:0031072 | heat shock protein binding | 8 | -6.807426854 |
| GO Molecular Functions | GO:0051879 | Hsp90 protein binding | 5 | -5.812901815 |
| GO Molecular Functions | GO:0030544 | Hsp70 protein binding | 3 | -2.997366675 |
| GO Molecular Functions | GO:0019207 | kinase regulator activity | 9 | -6.065705598 |
| GO Molecular Functions | GO:0019887 | protein kinase regulator activity | 8 | -5.567739615 |
| GO Molecular Functions | GO:0008047 | enzyme activator activity | 12 | -5.10378619 |
| GO Molecular Functions | GO:0016538 | cyclin-dependent protein serine/threonine kinase regulator activity | 4 | -4.049303022 |
| GO Molecular Functions | GO:0030291 | protein serine/threonine kinase inhibitor activity | 3 | -3.388277133 |
| GO Molecular Functions | GO:0030295 | protein kinase activator activity | 4 | -3.21893617 |
| GO Molecular Functions | GO:0019209 | kinase activator activity | 4 | -3.085002242 |
| GO Molecular Functions | GO:0004860 | protein kinase inhibitor activity | 3 | -2.450988897 |
| GO Molecular Functions | GO:0019210 | kinase inhibitor activity | 3 | -2.377879445 |
| GO Molecular Functions | GO:0004857 | enzyme inhibitor activity | 6 | -2.020103681 |
| GO Molecular Functions | GO:0033218 | amide binding | 11 | -5.699898051 |
| GO Molecular Functions | GO:0042277 | peptide binding | 9 | -4.703843736 |
| GO Molecular Functions | GO:0043295 | glutathione binding | 3 | -4.670338895 |
| GO Molecular Functions | GO:1900750 | oligopeptide binding | 3 | -4.557884878 |
| GO Molecular Functions | GO:0005504 | fatty acid binding | 4 | -4.434985285 |
| GO Molecular Functions | GO:0072341 | modified amino acid binding | 5 | -4.132111257 |
| GO Molecular Functions | GO:0004364 | glutathione transferase activity | 3 | -3.568943776 |
| GO Molecular Functions | GO:0033293 | monocarboxylic acid binding | 4 | -3.259521956 |
| GO Molecular Functions | GO:0016765 | transferase activity, transferring alkyl or aryl (other than methyl) groups | 3 | -2.591613046 |
| GO Molecular Functions | GO:0031406 | carboxylic acid binding | 5 | -2.383998327 |
| GO Molecular Functions | GO:0043177 | organic acid binding | 5 | -2.288877778 |
| GO Molecular Functions | GO:0032813 | tumor necrosis factor receptor superfamily binding | 5 | -5.518844322 |
| GO Molecular Functions | GO:0097199 | cysteine-type endopeptidase activity involved in apoptotic signaling pathway | 3 | -4.930600751 |
| GO Molecular Functions | GO:0005164 | tumor necrosis factor receptor binding | 4 | -4.883897344 |
| GO Molecular Functions | GO:0097200 | cysteine-type endopeptidase activity involved in execution phase of apoptosis | 3 | -4.557884878 |
| GO Molecular Functions | GO:0097153 | cysteine-type endopeptidase activity involved in apoptotic process | 3 | -4.359217275 |
| GO Molecular Functions | GO:0030235 | nitric-oxide synthase regulator activity | 3 | -5.461242321 |
| GO Molecular Functions | GO:0051117 | ATPase binding | 6 | -5.431334431 |
| GO Molecular Functions | GO:0019838 | growth factor binding | 7 | -5.410912182 |
| GO Molecular Functions | GO:0004714 | transmembrane receptor protein tyrosine kinase activity | 3 | -2.470037397 |
| GO Molecular Functions | GO:0019199 | transmembrane receptor protein kinase activity | 3 | -2.154507941 |
| GO Molecular Functions | GO:0004935 | adrenergic receptor activity | 3 | -5.084012469 |
| GO Molecular Functions | GO:1901338 | catecholamine binding | 3 | -4.454638559 |
| GO Molecular Functions | GO:0008227 | G protein-coupled amine receptor activity | 4 | -4.015400889 |
| GO Molecular Functions | GO:0042826 | histone deacetylase binding | 6 | -4.760748193 |
| GO Molecular Functions | GO:0035035 | histone acetyltransferase binding | 3 | -3.521210511 |
| GO Molecular Functions | GO:0047485 | protein N-terminus binding | 4 | -2.788065143 |
| GO Molecular Functions | GO:0002039 | p53 binding | 3 | -2.432258359 |
| GO Molecular Functions | GO:0070888 | E-box binding | 4 | -4.049303022 |
| GO Molecular Functions | GO:0051087 | chaperone binding | 5 | -3.879703295 |
| GO Molecular Functions | GO:0005507 | copper ion binding | 3 | -2.5496225 |
| GO Molecular Functions | GO:0001618 | virus receptor activity | 4 | -3.344136893 |
| GO Molecular Functions | GO:0104005 | hijacked molecular function | 4 | -3.344136893 |
| GO Molecular Functions | GO:0070851 | growth factor receptor binding | 5 | -3.267343038 |
| GO Molecular Functions | GO:0097718 | disordered domain specific binding | 3 | -3.19472315 |
| GO Molecular Functions | GO:0051219 | phosphoprotein binding | 4 | -3.160052019 |
| GO Molecular Functions | GO:0005080 | protein kinase C binding | 3 | -2.657644476 |
| GO Molecular Functions | GO:0097110 | scaffold protein binding | 3 | -2.5496225 |
| GO Molecular Functions | GO:0016247 | channel regulator activity | 4 | -2.271115437 |
