# Supplementary material for: Exploring the Molecular Mechanism of Liuwei Dihuang Pills for Treating Diabetic Nephropathy by Combined Network Pharmacology and Molecular Docking
Source: Evid Based Complement Alternat Med. 2021 Sep 13;2021:7262208. doi: 10.1155/2021/7262208 (PMC8452392; doi:10.1155/2021/7262208)
Supplement: Supplementary Materials — Table S1: 186 putative targets of LDP. Table S2: 3701 disease targets of DN. Table S3: 131 common targets of LDP and DN. Table S4: GO functional enrichment analysis. Table S5: KEGG pathway enrichment analysis. Figures S1–S4: heatmap of the enrichment analysis results downloaded from Metascape online platform. [file 7262208.f1.zip › 7262208.f1/Table S5 KEGG pathway enrichment analysis.docx]

Table S5: KEGG pathway enrichment analysis

| Category | GO | Description | #GeneInGOAndHitList | _LogP_MyList |
| --- | --- | --- | --- | --- |
| KEGG Pathway | hsa05200 | Pathways in cancer | 57 | -60.1948455 |
| KEGG Pathway | hsa05215 | Prostate cancer | 28 | -41.71513072 |
| KEGG Pathway | hsa05167 | kaposi sarcoma-associated herpesvirus infection | 31 | -38.2335546 |
| KEGG Pathway | hsa05161 | Hepatitis B | 30 | -37.79218791 |
| KEGG Pathway | hsa05160 | Hepatitis C | 29 | -36.67407252 |
| KEGG Pathway | hsa05163 | human cytomegalovirus infection | 31 | -35.21788248 |
| KEGG Pathway | ko05215 | Prostate cancer | 23 | -33.7679222 |
| KEGG Pathway | hsa05212 | Pancreatic cancer | 21 | -30.4945874 |
| KEGG Pathway | hsa05169 | Epstein-Barr virus infection | 28 | -27.7327828 |
| KEGG Pathway | hsa05225 | hepatocellular carcinoma | 24 | -27.37894029 |
| KEGG Pathway | hsa01522 | Endocrine resistance | 20 | -27.0468938 |
| KEGG Pathway | ko01522 | Endocrine resistance | 20 | -27.0468938 |
| KEGG Pathway | ko05212 | Pancreatic cancer | 18 | -26.98549418 |
| KEGG Pathway | ko05219 | Bladder cancer | 16 | -26.71828333 |
| KEGG Pathway | hsa05219 | Bladder cancer | 16 | -26.1166608 |
| KEGG Pathway | hsa05205 | Proteoglycans in cancer | 24 | -25.43731467 |
| KEGG Pathway | hsa05166 | Human T-cell leukemia virus 1 infection | 26 | -25.0325797 |
| KEGG Pathway | ko05205 | Proteoglycans in cancer | 23 | -24.6657555 |
| KEGG Pathway | hsa05165 | human papillomavirus infection | 27 | -24.38432923 |
| KEGG Pathway | hsa05224 | Breast cancer | 21 | -23.78564683 |
| KEGG Pathway | hsa05210 | Colorectal cancer | 18 | -23.63741268 |
| KEGG Pathway | ko05224 | Breast cancer | 20 | -23.28051594 |
| KEGG Pathway | hsa05222 | Small cell lung cancer | 18 | -23.19991259 |
| KEGG Pathway | ko05166 | HTLV-I infection | 23 | -22.31437797 |
| KEGG Pathway | hsa04151 | PI3K-Akt signaling pathway | 26 | -22.19566667 |
| KEGG Pathway | ko04151 | PI3K-Akt signaling pathway | 24 | -20.73128437 |
| KEGG Pathway | hsa05223 | Non-small cell lung cancer | 15 | -20.54182238 |
| KEGG Pathway | ko05210 | Colorectal cancer | 14 | -19.76726844 |
| KEGG Pathway | hsa05220 | Chronic myeloid leukemia | 15 | -19.42178728 |
| KEGG Pathway | ko05222 | Small cell lung cancer | 15 | -19.25317469 |
| KEGG Pathway | hsa05213 | Endometrial cancer | 14 | -19.02774601 |
| KEGG Pathway | ko05220 | Chronic myeloid leukemia | 14 | -18.6436499 |
| KEGG Pathway | ko05206 | MicroRNAs in cancer | 21 | -18.14524519 |
| KEGG Pathway | ko05223 | Non-small cell lung cancer | 13 | -18.1309581 |
| KEGG Pathway | hsa05214 | Glioma | 14 | -18.02687513 |
| KEGG Pathway | hsa05206 | MicroRNAs in cancer | 21 | -17.90927028 |
| KEGG Pathway | hsa05226 | gastric cancer | 17 | -17.68561822 |
| KEGG Pathway | ko05214 | Glioma | 13 | -17.52177628 |
| KEGG Pathway | ko05169 | Epstein-Barr virus infection | 18 | -17.4601422 |
| KEGG Pathway | ko05213 | Endometrial cancer | 12 | -17.16271008 |
| KEGG Pathway | hsa04068 | foxo signaling pathway | 13 | -13.03685562 |
| KEGG Pathway | hsa05218 | Melanoma | 11 | -12.72951943 |
| KEGG Pathway | ko05218 | Melanoma | 10 | -12.01956343 |
| KEGG Pathway | ko05418 | Fluid shear stress and atherosclerosis | 30 | -40.91066496 |
| KEGG Pathway | hsa05418 | Fluid shear stress and atherosclerosis | 30 | -40.31802983 |
| KEGG Pathway | ko04933 | AGE-RAGE signaling pathway in diabetic complications | 27 | -40.11656117 |
| KEGG Pathway | hsa04933 | AGE-RAGE signaling pathway in diabetic complications | 27 | -39.07813116 |
| KEGG Pathway | ko05146 | Amoebiasis | 11 | -12.01430591 |
| KEGG Pathway | hsa05146 | Amoebiasis | 11 | -11.48739355 |
| KEGG Pathway | ko04657 | IL-17 signaling pathway | 21 | -29.2078542 |
| KEGG Pathway | hsa04657 | IL-17 signaling pathway | 21 | -28.99283005 |
| KEGG Pathway | hsa05162 | Measles | 24 | -28.92477003 |
| KEGG Pathway | hsa04668 | TNF signaling pathway | 22 | -28.8622942 |
| KEGG Pathway | ko04668 | TNF signaling pathway | 21 | -27.71037811 |
| KEGG Pathway | hsa05164 | Influenza A | 23 | -25.79036229 |
| KEGG Pathway | hsa05170 | human immunodeficiency virus 1 infection | 24 | -24.9134716 |
| KEGG Pathway | ko05164 | Influenza A | 22 | -24.72807744 |
| KEGG Pathway | hsa04210 | Apoptosis | 21 | -24.45390811 |
| KEGG Pathway | ko04210 | Apoptosis | 20 | -23.66776844 |
| KEGG Pathway | ko04620 | Toll-like receptor signaling pathway | 17 | -21.08338844 |
| KEGG Pathway | hsa05142 | Chagas disease | 17 | -20.86065799 |
| KEGG Pathway | ko04659 | Th17 cell differentiation | 17 | -20.86065799 |
| KEGG Pathway | hsa04620 | Toll-like receptor signaling pathway | 17 | -20.57424366 |
| KEGG Pathway | hsa04659 | Th17 cell differentiation | 17 | -20.50442723 |
| KEGG Pathway | ko05145 | Toxoplasmosis | 17 | -20.43529804 |
| KEGG Pathway | hsa05145 | Toxoplasmosis | 17 | -20.23190464 |
| KEGG Pathway | ko04064 | NF-kappa B signaling pathway | 16 | -20.08060087 |
| KEGG Pathway | ko05142 | Chagas disease (American trypanosomiasis) | 16 | -19.55624164 |
| KEGG Pathway | ko04380 | Osteoclast differentiation | 17 | -19.35384024 |
| KEGG Pathway | hsa04064 | NF-kappa B signaling pathway | 16 | -19.20559964 |
| KEGG Pathway | hsa04380 | Osteoclast differentiation | 17 | -18.84266269 |
| KEGG Pathway | hsa04625 | c-type lectin receptor signaling pathway | 15 | -17.5852987 |
| KEGG Pathway | ko05168 | Herpes simplex infection | 17 | -16.69566033 |
| KEGG Pathway | ko04660 | T cell receptor signaling pathway | 14 | -16.2419073 |
| KEGG Pathway | hsa05168 | Herpes simplex virus 1 infection | 24 | -15.80226201 |
| KEGG Pathway | hsa04660 | T cell receptor signaling pathway | 14 | -15.60324663 |
| KEGG Pathway | ko04662 | B cell receptor signaling pathway | 12 | -15.17551355 |
| KEGG Pathway | hsa04062 | Chemokine signaling pathway | 16 | -14.70289472 |
| KEGG Pathway | ko04621 | NOD-like receptor signaling pathway | 15 | -14.50547249 |
| KEGG Pathway | hsa04662 | B cell receptor signaling pathway | 12 | -14.4503399 |
| KEGG Pathway | ko04062 | Chemokine signaling pathway | 15 | -14.06276623 |
| KEGG Pathway | hsa04621 | NOD-like receptor signaling pathway | 15 | -13.92223921 |
| KEGG Pathway | ko04658 | Th1 and Th2 cell differentiation | 12 | -13.75901164 |
| KEGG Pathway | hsa04658 | Th1 and Th2 cell differentiation | 12 | -13.4192389 |
| KEGG Pathway | ko05162 | Measles | 13 | -13.16264241 |
| KEGG Pathway | ko05120 | Epithelial cell signaling in Helicobacter pylori infection | 9 | -10.49763822 |
| KEGG Pathway | ko04622 | RIG-I-like receptor signaling pathway | 9 | -10.38058516 |
| KEGG Pathway | hsa05120 | Epithelial cell signaling in Helicobacter pylori infection | 9 | -10.32342496 |
| KEGG Pathway | hsa04622 | RIG-I-like receptor signaling pathway | 9 | -10.26713947 |
| KEGG Pathway | hsa01523 | Antifolate resistance | 7 | -9.99961722 |
| KEGG Pathway | ko01523 | Antifolate resistance | 7 | -9.99961722 |
| KEGG Pathway | hsa05131 | Shigellosis | 7 | -7.495315283 |
| KEGG Pathway | ko04623 | Cytosolic DNA-sensing pathway | 6 | -6.29162161 |
| KEGG Pathway | hsa04623 | Cytosolic DNA-sensing pathway | 6 | -6.250530545 |
| KEGG Pathway | hsa04010 | MAPK signaling pathway | 27 | -25.49727827 |
| KEGG Pathway | ko04010 | MAPK signaling pathway | 23 | -22.35364164 |
| KEGG Pathway | ko04012 | ErbB signaling pathway | 15 | -19.08900061 |
| KEGG Pathway | hsa04012 | ErbB signaling pathway | 15 | -18.47244934 |
| KEGG Pathway | hsa01521 | EGFR tyrosine kinase inhibitor resistance | 14 | -17.94386139 |
| KEGG Pathway | ko01521 | EGFR tyrosine kinase inhibitor resistance | 14 | -17.94386139 |
| KEGG Pathway | ko04510 | Focal adhesion | 18 | -17.53850177 |
| KEGG Pathway | hsa04510 | Focal adhesion | 18 | -16.9343743 |
| KEGG Pathway | ko05231 | Choline metabolism in cancer | 13 | -14.90853796 |
| KEGG Pathway | hsa05231 | Choline metabolism in cancer | 13 | -14.45558892 |
| KEGG Pathway | ko04370 | VEGF signaling pathway | 11 | -14.44944813 |
| KEGG Pathway | hsa04370 | VEGF signaling pathway | 11 | -13.95481663 |
| KEGG Pathway | hsa04014 | Ras signaling pathway | 16 | -13.01257137 |
| KEGG Pathway | ko04921 | Oxytocin signaling pathway | 13 | -12.4499477 |
| KEGG Pathway | hsa04921 | Oxytocin signaling pathway | 13 | -12.12737671 |
| KEGG Pathway | ko04540 | Gap junction | 10 | -10.92905166 |
| KEGG Pathway | hsa04540 | Gap junction | 10 | -10.82955694 |
| KEGG Pathway | ko04912 | GnRH signaling pathway | 10 | -10.7324473 |
| KEGG Pathway | hsa04912 | GnRH signaling pathway | 10 | -10.45438258 |
| KEGG Pathway | hsa04726 | Serotonergic synapse | 10 | -9.609676207 |
| KEGG Pathway | ko04664 | Fc epsilon RI signaling pathway | 8 | -8.965748595 |
| KEGG Pathway | hsa04650 | Natural killer cell mediated cytotoxicity | 10 | -8.678310198 |
| KEGG Pathway | hsa04928 | parathyroid hormone synthesis, secretion and action | 9 | -8.491107555 |
| KEGG Pathway | hsa04664 | Fc epsilon RI signaling pathway | 8 | -8.392675424 |
| KEGG Pathway | ko04650 | Natural killer cell mediated cytotoxicity | 9 | -7.866436293 |
| KEGG Pathway | ko04072 | Phospholipase D signaling pathway | 9 | -7.51318024 |
| KEGG Pathway | hsa04072 | Phospholipase D signaling pathway | 9 | -7.100055006 |
| KEGG Pathway | ko04666 | Fc gamma R-mediated phagocytosis | 7 | -6.612385875 |
| KEGG Pathway | hsa04666 | Fc gamma R-mediated phagocytosis | 7 | -6.158921382 |
| KEGG Pathway | hsa04725 | Cholinergic synapse | 7 | -5.919298687 |
| KEGG Pathway | hsa04611 | Platelet activation | 7 | -5.584657193 |
| KEGG Pathway | ko04015 | Rap1 signaling pathway | 8 | -5.161663334 |
| KEGG Pathway | ko04730 | Long-term depression | 5 | -5.034624507 |
| KEGG Pathway | hsa04730 | Long-term depression | 5 | -4.964216022 |
| KEGG Pathway | hsa04015 | Rap1 signaling pathway | 8 | -4.901724231 |
| KEGG Pathway | ko04720 | Long-term potentiation | 5 | -4.798439214 |
| KEGG Pathway | hsa04720 | Long-term potentiation | 5 | -4.735849997 |
| KEGG Pathway | ko04960 | Aldosterone-regulated sodium reabsorption | 4 | -4.571326191 |
| KEGG Pathway | hsa04960 | Aldosterone-regulated sodium reabsorption | 4 | -4.479170367 |
| KEGG Pathway | ko04723 | Retrograde endocannabinoid signaling | 5 | -3.939682996 |
| KEGG Pathway | ko04916 | Melanogenesis | 5 | -3.939682996 |
| KEGG Pathway | hsa04916 | Melanogenesis | 5 | -3.860133055 |
| KEGG Pathway | ko04724 | Glutamatergic synapse | 5 | -3.692762605 |
| KEGG Pathway | hsa04724 | Glutamatergic synapse | 5 | -3.640207061 |
| KEGG Pathway | ko04270 | Vascular smooth muscle contraction | 5 | -3.57241853 |
| KEGG Pathway | hsa04270 | Vascular smooth muscle contraction | 5 | -3.324298725 |
| KEGG Pathway | ko04810 | Regulation of actin cytoskeleton | 6 | -3.300535439 |
| KEGG Pathway | hsa04810 | Regulation of actin cytoskeleton | 6 | -3.066925687 |
| KEGG Pathway | hsa04723 | Retrograde endocannabinoid signaling | 5 | -2.936871845 |
| KEGG Pathway | hsa04713 | circadian entrainment | 4 | -2.896430809 |
| KEGG Pathway | ko04360 | Axon guidance | 5 | -2.846927706 |
| KEGG Pathway | hsa04360 | Axon guidance | 5 | -2.631881378 |
| KEGG Pathway | hsa04066 | HIF-1 signaling pathway | 18 | -21.66818682 |
| KEGG Pathway | hsa01524 | Platinum drug resistance | 15 | -20.24247868 |
| KEGG Pathway | ko01524 | Platinum drug resistance | 15 | -20.24247868 |
| KEGG Pathway | ko05014 | Amyotrophic lateral sclerosis (ALS) | 10 | -13.41384281 |
| KEGG Pathway | hsa05014 | Amyotrophic lateral sclerosis | 10 | -12.8952456 |
| KEGG Pathway | hsa04215 | Apoptosis - multiple species | 7 | -9.792238012 |
| KEGG Pathway | ko04215 | Apoptosis - multiple species | 7 | -9.792238012 |
| KEGG Pathway | hsa05016 | Huntington disease | 10 | -6.998273991 |
| KEGG Pathway | ko05016 | Huntington's disease | 7 | -4.443160079 |
| KEGG Pathway | hsa04218 | cellular senescence | 19 | -20.12207947 |
| KEGG Pathway | hsa04115 | p53 signaling pathway | 15 | -19.68356409 |
| KEGG Pathway | ko04115 | p53 signaling pathway | 12 | -15.33417166 |
| KEGG Pathway | hsa05203 | Viral carcinogenesis | 15 | -13.11256526 |
| KEGG Pathway | ko05203 | Viral carcinogenesis | 14 | -12.14319178 |
| KEGG Pathway | ko04110 | Cell cycle | 10 | -9.432491116 |
| KEGG Pathway | hsa05216 | Thyroid cancer | 7 | -9.246004132 |
| KEGG Pathway | hsa04110 | Cell cycle | 10 | -9.164597315 |
| KEGG Pathway | hsa04934 | cushing syndrome | 10 | -8.07179363 |
| KEGG Pathway | ko05216 | Thyroid cancer | 5 | -6.646840016 |
| KEGG Pathway | hsa05217 | Basal cell carcinoma | 4 | -3.457024691 |
| KEGG Pathway | ko05160 | Hepatitis C | 17 | -19.29515246 |
| KEGG Pathway | ko04071 | Sphingolipid signaling pathway | 12 | -12.43212536 |
| KEGG Pathway | ko04722 | Neurotrophin signaling pathway | 12 | -12.38767203 |
| KEGG Pathway | hsa04722 | Neurotrophin signaling pathway | 12 | -12.1713522 |
| KEGG Pathway | hsa04071 | Sphingolipid signaling pathway | 12 | -12.0050369 |
| KEGG Pathway | ko04920 | Adipocytokine signaling pathway | 9 | -10.43864688 |
| KEGG Pathway | hsa04920 | Adipocytokine signaling pathway | 9 | -10.26713947 |
| KEGG Pathway | hsa04931 | insulin resistance | 10 | -9.83332763 |
| KEGG Pathway | hsa04910 | Insulin signaling pathway | 11 | -9.725905021 |
| KEGG Pathway | hsa04140 | Autophagy - animal | 10 | -9.164597315 |
| KEGG Pathway | ko04910 | Insulin signaling pathway | 10 | -8.975050604 |
| KEGG Pathway | ko04150 | mTOR signaling pathway | 10 | -8.593547943 |
| KEGG Pathway | hsa04150 | mTOR signaling pathway | 10 | -8.402736377 |
| KEGG Pathway | ko04140 | Autophagy - animal | 9 | -8.012485769 |
| KEGG Pathway | ko04930 | Type II diabetes mellitus | 6 | -7.123941385 |
| KEGG Pathway | hsa04930 | Type II diabetes mellitus | 6 | -6.50828585 |
| KEGG Pathway | ko05140 | Leishmaniasis | 14 | -18.46067419 |
| KEGG Pathway | hsa05140 | Leishmaniasis | 14 | -17.46922984 |
| KEGG Pathway | hsa04932 | Non-alcoholic fatty liver disease | 17 | -17.36887166 |
| KEGG Pathway | ko05152 | Tuberculosis | 17 | -16.94039119 |
| KEGG Pathway | hsa05152 | Tuberculosis | 17 | -16.42085664 |
| KEGG Pathway | ko04932 | Non-alcoholic fatty liver disease (NAFLD) | 14 | -13.94725893 |
| KEGG Pathway | ko05323 | Rheumatoid arthritis | 12 | -13.87770487 |
| KEGG Pathway | hsa05323 | Rheumatoid arthritis | 12 | -13.36483253 |
| KEGG Pathway | ko05133 | Pertussis | 10 | -11.58326857 |
| KEGG Pathway | hsa05133 | Pertussis | 10 | -11.2978223 |
| KEGG Pathway | hsa04217 | necroptosis | 12 | -10.59994547 |
| KEGG Pathway | ko04060 | Cytokine-cytokine receptor interaction | 14 | -10.41411299 |
| KEGG Pathway | hsa04060 | Cytokine-cytokine receptor interaction | 15 | -10.35733098 |
| KEGG Pathway | ko05134 | Legionellosis | 8 | -9.726939616 |
| KEGG Pathway | ko05132 | Salmonella infection | 9 | -9.558401762 |
| KEGG Pathway | hsa05134 | Legionellosis | 8 | -9.473368304 |
| KEGG Pathway | hsa05132 | Salmonella infection | 9 | -9.16634431 |
| KEGG Pathway | hsa05010 | Alzheimer disease | 11 | -8.641312713 |
| KEGG Pathway | ko05321 | Inflammatory bowel disease (IBD) | 7 | -7.634058426 |
| KEGG Pathway | hsa05321 | Inflammatory bowel disease | 7 | -7.540810613 |
| KEGG Pathway | ko05010 | Alzheimer's disease | 8 | -5.822964074 |
| KEGG Pathway | hsa05332 | Graft-versus-host disease | 5 | -5.612211773 |
| KEGG Pathway | hsa04940 | Type I diabetes mellitus | 5 | -5.564990221 |
| KEGG Pathway | ko05332 | Graft-versus-host disease | 4 | -4.39197536 |
| KEGG Pathway | ko04940 | Type I diabetes mellitus | 4 | -4.30924963 |
| KEGG Pathway | ko04640 | Hematopoietic cell lineage | 4 | -2.945488899 |
| KEGG Pathway | hsa04640 | Hematopoietic cell lineage | 4 | -2.701311383 |
| KEGG Pathway | hsa04919 | thyroid hormone signaling pathway | 16 | -18.19451467 |
| KEGG Pathway | hsa05221 | Acute myeloid leukemia | 11 | -13.58000257 |
| KEGG Pathway | hsa04630 | JAK-STAT signaling pathway | 14 | -13.11118216 |
| KEGG Pathway | ko05221 | Acute myeloid leukemia | 10 | -13.06103189 |
| KEGG Pathway | ko05230 | Central carbon metabolism in cancer | 10 | -12.29135559 |
| KEGG Pathway | hsa05230 | Central carbon metabolism in cancer | 10 | -11.46668775 |
| KEGG Pathway | ko04630 | Jak-STAT signaling pathway | 12 | -10.97941773 |
| KEGG Pathway | hsa04371 | Apelin signaling pathway | 9 | -7.51318024 |
| KEGG Pathway | hsa05211 | Renal cell carcinoma | 7 | -6.92542019 |
| KEGG Pathway | ko05211 | Renal cell carcinoma | 6 | -6.210130027 |
| KEGG Pathway | ko04550 | Signaling pathways regulating pluripotency of stem cells | 6 | -4.296492882 |
| KEGG Pathway | hsa04550 | Signaling pathways regulating pluripotency of stem cells | 6 | -4.065178859 |
| KEGG Pathway | hsa04926 | relaxin signaling pathway | 16 | -17.47424362 |
| KEGG Pathway | hsa04915 | Estrogen signaling pathway | 16 | -16.96974905 |
| KEGG Pathway | ko04917 | Prolactin signaling pathway | 11 | -13.58000257 |
| KEGG Pathway | hsa04917 | Prolactin signaling pathway | 11 | -13.2337445 |
| KEGG Pathway | hsa04024 | cAMP signaling pathway | 14 | -11.450287 |
| KEGG Pathway | ko04024 | cAMP signaling pathway | 13 | -10.98137602 |
| KEGG Pathway | ko04915 | Estrogen signaling pathway | 10 | -10.45438258 |
| KEGG Pathway | hsa05202 | Transcriptional misregulation in cancer | 15 | -13.3282507 |
| KEGG Pathway | ko05202 | Transcriptional misregulation in cancer | 14 | -12.80244959 |
| KEGG Pathway | hsa05143 | African trypanosomiasis | 9 | -12.29874541 |
| KEGG Pathway | ko05144 | Malaria | 9 | -11.84518642 |
| KEGG Pathway | ko05143 | African trypanosomiasis | 8 | -11.406725 |
| KEGG Pathway | hsa05144 | Malaria | 9 | -11.36477567 |
| KEGG Pathway | ko04514 | Cell adhesion molecules (CAMs) | 4 | -2.323279622 |
| KEGG Pathway | hsa04514 | Cell adhesion molecules | 4 | -2.240781636 |
| KEGG Pathway | hsa05204 | Chemical carcinogenesis | 10 | -10.87899911 |
| KEGG Pathway | ko05204 | Chemical carcinogenesis | 9 | -9.74725421 |
| KEGG Pathway | ko00980 | Metabolism of xenobiotics by cytochrome P450 | 8 | -8.66654378 |
| KEGG Pathway | hsa00980 | Metabolism of xenobiotics by cytochrome P450 | 8 | -8.572648511 |
| KEGG Pathway | hsanan01 | drug metabolism | 8 | -7.083942634 |
| KEGG Pathway | hsa00982 | Drug metabolism - cytochrome P450 | 6 | -6.017751354 |
| KEGG Pathway | ko00982 | Drug metabolism - cytochrome P450 | 6 | -6.017751354 |
| KEGG Pathway | ko00380 | Tryptophan metabolism | 4 | -4.434985285 |
| KEGG Pathway | hsa00380 | Tryptophan metabolism | 4 | -4.049303022 |
| KEGG Pathway | ko00480 | Glutathione metabolism | 4 | -3.917844574 |
| KEGG Pathway | ko00140 | Steroid hormone biosynthesis | 4 | -3.796510414 |
| KEGG Pathway | hsa00140 | Steroid hormone biosynthesis | 4 | -3.71130793 |
| KEGG Pathway | hsa00480 | Glutathione metabolism | 4 | -3.656991361 |
| KEGG Pathway | ko00830 | Retinol metabolism | 4 | -3.604513957 |
| KEGG Pathway | hsa00830 | Retinol metabolism | 4 | -3.553761472 |
| KEGG Pathway | hsa00591 | Linoleic acid metabolism | 3 | -3.475270311 |
| KEGG Pathway | ko00591 | Linoleic acid metabolism | 3 | -3.475270311 |
| KEGG Pathway | hsa05020 | Prion disease | 7 | -9.598583988 |
| KEGG Pathway | ko05020 | Prion diseases | 7 | -9.598583988 |
| KEGG Pathway | ko04141 | Protein processing in endoplasmic reticulum | 5 | -2.948463378 |
| KEGG Pathway | hsa04141 | Protein processing in endoplasmic reticulum | 5 | -2.913926697 |
| KEGG Pathway | hsa04914 | Progesterone-mediated oocyte maturation | 9 | -8.850403788 |
| KEGG Pathway | hsa04114 | Oocyte meiosis | 9 | -7.953330263 |
| KEGG Pathway | ko04914 | Progesterone-mediated oocyte maturation | 8 | -7.759499879 |
| KEGG Pathway | ko04114 | Oocyte meiosis | 7 | -5.69938427 |
| KEGG Pathway | ko04310 | Wnt signaling pathway | 10 | -8.823844905 |
| KEGG Pathway | hsa04310 | Wnt signaling pathway | 10 | -8.120872875 |
| KEGG Pathway | ko04728 | Dopaminergic synapse | 8 | -6.729098479 |
| KEGG Pathway | hsa04728 | Dopaminergic synapse | 8 | -6.677972005 |
| KEGG Pathway | ko05031 | Amphetamine addiction | 6 | -6.092857103 |
| KEGG Pathway | hsa05031 | Amphetamine addiction | 6 | -5.981066658 |
| KEGG Pathway | ko04670 | Leukocyte transendothelial migration | 7 | -5.94489175 |
| KEGG Pathway | hsa04670 | Leukocyte transendothelial migration | 7 | -5.770687593 |
| KEGG Pathway | hsa04750 | inflammatory mediator regulation of trp channels | 5 | -3.879703295 |
| KEGG Pathway | ko04918 | Thyroid hormone synthesis | 4 | -3.388290631 |
| KEGG Pathway | hsa04918 | Thyroid hormone synthesis | 4 | -3.280234157 |
| KEGG Pathway | ko04972 | Pancreatic secretion | 4 | -2.962217094 |
| KEGG Pathway | ko04961 | Endocrine and other factor-regulated calcium reabsorption | 3 | -2.854789034 |
| KEGG Pathway | hsa04972 | Pancreatic secretion | 4 | -2.848983598 |
| KEGG Pathway | hsa04922 | Glucagon signaling pathway | 4 | -2.788065143 |
| KEGG Pathway | hsa04961 | Endocrine and other factor-regulated calcium reabsorption | 3 | -2.77688461 |
| KEGG Pathway | ko04971 | Gastric acid secretion | 3 | -2.276520999 |
| KEGG Pathway | hsa04971 | Gastric acid secretion | 3 | -2.244710217 |
| KEGG Pathway | ko05032 | Morphine addiction | 3 | -2.044938271 |
| KEGG Pathway | hsa05032 | Morphine addiction | 3 | -2.03200489 |
| KEGG Pathway | hsa04211 | Longevity regulating pathway | 9 | -8.630504393 |
| KEGG Pathway | hsa04213 | Longevity regulating pathway - multiple species | 4 | -3.68391217 |
| KEGG Pathway | ko04213 | Longevity regulating pathway - multiple species | 4 | -3.68391217 |
| KEGG Pathway | ko04152 | AMPK signaling pathway | 5 | -3.589129146 |
| KEGG Pathway | hsa04152 | AMPK signaling pathway | 5 | -3.444044913 |
| KEGG Pathway | ko04137 | Mitophagy - animal | 7 | -7.634058426 |
| KEGG Pathway | hsa04137 | Mitophagy - animal | 7 | -7.540810613 |
| KEGG Pathway | hsa05030 | Cocaine addiction | 4 | -3.886621432 |
| KEGG Pathway | hsa04530 | Tight junction | 5 | -2.80372188 |
| KEGG Pathway | ko05030 | Cocaine addiction | 3 | -2.802278839 |
| KEGG Pathway | ko04530 | Tight junction | 4 | -2.072351701 |
| KEGG Pathway | hsa05416 | Viral myocarditis | 7 | -7.406467719 |
| KEGG Pathway | hsa04913 | Ovarian steroidogenesis | 6 | -6.696753521 |
| KEGG Pathway | ko04913 | Ovarian steroidogenesis | 5 | -5.429596796 |
| KEGG Pathway | ko00590 | Arachidonic acid metabolism | 5 | -4.964216022 |
| KEGG Pathway | hsa00590 | Arachidonic acid metabolism | 5 | -4.929928343 |
| KEGG Pathway | ko04020 | Calcium signaling pathway | 9 | -6.691132513 |
| KEGG Pathway | hsa04923 | Regulation of lipolysis in adipocytes | 6 | -6.50828585 |
| KEGG Pathway | hsa04020 | Calcium signaling pathway | 9 | -6.237557803 |
| KEGG Pathway | ko04022 | cGMP-PKG signaling pathway | 7 | -4.915354209 |
| KEGG Pathway | hsa04022 | cGMP-PKG signaling pathway | 7 | -4.747801209 |
| KEGG Pathway | ko04261 | Adrenergic signaling in cardiomyocytes | 6 | -4.211004361 |
| KEGG Pathway | hsa04261 | Adrenergic signaling in cardiomyocytes | 6 | -4.161287965 |
| KEGG Pathway | ko04080 | Neuroactive ligand-receptor interaction | 7 | -3.470630269 |
| KEGG Pathway | hsa04924 | Renin secretion | 4 | -3.410858862 |
| KEGG Pathway | ko04970 | Salivary secretion | 4 | -3.066820267 |
| KEGG Pathway | hsa04970 | Salivary secretion | 4 | -2.99626089 |
| KEGG Pathway | ko04144 | Endocytosis | 6 | -2.841851498 |
| KEGG Pathway | hsa04144 | Endocytosis | 6 | -2.750890567 |
| KEGG Pathway | hsa04080 | Neuroactive ligand-receptor interaction | 7 | -2.737149604 |
| KEGG Pathway | ko04924 | Renin secretion | 3 | -2.450988897 |
| KEGG Pathway | hsa04610 | Complement and coagulation cascades | 7 | -6.678947463 |
| KEGG Pathway | ko04610 | Complement and coagulation cascades | 6 | -5.706359949 |
| KEGG Pathway | hsa04976 | Bile secretion | 5 | -4.559274925 |
| KEGG Pathway | ko04976 | Bile secretion | 4 | -3.457024691 |
| KEGG Pathway | hsa05330 | Allograft rejection | 4 | -4.155575546 |
| KEGG Pathway | ko04350 | TGF-beta signaling pathway | 4 | -3.179422784 |
| KEGG Pathway | ko05330 | Allograft rejection | 3 | -3.125248435 |
| KEGG Pathway | hsa04350 | TGF-beta signaling pathway | 4 | -3.013585002 |
| KEGG Pathway | hsa05322 | Systemic lupus erythematosus | 4 | -2.302166099 |
| KEGG Pathway | ko04612 | Antigen processing and presentation | 3 | -2.244710217 |
| KEGG Pathway | hsa04612 | Antigen processing and presentation | 3 | -2.169028672 |
| KEGG Pathway | hsa04340 | Hedgehog signaling pathway | 4 | -4.083944805 |
| KEGG Pathway | ko04340 | Hedgehog signaling pathway | 3 | -2.854789034 |
| KEGG Pathway | ko04390 | Hippo signaling pathway | 4 | -2.220943268 |
| KEGG Pathway | hsa04390 | Hippo signaling pathway | 4 | -2.037768235 |
| KEGG Pathway | hsa05012 | Parkinson disease | 6 | -3.719214449 |
| KEGG Pathway | hsa04714 | thermogenesis | 5 | -2.035578953 |
| KEGG Pathway | ko03320 | PPAR signaling pathway | 4 | -3.433766973 |
| KEGG Pathway | hsa03320 | PPAR signaling pathway | 4 | -3.14093035 |
| KEGG Pathway | ko04974 | Protein digestion and absorption | 4 | -3.066820267 |
| KEGG Pathway | hsa04974 | Protein digestion and absorption | 4 | -2.99626089 |
| KEGG Pathway | hsa00051 | Fructose and mannose metabolism | 3 | -2.938249299 |
| KEGG Pathway | ko04973 | Carbohydrate digestion and absorption | 3 | -2.938249299 |
| KEGG Pathway | hsa04973 | Carbohydrate digestion and absorption | 3 | -2.703874406 |
| KEGG Pathway | hsa04216 | Ferroptosis | 3 | -2.881955357 |
| KEGG Pathway | ko04520 | Adherens junction | 3 | -2.326037108 |
| KEGG Pathway | hsa04520 | Adherens junction | 3 | -2.183747735 |
