# Supplementary figures and images for: Exploring the Molecular Mechanism of Liuwei Dihuang Pills for Treating Diabetic Nephropathy by Combined Network Pharmacology and Molecular Docking
Source: Evid Based Complement Alternat Med. 2021 Sep 13;2021:7262208. doi: 10.1155/2021/7262208 (PMC8452392; doi:10.1155/2021/7262208)

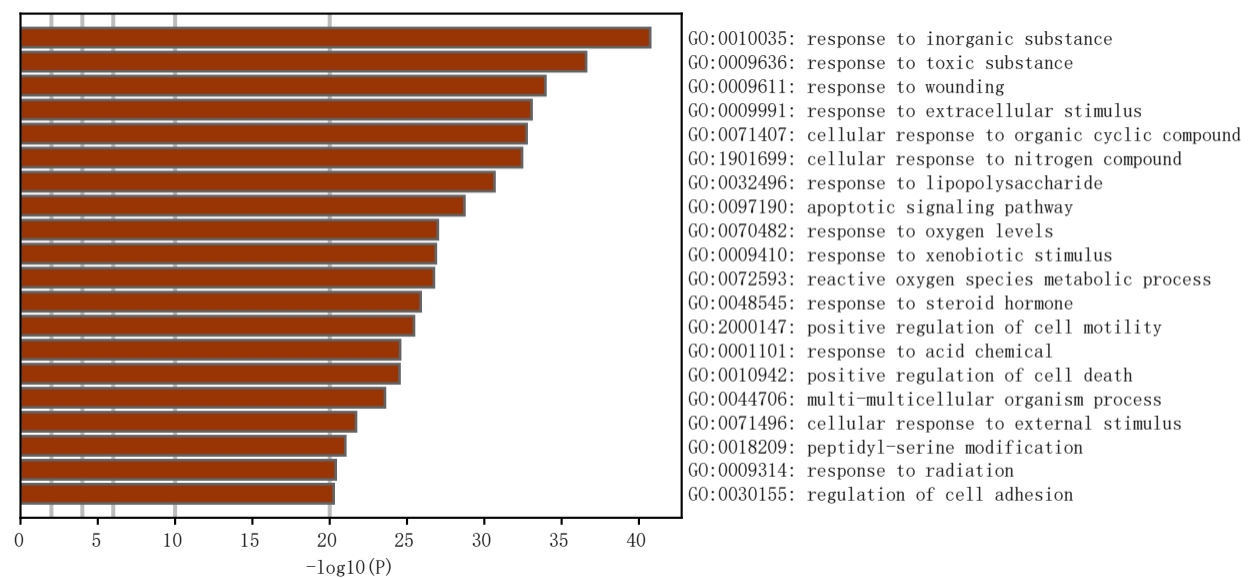

Supplement: Supplementary Materials — Table S1: 186 putative targets of LDP. Table S2: 3701 disease targets of DN. Table S3: 131 common targets of LDP and DN. Table S4: GO functional enrichment analysis. Table S5: KEGG pathway enrichment analysis. Figures S1–S4: heatmap of the enrichment analysis results downloaded from Metascape online platform. [file 7262208.f1.zip › 7262208.f1/Figure S1 Heatmap of GO BP downloaded from Metascape.pdf]

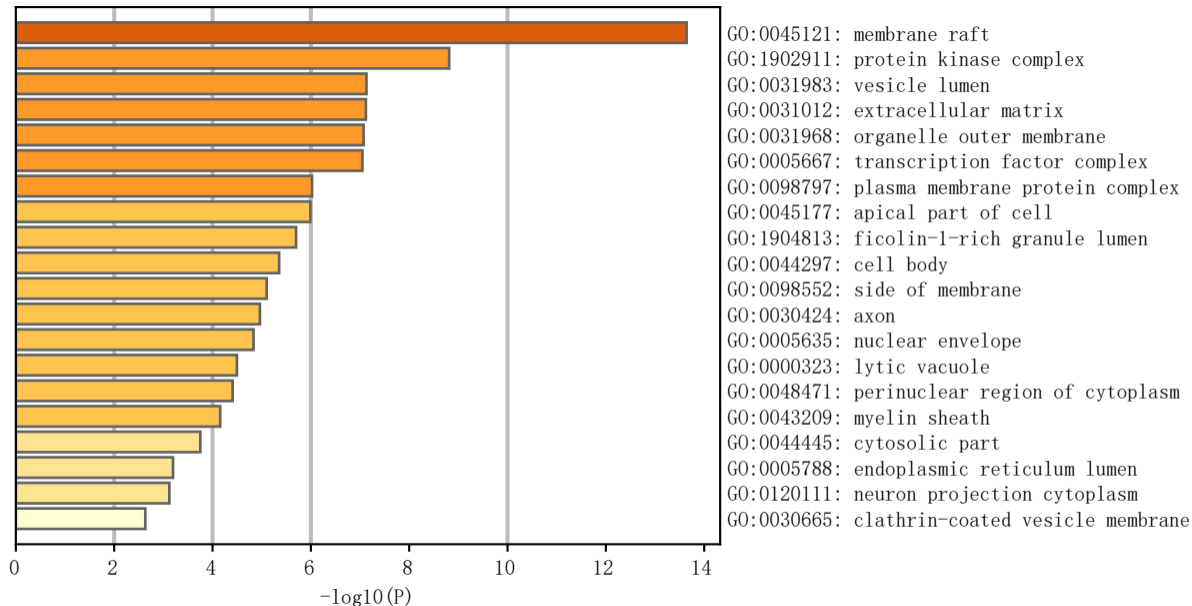

Supplement: Supplementary Materials — Table S1: 186 putative targets of LDP. Table S2: 3701 disease targets of DN. Table S3: 131 common targets of LDP and DN. Table S4: GO functional enrichment analysis. Table S5: KEGG pathway enrichment analysis. Figures S1–S4: heatmap of the enrichment analysis results downloaded from Metascape online platform. [file 7262208.f1.zip › 7262208.f1/Figure S2 Heatmap of GO CC downloaded from Metascape.pdf]

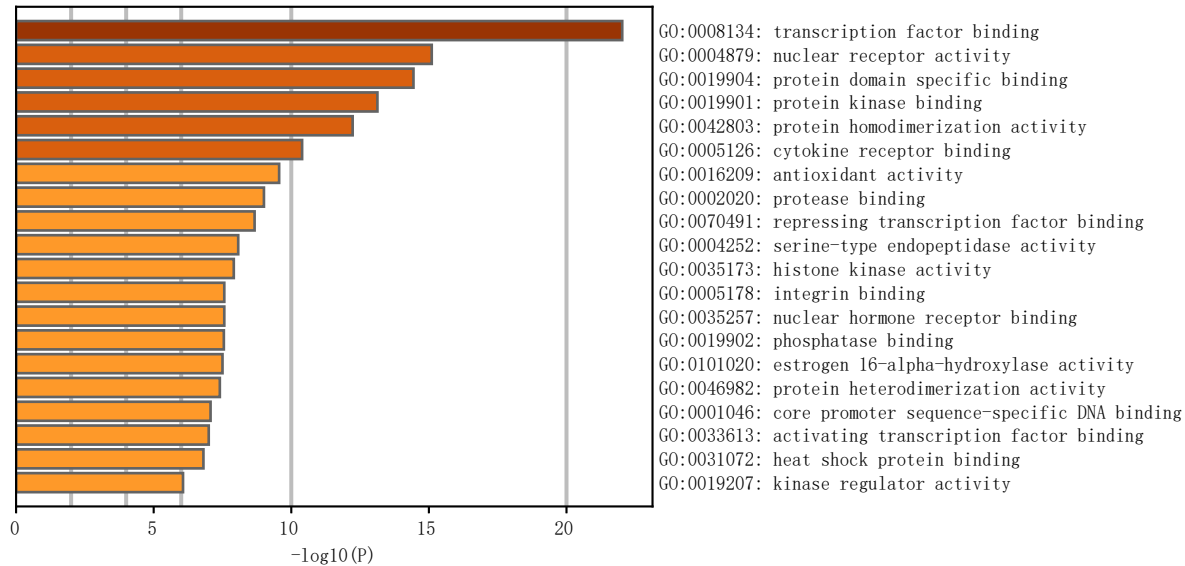

Supplement: Supplementary Materials — Table S1: 186 putative targets of LDP. Table S2: 3701 disease targets of DN. Table S3: 131 common targets of LDP and DN. Table S4: GO functional enrichment analysis. Table S5: KEGG pathway enrichment analysis. Figures S1–S4: heatmap of the enrichment analysis results downloaded from Metascape online platform. [file 7262208.f1.zip › 7262208.f1/Figure S3 Heatmap of GO MF downloaded from Metascape.pdf]

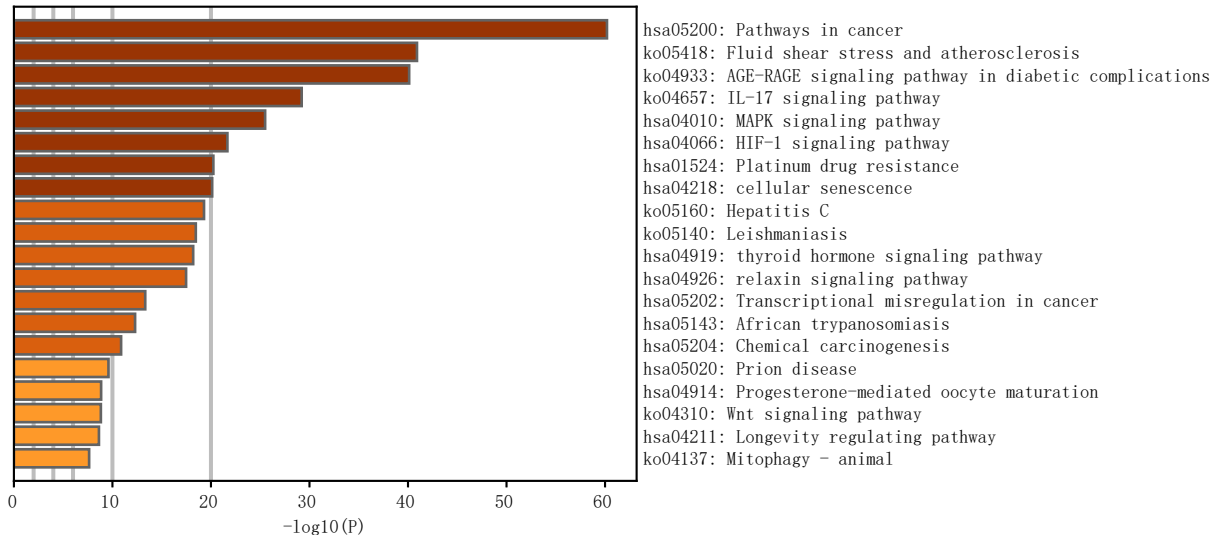

Supplement: Supplementary Materials — Table S1: 186 putative targets of LDP. Table S2: 3701 disease targets of DN. Table S3: 131 common targets of LDP and DN. Table S4: GO functional enrichment analysis. Table S5: KEGG pathway enrichment analysis. Figures S1–S4: heatmap of the enrichment analysis results downloaded from Metascape online platform. [file 7262208.f1.zip › 7262208.f1/Figure S4 Heatmap of KEGG pathways downloaded from Metascape.pdf]
